# Menthyl esterification allows chiral resolution for the synthesis of artificial glutamate analogs

**DOI:** 10.3762/bjoc.17.48

**Published:** 2021-02-24

**Authors:** Kenji Morokuma, Shuntaro Tsukamoto, Kyosuke Mori, Kei Miyako, Ryuichi Sakai, Raku Irie, Masato Oikawa

**Affiliations:** 1 Yokohama City University, Seto 22-2, Kanazawa-ku, Yokohama 236-0027, Japan; 2 Faculty of Fisheries Sciences, Hokkaido University, Hakodate 041-8611, Japan

**Keywords:** chiral resolution, configurational analysis, glutamate, metathesis, neuroactivity

## Abstract

Herein, we report the enantiospecific synthesis of two artificial glutamate analogs designed based on IKM-159, an antagonist selective to the AMPA-type ionotropic glutamate receptor. The synthesis features the chiral resolution of the carboxylic acid intermediate by the esterification with ʟ-menthol, followed by a configurational analysis by NMR, conformational calculation, and X-ray crystallography. A mice in vivo assay showed that (2*R*)-MC-27, with a six-membered oxacycle, is neuroactive, whereas the (2*S*)-counterpart is inactive. It was also found that TKM-38, with an eight-membered azacycle, is neuronally inactive, showing that the activity is controlled by the ring C.

## Introduction

**Figure 1:**
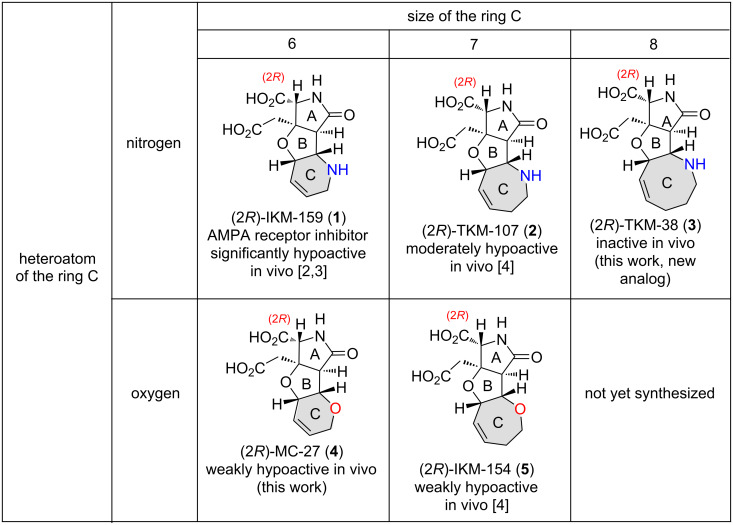
Artificial glutamate analogs synthesized in an enantiomerically pure form.

The ionotropic glutamate receptor (iGluR) mediates the majority of the excitatory neurotransmission in the mammalian central nervous system (CNS) and plays an important role in higher brain functions, such as learning and memory [1]. Previously, we have synthetically developed (2*R*)-IKM-159 (**1**) as an artificial glutamate analog that is selectively antagonistic to AMPA-type iGluR (Figure 1) [2–3]. From a series of these studies (see **1**, **2**, and **5** in Figure 1) [3–4], we found that 1) the (2*R*)-enantiomer is responsible for the neuroactivity and that 2) the activity is controlled by the structure of the ring C; the seven-membered azacyclic analog (2*R*)-TKM-107 (**2**) is moderately hypoactive, that is, it attenuates the voluntary movement of mice upon intracerebroventricular injection, and the seven-membered oxacycle (2*R*)-IKM-154 (**5**) is weakly hypoactive [4].

In 2015, we reported the synthesis and evaluation of the six-membered oxacyclic analog MC-27 in the racemic form, (*rac*)-**4**, which was shown to be weakly hypoactive in vivo [5]. In the present study, we synthesized both enantiomers of MC-27, **4** and 4*, separately (see Figure 1 for the (2*R*)-enantiomer) and found that, as expected, the (2*R*)-enantiomer **4** is responsible for the neuroactivity (see above). Herein, we also report the enantiospecific synthesis and evaluation of the novel eight-membered azacyclic analog (2*R*)-TKM-38 (**3**, see Figure 1) and the antipode 3*.

For the synthesis of enantiomerically pure artificial glutamate analogs, we have previously developed an enantiospecific synthesis using a chiral amine as a starting material and have applied this to the synthesis of both enantiomers of IKM-159 (**1** and the antipode 1*) in 2013 [3]. The synthesis reported herein is based on the menthol-mediated chiral resolution, which was developed de novo thereafter for the enantiospecific synthesis of the seven-membered-ring analogs TKM-107 (**2** and the antipode 2*, see Figure 1) and IKM-154 (**5** and the antipode 5*) [4]. The structural analysis of the diastereomeric menthyl esters obtained after chiral resolution was conducted based on a combination of NOESY data and conformational calculation in that study [4]. In this study, the configurational analysis of the menthyl ester is reasonably justified by X-ray crystallographic analysis and the PGME amide analysis, as follows.

## Results and Discussion

### Enantiospecific synthesis of MC-27

**Scheme 1:**
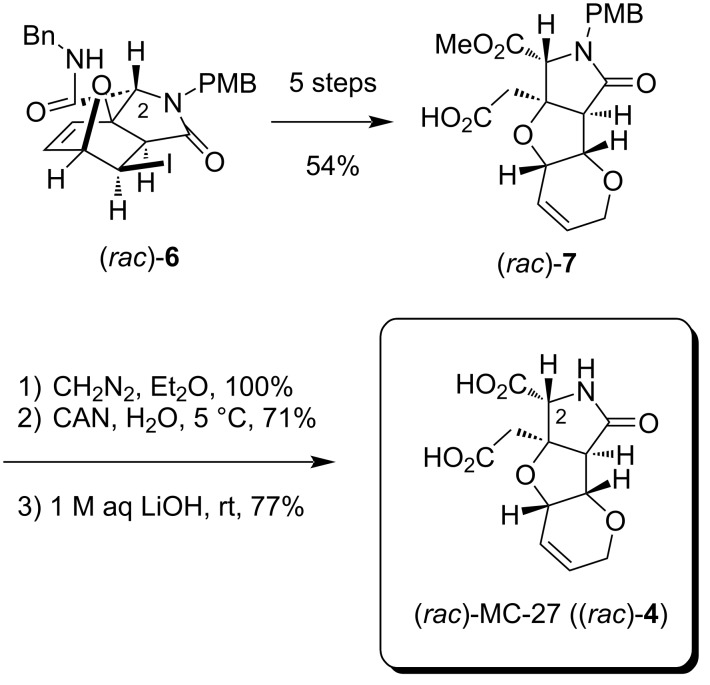
Our established synthetic route to racemic MC-27 ((*rac*)-**4**) [5–7].

The synthetic route to the racemate of the heterotricyclic compound MC-27, (*rac*)-**4**, has been established in 2015 as shown in Scheme 1 [5–7]. Starting from the oxanorbornene (*rac*)-**6** [6,8], the heterotricyclic framework was constructed over a five-step sequence including the domino metathesis reaction as a key step to give (*rac*)-**7**. The subsequent three steps from (*rac*)-**7** (esterification with CH_2_N_2_, PMB removal, and ester hydrolysis) had been proven to be promising for the preparation of subgram quantities of (*rac*)-MC-27 ((*rac*)-**4**), which was found to cause weak inhibition of the voluntary movement of mice upon intracerebroventricular injection [5].

**Scheme 2:**
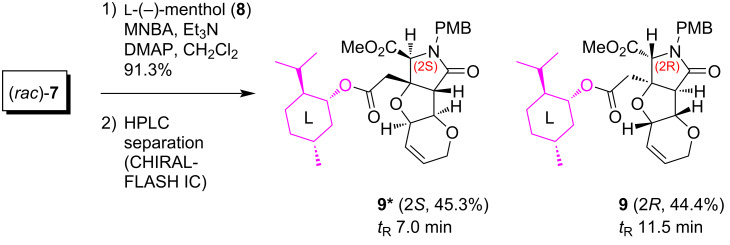
Resolution of the MC-27 precursor (*rac*)-**7** by a chiral auxiliary.

**Figure 2:**
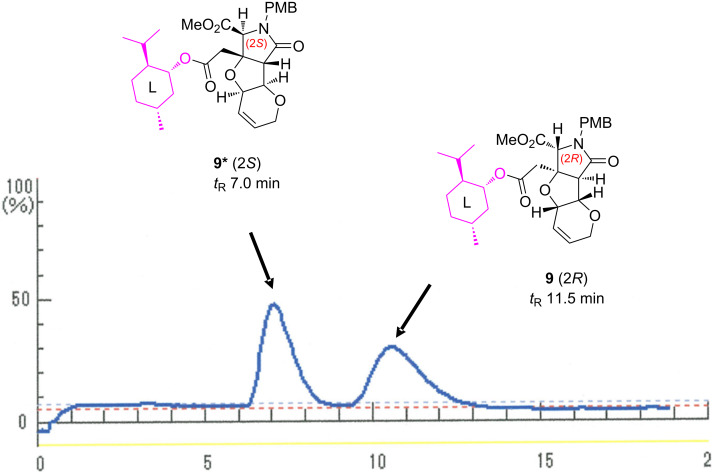
Chiral chromatography profiles for the separation of menthyl ester diastereomers **9** and 9*. Conditions: 30 × 100 mm CHIRALFLASH IC column, EtOH/hexane 65:35, 20 mL/min, 25 °C, 254 nm, *t*_R_ 7.0, 11.5 min.

On the basis of the racemate synthesis shown in Scheme 1, in the present study, we envisioned that both enantiomers of MC-27 could independently be synthesized from the racemic carboxylic acid intermediate (*rac*)-**7** [6]. For such a chiral resolution, we recently discovered that ʟ-(−)-menthol (**8**) is of use as a chiral auxiliary in the enantiospecific synthesis of the other analogs **2** and **5** (see Figure 1) [4], and the strategy was found to also be effective here (Scheme 2). Thus, the esterification mediated by 2-methyl-6-nitrobenzoic anhydride (MNBA, Shiina esterification) [9], followed by chromatographic separation of the diastereomers, successfully generated the (2*S*)-isomer 9* (*t*_R_ 7.0 min) and the (2*R*)-isomer **9** (*t*_R_ 11.5 min) in 45.3% and 44.4% yield, respectively (Figure 2). It should be noted here that DCC and DMAP drove the esterification in a poor yield (51% in total for **9** and 9*). As shown in Figure 2, the preparative separation was performed cleanly even on a gram-scale synthesis (1.70 g). The structures, illustrated in Scheme 2 and Figure 2, were unambiguously determined later from crystallographic and spectroscopic studies of the 2*R*-derivative **9** (see below).

**Scheme 3:**
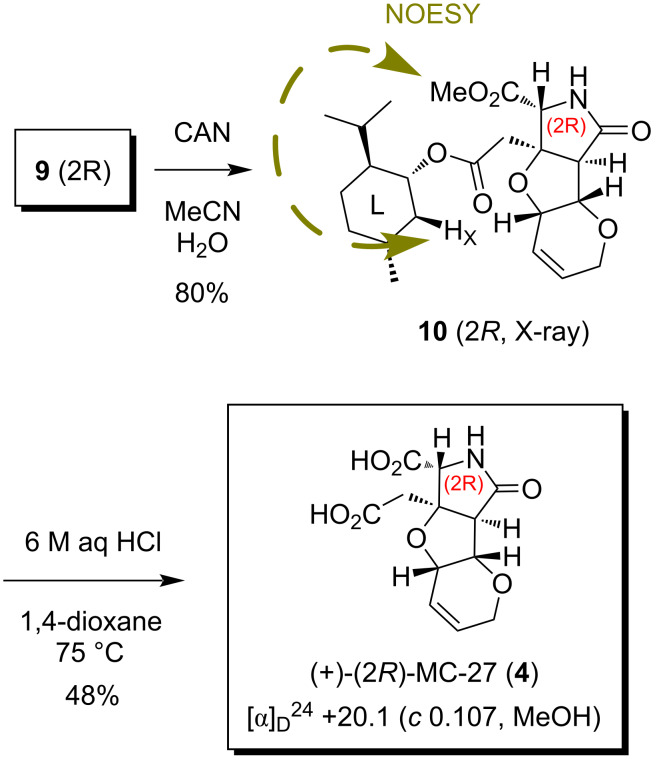
Final elaboration of (2*R*)-MC-27 (**4**).

**Figure 3:**
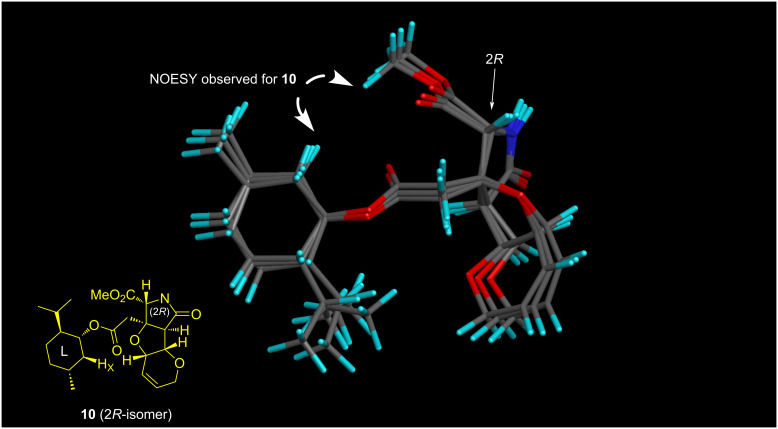
Superimposed structures of the top 3 stable conformers (76.5% total population) generated by CONFLEX (MMFF94S) for the (2*R*)-isomer, which is consistent with the NOESY crosspeak observed for **10** (400 MHz, CDCl_3_). See Supporting Information File 1 for the stereo diagram.

**Figure 4:**
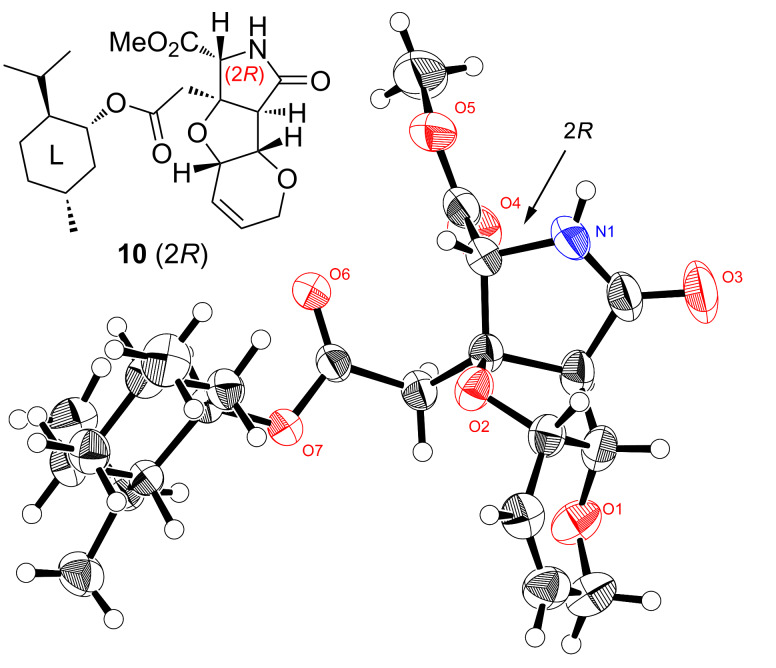
Crystallographic analysis of the menthyl ester **10**, unequivocally showing the 2*R* configuration (CCDC 2030829).

Scheme 3 shows the synthesis of the (2*R*)-enantiomer of MC-27, **4**. The deprotection of the PMB group of **9** (*t*_R_ 11.5 min in Figure 2) by CAN proceeded smoothly at rt to give rise to **10**. The stereochemical configuration of **10** was determined to be 2*R*, in consideration of the fact that a NOESY crosspeak at CO_2_Me/H_x_ observed for **10** was consistent with the top three conformers (total population: 76.5%) for the (2*R*)-isomer generated by CONFLEX (Version 5, MMFF94S, Figure 3) [10–12]. Since **10** was obtained as crystals, the configurational analysis was thereafter confirmed by single-crystal X-ray analysis, as shown in Figure 4. The conformational differences for **10** between the results of the calculations and the analysis of the NMR data compared to the situation in the crystal are arising from the four contiguous single bonds between the heterocycle and the menthyl group. These take a stable extended conformation in the calculations and in the NMR experiment, whereas a rather folded conformation is taken in the crystals. Such a discrepancy is often observed and reported in small molecules and biomacromolecule due to intermolecular or intramolecular H-bonds and/or hydrophobic interactions [13].

Hydrolytic deprotections were finally examined to complete the synthesis. The previously employed alkaline hydrolysis (1 M aq LiOH, MeOH or THF, rt→45 °C) [4], however, gave only a monocarboxylic acid product, and the menthyl ester remained unaffected (structure not shown). Fortunately, the complete deprotection of the two esters was cleanly possible under acidic conditions (6 M aq HCl, 1,4-dioxane, 75 °C, 4 days) to furnish (2*R*)-MC-27 (**4**) in 48% yield (Scheme 3), the chromatographic behavior and the spectroscopic data (^1^H and ^13^C NMR) of which were identical to those of the racemate [5].

**Scheme 4:**
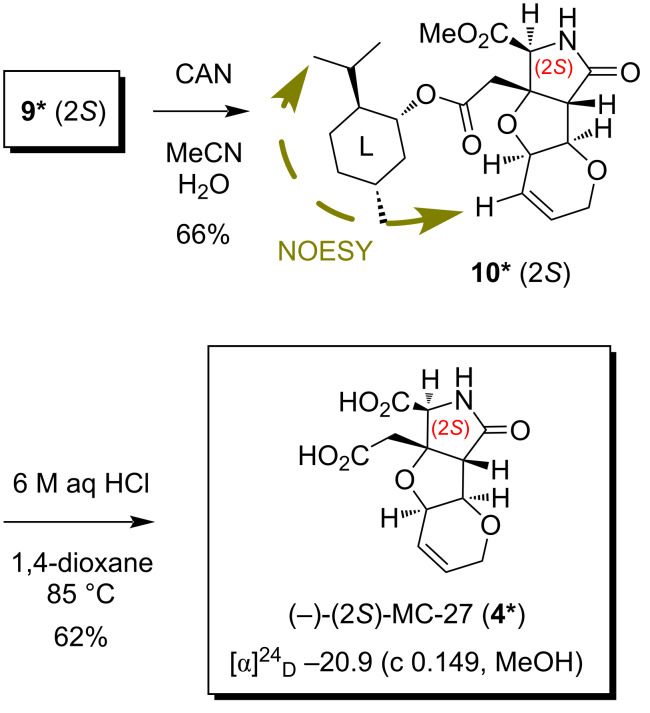
Synthesis of (2*S*)-MC-27 (4*) from 9*.

On the other hand, the *N*-PMB amide 9* (2*S*, *t*_R_ 7.0 min in Figure 2) was also deprotected by CAN (Scheme 4), and the configurational analysis of the product 10* was attempted separately. An important NOESY correlation observed for 10* is also shown in Scheme 4. The conformational analysis of the (2*S*)-isomer carried out by CONFLEX (MMFF94S), however, was not very encouraging since the four contiguous single bonds between the heterotricycle and the menthyl group were found to be freely rotating in the top five conformers (total population: 94.4%, data not shown, see below for detailed discussions). Although the characteristic NOESY crosspeak shown in Scheme 4 seemed to be attributable to the second conformer of the (2*S*)-isomer (29.4% population, data not shown), the other conformers were not consistent well. Due to the conformational flexibility of the (2*S*)-isomer thus presumed, the de novo configurational characterization by spectroscopic analysis, in combination with conformational calculations, was unsuccessful to conclude that 10* is the (2*S*)-isomer, separately.

With 10* (2*S*) in hand, (2*S*)-MC-27 (4*) was synthesized in a reasonable yield (62%) by acidic hydrolysis (Scheme 4). The chromatographic behavior and the spectroscopic data (^1^H and ^13^C NMR) of (2*S*)-MC-27 (4*) thus synthesized were identical to those of the antipode **4** (see above as well as Scheme 3 and Scheme 4).

### Enantiospecific synthesis of TKM-38

**Scheme 5:**
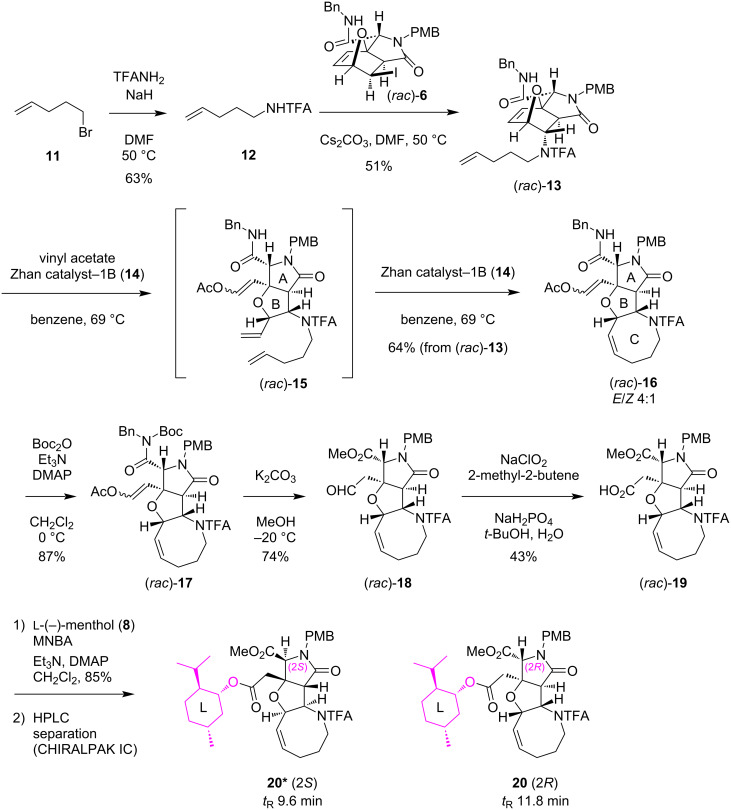
Construction and chiral resolution of the 5/5/8-ring system towards the TKM-38 enantiomers.

**Figure 5:**
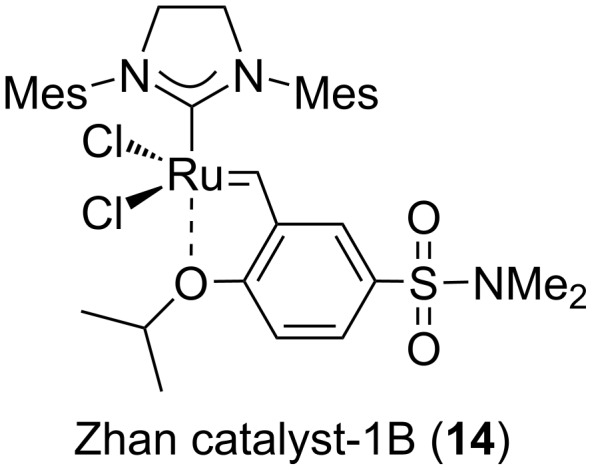
Structure of Zhan catalyst-1B (**14**) [14].

For the enantiospecific synthesis of TKM-38, which uniquely bears an eight-membered azacycle as the ring C, we explored 1) the amino-protecting group and 2) the conditions for the cyclization of the medium-sized ring by ring-closing metathesis (RCM). Finally, the established synthetic route with the optimized reaction conditions is shown in Scheme 5. First, the direct introduction of a pentenyl group to the oxanorbornene (*rac*)-**6** [6,8] proceeds smoothly to give (*rac*)-**13** in a moderate yield (51%) when 2,2,2-trifluoro-*N*-(pent-4-en-1-yl)acetamide (**12**), prepared from 4-pentenyl bromide (**11**) and TFANH_2_, was reacted in the presence of Cs*_2_*CO_3_ in DMF. We next examined the construction of the characteristic eight-membered ring using vinyl acetate and Zhan catalyst-1B (**14**, see Figure 5 for the structure) [14]. The construction of such a medium-sized ring is generally highly challenging [15–16], and this was also the case for (*rac*)-**13**, since we first obtained the incomplete triene intermediate (*rac*)-**15** as a result of only ring-opening metathesis (ROM) mediated by the Fischer carbene complex [Ru]=CH–OAc [6], generated by the reaction of Zhan catalyst-1B (**14**) with vinyl acetate. The predominant generation of triene (*rac*)-**15** obviously indicated that the ROM reaction proceeded regioselectively, as also observed in our previous study [6]. With triene (*rac*)-**15** in hand, the cyclization of the eight-membered ring was furthermore attempted by RCM. Gratifyingly, after several trial experiments, we found that the desired cyclization took place smoothly to give rise to heterotricycle (*rac*)-**16** in 64% yield (over two steps) as a 4:1 mixture of the *E*/*Z* isomers at the acetoxyalkene moiety, when the reaction was conducted with 0.05 equiv of catalyst **14** at 69 °C. The highly efficient overall conversion of oxanorbornene (*rac*)-**13** to heterotricycle (*rac*)-**16** through the eight-membered-ring formation would be owing to the *cis*-relationships of the pentenyl and vinyl groups on the ring B of (*rac*)-**15**, which allows the proximal arrangement of the reacting sites in the RCM.

**Figure 6:**
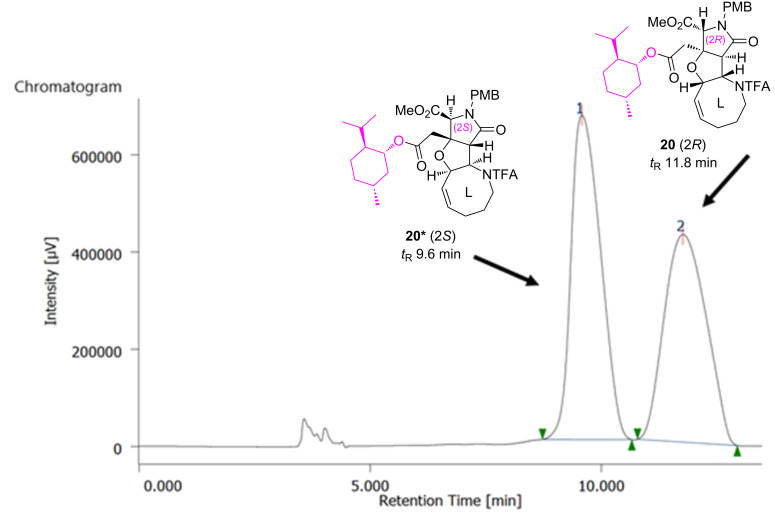
Chiral HPLC profiles for the separation of menthyl ester diastereomers 20* and **20**. Conditions: 4.6 × 250 mm CHIRALPAK IC column, EtOH/hexane 1:19, 1 mL/min, 40 °C, 254 nm, *t*_R_ 9.6, 11.8 min.

The *N*-Boc derivatization of (*rac*)-**16** (87% yield), followed by alkaline methanolysis (74% yield) [17] and Pinnick oxidation (43% yield) [18–20], delivered carboxylic acid (*rac*)-**19**. Unfortunately, an attempt to improve the oxidation yield was not fruitful; the oxidation of aldehyde (*rac*)-**18** with TEMPO [21] resulted in a lower yield (28%). The carboxylic acid (*rac*)-**19** was then esterified with ʟ-(−)-menthol (**8**) for a chiral resolution. The reaction was mediated smoothly by MNBA [9] in 85% yield to give a diastereomeric mixture of menthyl esters 20* and **20** after silica gel column chromatography. As shown in Figure 6, the clean separation of 20* and **20** was realized by preparative HPLC with a CHIRALPAK IC column to furnish 20* (*t*_R_ 9.6 min) and **20** (*t*_R_ 11.8 min) in a 20*/**20** ratio of 53:46, for which the stereochemical configuration was computationally and spectroscopically determined as 2*S* and 2*R*, respectively, one step later for 21* and **21** (see below).

**Scheme 6:**
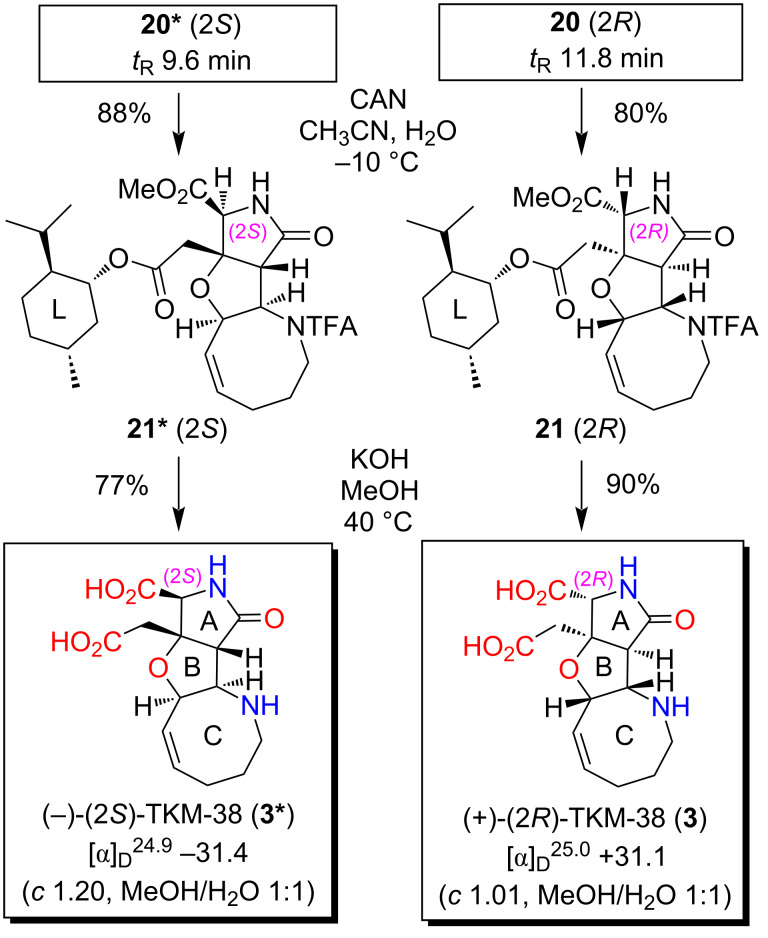
Final elaboration towards (2*R*)- and (2*S*)-TKM-38.

**Figure 7:**
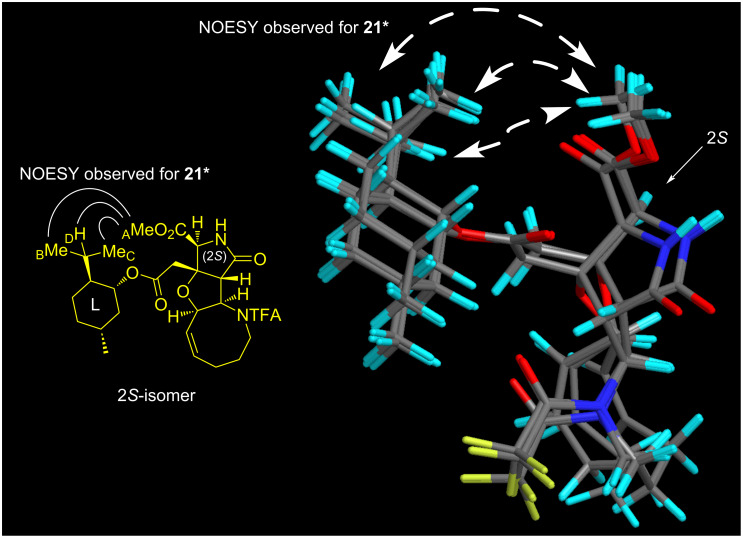
Superimposed structures of the top 5 stable conformers (89.9% total population) generated by CONFLEX (MMFF94S) for the (2*S*)-isomer, which is consistent with the NOESY crosspeaks observed for 21* (400 MHz, CDCl_3_). See Supporting Information File 1 for the stereo diagram.

**Figure 8:**
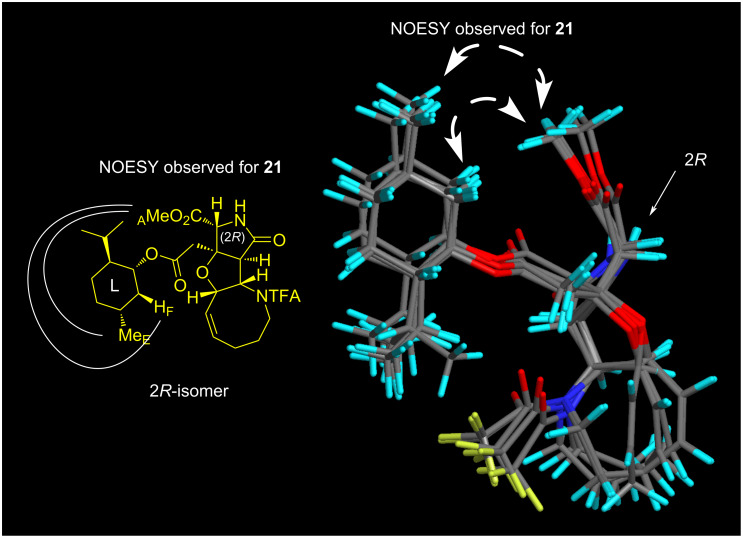
Superimposed structures of the top 5 stable conformers (76.8% total population) generated by CONFLEX (MMFF94S) for the (2*R*)-isomer, which is consistent with the NOESY crosspeaks observed for **21** (400 MHz, CDCl_3_). See Supporting Information File 1 for the stereo diagram.

The PMB group of 20* and **20** was then independently removed by CAN at −10 °C to give 21* and **21** in 88% and 80% yield, respectively (Scheme 6). With 21* and **21**, the stereochemistry was determined on the basis of the NOESY data in combination with the conformational analyses by CONFLEX, as follows. Thus, as for 21* (see Figure 7), three characteristic NOESY crosspeaks observed between isopropyl protons and methyl ester protons (Me_A_/Me_B_, Me_A_/Me_C_, Me_A_/H_D_) were found to be reasonably accounted for by the top 5 stable conformers (89.9% total population) calculated for the (2*S*)-isomer (MMFF94S). On the other hand, two NOESY crosspeaks were observed for Me_A_/Me_E_ and Me_A_/H_F_ for **21** (see Figure 8), which were consistent with the top 5 stable conformers for the (2*R*)-isomer (76.8% total population, MMFF94S). It was thus concluded that 20* and 21* are isomers with a (2*S*)-configuration, and the stereochemistry of **20** and **21** is 2*R*, as described in Figure 6, Scheme 5, and Scheme 6. The assignments were undoubtedly verified by the PGME amide analysis [22–23] (see the Supporting Information File 2).

**Figure 9:**
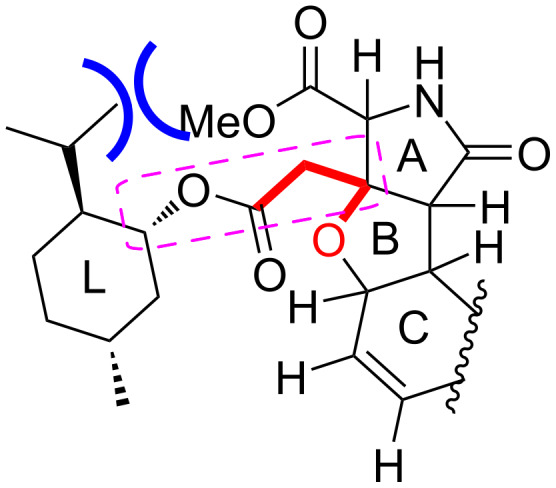
Key interactions that are supposed to control the spatial arrangement of the heterotricycle and the menthyl rings in **10**, 10*, **21**, and 21*.

The stereochemical analyses carried out here and in our previous study [4] have been supported by taking into account the steric interactions between the heterotricycle and the menthyl ring, as follows. There are no major differences in the conformations of the heterotricycle and the menthyl rings in **10**, 10*, **21**, and 21*. It is reasonably speculated that 1) the four bonds between the rings, surrounded by a purple square (Figure 9), are preferably in a linear zigzag arrangement, 2) the dihedral angle of O–C–CH_2_–C, shown in red color, is preferably 180°, and 3) on the other hand, a steric repulsion, shown in blue color, seems to occur between the isopropyl group and the methyl ester. For the (*R*)-isomers **10** and **21**, these three conditions match to define the stable conformer (see Figure 3 and Figure 8). However, the situation is different for the (*S*)-isomers 10* and 21* because there is no conformation that simultaneously fulfills these three conditions in these compounds. Thus, in 21*, to avoid a steric repulsion with the large ring C with the TFA group, the isopropyl group takes a conformation proximal to the methyl ester (see Figure 7). For 10*, with smaller size of the ring C, a conformer in which the isopropyl group and the methyl ester are separated also seems to be energetically advantageous. We speculate that, because of the small energy difference between the possible multiple conformers, the four bonds in a purple square would be freely rotating in 10* (see Supporting Information File 1 for the conformers). The correctness of the configurational analysis has been proven by single-crystal X-ray analysis of the (2*R*)-MC-27 precursor **10** (2*R*, Figure 4), and the analysis based on the PGME amide method [22–23] applied to the TKM-38 precursor (*rac*)-**19** (see Supporting Information File 2).

Finally, the hydrolytic removal of the menthyl, methyl, and TFA groups was attempted towards both enantiomers of TKM-38, **3** and 3* (Scheme 6). A preliminary study with 21* (2*S*) showed the low reactivity under acidic conditions (6 M aq HCl, MeOH, 65 °C) [5,24], which resulted in the quantitative recovery of the substrate 21* (2*S*). We then examined an alkaline hydrolysis (KOH, MeOH, H_2_O, 40 °C) [25–26], which gratifyingly furnished (2*S*)-TKM-38 (3*) in a good yield (77%) after ion-exchange chromatography (Dowex^®^ 50W x8-200, H^+^ form). LiOH, which was used for the final deprotection in the synthesis of the MC-27 enantiomers **4** and 4* (see Scheme 3 and Scheme 4), was not capable of facilitating the removal of the TFA group of 21* (2*S*). The procedure with KOH also provided the (2*R*)-enantiomer **3** of TKM-38 from **21** (2*R*) in 90% yield.

### Neuronal activity

The behavioral activity of mice was evaluated with the artificial glutamate analogs synthesized in this study. An intracerebroventricular injection (50 μg/mouse) of (2*R*)-MC-27 (**4**) caused a weak inhibition of the voluntary action of the mouse, which had been observed previously with (*rac*)-MC-27 with a nearly identical potency [5]. Therefore, it was concluded that the (2*R*)-enantiomer is responsible for the neuroactivity of (*rac*)-MC-27.

On the other hand, neither of the enantiomers of the new analog TKM-38, **3** and 3* bearing an eight-membered azacycle, showed a behavioral activity; no effects were observed on the voluntary action of mice upon intracerebroventricular injection (50 μg/mouse).

## Conclusion

We showed here in detail that the ester formation of the carboxylic acid intermediates (*rac*)-**7** and (*rac*)-**19** with ʟ-(−)-menthol (**8**) [4] enables the chiral resolution of the heterotricyclic artificial glutamate analogs more practically than our previous method using a chiral amine as the starting material [3]. In the present study, the correctness of the configurational analysis of **10** (2*R*) as well as 21* and **21** based on NOESY data and conformational calculations was undoubtedly proven by the crystallographic data (see Figure 4) and the PGME method (see Supporting Information File 2), respectively, justifying the propriety of the analyses in a series of studies [4] employing menthol-mediated chiral resolution as well. It should be noted, however, that menthyl esterification is not generally applicable to the configurational analysis of chiral carboxylic acid, from the fact that no other examples have been reported so far. In this study, the bulkiness and the rigidity of the heterotricyclic skeleton of menthyl esters **10** (2*R*) and 21*/**21** would have enabled configurational analysis based on NOESY data and conformational calculations.

The mice in vivo assay in the present study showed that, as for MC-27 (**4**), the (2*R*)-isomer is the neuroactive enantiomer. It is again interesting that the (2*R*)-isomer is neuronally active because the (2*S*)-isomer is generally neuroactive for glutamic acid and some natural products with a glutamate motif, dysiherbaine [27] and kainic acid [28]. Since the (2*R*)-isomer is the neuronally active enantiomer in these analogs (Figure 1), our future studies will straightforwardly focus on the enantioselective synthesis of only the (2*R*)-isomer. The asymmetric Ugi reaction recently developed [29] is of interest for the selective preparation of (2*R*)-**6** (Scheme 1 and Scheme 5) [6,8]. Studies are in progress to develop an asymmetric Ugi/Diels–Alder reaction, and the results will be reported in due course.

**Figure 10:**
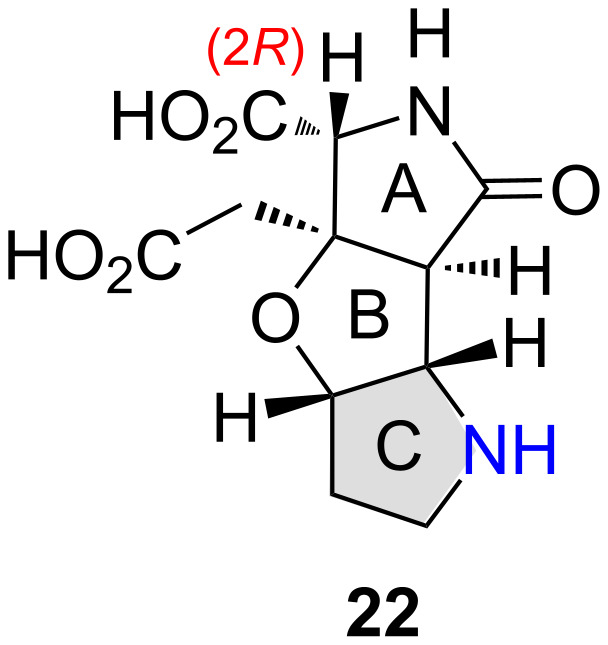
The future synthetic target **22** is expected to show potent neuroactivity.

The in vivo inactivity of TKM-38 (**3**) found in this study shows the less potent neuroactivity of analogs with a larger ring C (see analogs **1**–**3** in Figure 1). The neuroactivity of the new analog **22**, with smaller five-membered azacycle as the ring C (Figure 10), is therefore, of interest [5], and the synthesis is also underway in our laboratory.

## Experimental

Procedures for all chemical syntheses are described in Supporting Information File 3.

### Molecular modeling

The CONFLEX calculations were performed using BARISTA software (Version 1.2.2.22, Conflex Corporation), employing initial conformations generated by MM2 (ChemBio3D Ultra, Version 14.0.0.117). The calculations were basically independent from the initial conformations. A same set of conformers was obtained after CONFLEX calculations, starting with different conformers employed as initial conformers.

### Mice in vivo behavioral assay

The mice in vivo assay was performed under approval by the Ethical Committee of Experimental Animal Care at Hokkaido University. All experiments were performed in compliance with the relevant laws and institutional guidelines.

An aqueous solution (20 μL) of the sample was injected intracerebroventricularly in male ddY mice of 3 to 4 weeks (Japan SLC Inc, Hamamatsu) as described previously [30]. The effects on the behavior of mice were evaluated according to our reported procedures [3].

## Supporting Information

File 1 Synthetic procedures.

File 2 NMR spectra of all new compounds.

File 3 X-ray structure of the menthyl ester **10**.

File 4 CIF file for the X-ray structure of the menthyl ester **10**.

File 5 Stereo diagrams for **10**, 21*, and **21** as well as superimposed structures of the stable conformers of 10*.

File 6 Assignments and intensities of all NOESY crosspeaks observed for 10*, **10**, 21*, and **21**.

File 7 Stereochemical analysis of TKM-38 by the PGME amide method, as a support for the original determination of the configuration of menthyl esters **21** and 21* based on NOESY spectra and CONFLEX calculations.
