# Supplementary material for: Menthyl esterification allows chiral resolution for the synthesis of artificial glutamate analogs
Source: Beilstein J Org Chem. 2021 Feb 24;17:540–50. doi: 10.3762/bjoc.17.48 (PMC7934734; doi:10.3762/bjoc.17.48)
Supplement: File 1 — Synthetic procedures. [file Beilstein_J_Org_Chem-17-540-s001.pdf]

BEILSTEIN JOURNAL OF ORGANIC CHEMISTRY

# Supporting Information

for

# Menthyl esterification allows chiral resolution for the synthesis of artificial glutamate analogs

Kenji Morokuma, Shuntaro Tsukamoto, Kyosuke Mori, Kei Miyako, Ryuichi Sakai, Raku Irie and Masato Oikawa

*Beilstein J. Org. Chem.* **2021,** *17,* 540–550. doi:10.3762/bjoc.17.48

# Synthetic procedures

License and Terms: This is a supporting information file under the terms of the Creative Commons Attribution License (https://creativecommons.org/licenses/by/4.0). Please note that the reuse, redistribution and reproduction in particular requires that the author(s) and source are credited and that individual graphics may be subject to special legal provisions.
The license is subject to the *Beilstein Journal of Organic Chemistry* terms and conditions: (https://www.beilstein-journals.org/bjoc/terms)

**Contents:**

Synthetic procedures **S2**
References **S21**

**Synthetic procedures**

**General methods.** All reactions sensitive to air or moisture were carried out in oven-dried glassware under argon atmosphere unless otherwise noted. $CH_2Cl_2$, $Et_2O$, and THF were purified by a Glass Contour Solvent Dispensing System (Nikko Hansen). All other reagents were purchased at the highest commercial grade and used directly, unless otherwise stated.

Analytical thin-layer chromatography (TLC) was performed using Merck silica gel 60 $F_{254}$ plates (0.25 mm thickness). Flash column chromatography was carried out using Fuji Silysia silica gel BW-300 (200-400 mesh), Kanto Chemical silica gel 60 N (40-50 μm), or Yamazen silica gel CHIRALFLASH IC or HiFlash (SiOH-30μ Premium, 30 μm, 60 Å) with automated flash column systems EPCLC-Wprep2XY-10VW (Yamazen Corporation). Reversed-phase silica gel column chromatography was carried out using Fuji Silysia Chromatorex DM1020T (ODS, 100-200 mesh). For high-performance liquid chromatography (HPLC), JASCO LC-2000Plus series was used.

The specific rotation ($[\alpha]_D$) was recorded on a JASCO P-1030 polarimeter. IR spectra were recorded on a JASCO FT/IR-400 spectrometer.

$^1$H and $^{13}$C NMR spectra were recorded on a BRUKER AVANCE 400 spectrometer or a BRUKER AVANCE III HD 400 spectrometer. Chemical shift values are reported in δ (ppm) with reference to internal residual solvent ($^1$H NMR, $CHCl_3/CDCl_3$ (7.24), $C_6HD_5/C_6D_6$ (7.15), $HDO/D_2O$ (4.70), $CHD_2OD/CD_3OD$ (3.30); $^{13}$C NMR, $CDCl_3$ (77.0), $C_6D_6$ (128.0), $D_2O$ (-), $CD_3OD$ (49.0)). Coupling constants (*J*) are reported in Hertz (Hz). The following abbreviations are used to designate the multiplicities; s = singlet, d = doublet, dd = double doublet, ddd = double double doublet, dddd = double double double doublet, t = triplet, q = quartet, m = multiplet, br = broad.

ESI mass spectra were measured with a Q Exactive Focus Hybrid Quadrupole-Orbitrap mass spectrometer (Thermo Fisher Scientific, San Jose, CA).

**Methyl (4a*R*,5a*S*,6*S*,8a*R*,8b*R*)-5a-(2-(((1*R*,2*S*,5*R*)-2-isopropyl-5-methylcyclohexyl)oxy)-2-oxoethyl)-7-(4-methoxybenzyl)-8-oxo-2,4a,5a,6,7,8,8a,8b-octahydropyrano[2',3':4,5]furo[2,3-*c*]pyrrole-6-carboxylate (9*) and methyl (4a*S*,5a*R*,6*R*,8a*S*,8b*S*)-5a-(2-(((1*R*,2*S*,5*R*)-2-isopropyl-5-methylcyclohexyl)oxy)-2-oxoethyl)-7-(4-methoxybenzyl)-8-oxo-2,4a,5a,6,7,8,8a,8b-octahydropyrano[2',3':4,5]furo[2,3-*c*]pyrrole-6-carboxylate (9)**

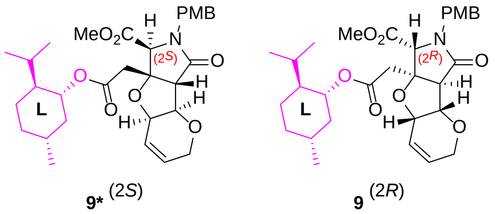


**9*** (2*S*) retention time: 7.0 min    **9** (2*R*) retention time: 11.5 min

To a stirred solution of the racemic carboxylic acid (*rac*)-**7** [1] (1.40 g, 3.35 mmol), L-(–)-menthol (**8**, 629 mg, 4.03 mmol), and 2-methyl-6-nitrobenzoic anhydride (1.27 g, 3.69 mmol) in $CH_2Cl_2$ (28.0 mL) at rt were added $Et_3N$ (1.40 mL, 10.0 mmol) and DMAP (93.0 mg, 0.761 mmol). After stirring for 13 h, to the reaction mixture was added saturated aqueous $NaHCO_3$ (20 mL), and the mixture was extracted with $CH_2Cl_2$ (2×10 mL). The combined organic layer was concentrated under reduced pressure. The residue was purified by column chromatography on silica gel (SiOH-30μ Premium, 45 g, EtOAc/hexane 33:67) to give an inseparable mixture of the L-menthyl ester **9*** (2*S*) and **9** (2*R*) (**9***/**9** 50.5:49.5, 1.70 g, 91.3%) as a colorless oil. The mixture was further purified by column chromatography on silica gel (CHIRALFLASH IC, 30 g, EtOH/hexane 65:35) to give the L-menthyl ester **9*** (2*S*, less polar, 843 mg, 45.3%) as a colorless amorphous solid, and the L-menthyl ester **9** (2*R*, more polar, 825 mg, 44.4%) as a colorless powder. The stereochemical configuration was elucidated later at the stage of **10*** and **10**.

Data for the L-menthyl ester **9*** (2*S*): $t_R$ 8.5 min (4.6×150 mm CHIRALFLASH IC column, EtOH/hexane 65:35, 20 mL/min, 25 °C); $[\alpha]^{24}_D$ -63.7 (*c* 0.765, $CHCl_3$); IR (ATR) 2947, 2913, 1738, 1698, 1513, 1249, 1201, 1177, 1086, 1049, 1033, 995, 844, 821, 685, 592 $cm^{-1}$; $^1H$ NMR (400 MHz, $CDCl_3$) δ 7.11 (d, *J* = 8.7 Hz, 2H), 6.81 (d, *J* = 8.7 Hz,

2H), 5.99 (dd, *J* = 10.3, 4.1 Hz, 1H), 5.90 (m, 1H), 4.71 (d, *J* = 14.9 Hz, 1H), 4.60 (ddd, *J* = 10.9, 10.9, 4.4 Hz, 1H), 4.41 (d, *J* = 3.1 Hz, 1H), 4.36 (s, 1H), 4.15 (m, 1H), 4.09 (d, *J* = 14.9 Hz, 1H), 4.05-3.96 (m, 2H), 3.77 (s, 3H), 3.55 (s, 3H), 3.32 (s, 1H), 3.09 (d, *J* = 16.4 Hz, 1H), 2.70 (d, *J* = 16.4 Hz, 1H), 1.90 (m, 1H), 1.81 (m, 1H), 1.68-1.57 (m, 2H), 1.40 (m, 1H), 1.28 (m, 1H), 0.99 (m, 1H), 0.92-0.75 (m, 2H), 0.85 (d, *J* = 6.7 Hz, 3H), 0.82 (d, *J* = 6.7 Hz, 3H), 0.69 (d, *J* = 6.7 Hz, 3H); $^{13}$C NMR (100 MHz, $CDCl_3$) δ 171.0, 170.4, 169.6, 159.2, 130.1, 129.8 (× 2), 127.3, 122.9, 114.1 (× 2), 85.4, 78.4, 74.6, 73.5, 68.3, 64.0, 57.8, 55.3, 52.4, 46.9, 45.5, 40.7, 40.4, 34.2, 31.3, 26.1, 23.5, 22.0, 20.7, 16.5; HRMS (ESI, positive) calcd for $C_{31}H_{42}NO_8^+$ [$(M+H)^+$] 556.29049, found 556.29034.

Data for the L-menthyl ester **9** (2*R*): $t_R$ 12.6 min (4.6×150 mm CHIRALFLASH IC column, EtOH/hexane 65:35, 20 mL/min, 25 °C); $[\alpha]^{24}_D$ -14.3 (*c* 0.600, $CHCl_3$); IR (ATR) 1739, 1720, 1677, 1514, 1248, 1209, 1180, 1088, 1037, 1001, 984, 965, 813, 692 cm$^{-1}$; $^1$H NMR (400 MHz, $CDCl_3$) δ 7.10 (d, *J* = 8.7 Hz, 2H), 6.81 (d, *J* = 8.7 Hz, 2H), 6.00 (dd, *J* = 10.3, 3.6 Hz, 1H), 5.91 (ddd, *J* = 10.3, 2.1, 2.0 Hz, 1H), 4.79 (d, *J* = 14.9 Hz, 1H), 4.61 (ddd, *J* = 10.9, 10.9, 4.4 Hz, 1H), 4.41-4.38 (m, 2H), 4.40 (s, 1H), 4.14 (dd, *J* = 17.0, 4.1 Hz, 1H), 4.02 (m, 1H), 4.01 (d, *J* = 14.9 Hz, 1H), 3.77 (s, 3H), 3.56 (s, 3H), 3.31 (s, 1H), 3.11 (d, *J* = 17.2 Hz, 1H), 2.75 (d, *J* = 17.2 Hz, 1H), 1.94 (m, 1H), 1.78 (m, 1H), 1.68-1.57 (m, 2H), 1.40 (m, 1H), 1.28 (m, 1H), 1.06-0.75 (m, 3H), 0.85 (d, *J* = 6.7 Hz, 3H), 0.80 (d, *J* = 6.7 Hz, 3H), 0.68 (d, *J* = 6.7 Hz, 3H); $^{13}$C NMR (100 MHz, $CDCl_3$) δ 170.9, 170.4, 169.7, 159.2, 130.3, 129.7 (× 2), 127.3, 122.8, 114.1 (× 2), 85.2, 78.4, 74.7, 73.6, 68.3, 64.0, 57.9, 55.3, 52.3, 46.9, 45.4, 40.8, 40.5, 34.2, 31.4, 26.1, 23.4, 22.0, 20.7, 16.3; HRMS (ESI, positive) calcd for $C_{31}H_{42}NO_8^+$ [$(M+H)^+$] 556.29049, found 556.28986.

**Methyl (4a*S*,5a*R*,6*R*,8a*S*,8b*S*)-5a-(2-(((1*R*,2*S*,5*R*)-2-isopropyl-5-methylcyclohexyl)oxy)-2-oxoethyl)-8-oxo-2,4a,5a,6,7,8,8a,8b-octahydropyrano[2',3':4,5]furo[2,3-*c*]pyrrole-6-carboxylate (10)**

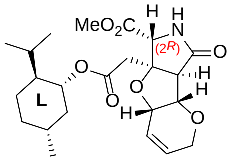

**10** (2*R*, X-ray)

To an ice-cooled solution of the *N*-PMB amide **9** (2*R*, 820 mg, 1.48 mmol) in MeCN (13 mL) and water (7 mL) was added ceric ammonium nitrate (1.63 g, 2.97 mmol). After stirring at rt for 17 h, the mixture was diluted with EtOAc (30 mL) and washed with water (30 mL) and brine (10 mL). The aqueous layer was extracted with EtOAc (10 mL), and the combined organic layer was concentrated under reduced pressure. The residue was purified by column chromatography on silica gel (SiOH-30µ Premium, 34 g, EtOAc/hexane 75:25) to give **10** (2*R*, 515 mg, 80%) as colorless crystals: Mp 183-184 °C; $[\alpha]^{24}_{D}$ -17.2 (*c* 0.345, $CHCl_3$); IR (ATR) 3216, 2925, 2870, 1759, 1736, 1703, 1669, 1204, 1193, 1179, 1085, 1040, 1031 $cm^{-1}$; $^1H$ NMR (400 MHz, $CDCl_3$) δ 6.15 (s, 1H), 6.03 (m, 1H), 5.95 (m, 1H), 4.64 (ddd, *J* = 10.9, 10.9, 4.4 Hz, 1H), 4.47 (d, *J* = 0.9 Hz, 1H), 4.29 (d, *J* = 3.1 Hz, 1H), 4.19-4.11 (m, 2H), 4.02 (m, 1H), 3.71 (s, 3H), 3.24 (s, 1H), 3.20 (d, *J* = 17.3 Hz, 1H), 2.87 (d, *J* = 17.3 Hz, 1H), 1.95 (m, 1H), 1.83 (m, 1H), 1.68-1.59 (m, 2H), 1.42 (m, 1H), 1.33 (m, 1H), 1.07-0.76 (m, 3H), 0.87 (d, *J* = 6.8 Hz, 3H), 0.85 (d, *J* = 7.1 Hz, 3H), 0.71 (d, *J* = 6.9 Hz, 3H); $^{13}C$ NMR (100 MHz, $CDCl_3$) δ 173.5, 170.4, 170.0, 130.9, 122.3, 87.5, 78.2, 74.8, 73.3, 64.4, 64.1, 56.9, 52.6, 46.9, 40.8, 40.7, 34.2, 31.4, 26.1, 23.4, 22.0, 20.7, 16.3; HRMS (ESI, positive) calcd for $C_{23}H_{34}NO_7^+$ $[(M+H)^+]$ 436.23298, found 436.23285.

The absolute configuration was determined to be 2*R* by the X-ray crystallographic analysis. See the other Supporting Information Files for details.

**(4a*S*,5a*R*,6*R*,8a*S*,8b*S*)-5a-(Carboxymethyl)-8-oxo-2,4a,5a,6,7,8,8a,8b-octahydropyrano[2',3':4,5]furo[2,3-*c*]pyrrole-6-carboxylic acid ((2*R*)-MC-27, 4)**

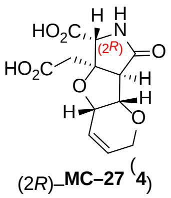


(2*R*)-**MC–27** (**4**)

A suspension of the diester **10** (2*R*, 102.0 mg, 0.234 mmol) in hydrochloric acid (6 M, 2.0 mL) and 1,4-dioxane (1.0 mL) was heated to 75 °C for 4 days. The mixture was then concentrated under reduced pressure. The residual solid was triturated with $Et_2O$ (8 mL) to give crude (2*R*)-MC-27 (**4**, 61.0 mg) as a white solid. The solid was purified by crystallization from water (5 mL) to give (2*R*)-MC-27 (**4**, 31.7 mg, 0.112 mmol, 48%) as a colorless powder: $[\alpha]^{24}_{D}$ +20.1 (*c* 0.107, MeOH); IR (ATR) 3373, 1719, 1705, 1664, 1437, 1418, 1404, 1258, 1223, 1200, 1183, 1112, 1094, 1076, 1059, 1038, 986, 675, 623 $cm^{-1}$; $^{1}H$ NMR (400 MHz, $D_2O$) δ 6.13 (dd, *J* = 10.3, 2.6 Hz, 1H), 5.93 (m, 1H), 4.42 (s, 1H), 4.28-4.06 (m, 4H), 3.31 (s, 1H), 3.10 (d, *J* = 17.0 Hz, 1H), 2.86 (d, *J* = 17.0 Hz, 1H); $^{13}C$ NMR (100 MHz, $D_2O$) δ 175.8, 173.5, 173.3, 132.1, 120.3, 87.0, 77.6, 73.1, 66.0, 64.2, 57.3, 40.3; HRMS (ESI, positive) calcd for $C_{12}H_{13}NO_7Na^+$ $[(M+Na)^+]$ 306.05842, found 306.05855.

**Methyl (4a*R*,5a*S*,6*S*,8a*R*,8b*R*)-5a-(2-(((1*R*,2*S*,5*R*)-2-isopropyl-5-methylcyclohexyl)oxy)-2-oxoethyl)-8-oxo-2,4a,5a,6,7,8,8a,8b-octahydropyrano[2',3':4,5]furo[2,3-c]pyrrole-6-carboxylate (10*)**

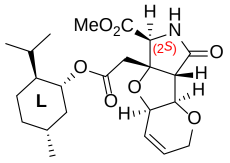


**10*** (2*S*)

To an ice-cooled solution of the *N*-PMB amide **9*** (2*S*, 294 mg, 0.529 mmol) in MeCN (5 mL) and water (2.5 mL) was added ceric ammonium nitrate (577 mg, 1.05 mmol). After stirring at rt for 32 h, the mixture was diluted with EtOAc (20 mL) and water (10 mL). The organic layer was separated and filtered through a pad of silica gel (Chromatorex NH-DM2035, Fuji Silysia Chemical Ltd, 5 g, EtOAc/hexane 60:40), and the filtrate was concentrated under reduced pressure. The residual solid was triturated with $iPr_2O$ (4 mL) to give **10*** (2*S*, 153 mg, 66%) as a colorless powder: $[\alpha]^{20}_D$ -76.6 (*c* 0.610, $CHCl_3$); IR (ATR) 3199, 3114, 2958, 2944, 2917, 2864, 2848, 1737, 1712, 1344, 1200, 1179, 1131, 1085, 1056, 1049, 995, 984, 706 $cm^{-1}$; $^1H$ NMR (400 MHz, $CDCl_3$) δ 6.38 (s, 1H), 6.02 (m, 1H), 5.94 (m, 1H), 4.63 (ddd, *J* = 10.9, 10.9, 4.4 Hz, 1H), 4.44 (d, *J* = 0.9 Hz, 1H), 4.30 (d, *J* = 3.1 Hz, 1H), 4.19-4.10 (m, 2H), 4.02 (m, 1H), 3.72 (s, 3H), 3.24 (s, 1H), 3.19 (d, *J* = 16.6 Hz, 1H), 2.87 (d, *J* = 16.6 Hz, 1H), 1.93 (m, 1H), 1.85 (m, 1H), 1.68-1.59 (m, 2H), 1.42 (m, 1H), 1.32 (m, 1H), 1.07-0.76 (m, 3H), 0.87 (d, *J* = 6.7 Hz, 3H), 0.85 (d, *J* = 7.1 Hz, 3H), 0.72 (d, *J* = 6.9 Hz, 3H); $^{13}C$ NMR (100 MHz, $CDCl_3$) δ 173.7, 170.4, 169.9, 130.6, 122.3, 87.6, 78.3, 74.7, 73.2, 64.4, 64.1, 56.9, 52.6, 46.8, 40.7, 40.6, 34.2, 31.3, 26.0, 23.4, 22.0, 20.7, 16.4; HRMS (ESI, positive) calcd for $C_{23}H_{33}NO_7Na^+$ [$(M+Na)^+$] 458.2149, found 458.2139.

**(4a*R*,5a*S*,6*S*,8a*R*,8b*R*)-5a-(Carboxymethyl)-8-oxo-2,4a,5a,6,7,8,8a,8b-octahydropyrano[2',3':4,5]furo[2,3-*c*]pyrrole-6-carboxylic acid ((2*S*)-MC-27, 4*)**

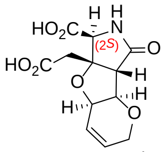


(–)-(2*S*)-**MC-27** (**4***)

A stirred mixture of the diester **10*** (2*S*, 114 mg, 0.262 mmol) in hydrochloric acid (6 M, 2 mL) and 1,4-dioxane (1 mL) was heated to 85 °C for 70 h. The mixture was then concentrated under reduced pressure. The residual solid was triturated with $Et_2O$ (5 mL) to give a pale brown solid (66.5 mg), which was crystallized from water (6 mL) to give (2*S*)-MC-27 (**4***, 46.3 mg, 0.163 mmol, 62%) as a pale brown powder: $[\alpha]^{24}_{D}$ -20.9 (*c* 0.149, MeOH).

The other spectroscopic data were in good agreement with those for **4** (see above).

**2,2,2-Trifluoro-*N*-(pent-4-en-1-yl)acetamide (12)**

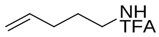

**12**

To a stirred solution of trifluoroacetamide (6.00 g, 53.1 mmol) in DMF (120 mL) at 0 °C was added sodium hydride (55% dispersion in paraffin liquid, 2.54 g, 58.4 mmol). After stirring at rt for 2 h, to the mixture was added 5-bromo-1-pentene (6.92 mL, 58.4 mmol). After stirring at 50 °C for 21 h, the mixture was cooled to rt, poured into water (100 mL) and extracted with $Et_2O$ (3×100 mL). The combined extracts were washed with brine (3×100 mL), dried over $Na_2SO_4$, and concentrated under reduced pressure. The residue was purified by column chromatography on silica gel (SiOH-30μ Premium, 40 g, EtOAc/hexane 1:9) to give the amide **12** (6.07 g, 63%) as a colorless oil: IR (ATR) 3305, 3102, 2941, 1703, 1560, 1444, 1347, 1209, 1183, 994, 917 $cm^{-1}$; $^1$H NMR (400 MHz, $CDCl_3$) δ 7.00 (m, 1H), 5.74 (m, 1H), 4.99 (m, 2H), 3.32 (dt, *J* = 7.3, 7.3 Hz, 2H), 2.07 (dt, *J* = 7.3, 7.3 Hz, 2H), 1.65 (tt, *J* = 7.3, 7.3 Hz, 2H); $^{13}$C NMR (100 MHz, $CDCl_3$) δ 157.4 (q, *J* = 36.7 Hz), 137.0, 117.3 (q, *J* = 287.6 Hz), 115.6, 39.5, 30.7, 27.8; HRMS (ESI, positive) calcd for $C_7H_{10}F_3NONa^+$ [$(M+Na)^+$] 204.0607, found 204.0607.

**(3*R**,3a*R**,6*R**,7*S**,7a*S**)-*N*-Benzyl-2-(4-methoxybenzyl)-1-oxo-7-(2,2,2-trifluoro-*N*-(pent-4-en-1-yl)acetamido)-1,2,3,6,7,7a-hexahydro-3a,6-epoxyisoindole-3-carboxamide ((*rac*)-13)**

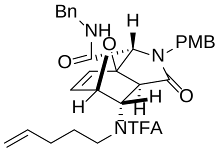


*rac*–**13**

To a stirred solution of the amide **12** (34.2 mg, 0.189 mmol) in DMF (0.47 mL) at rt were added the iodide (*rac*)-**6** (50.0 mg, 0.0943 mmol) and cesium carbonate (61.4 mg, 0.189 mmol). After 13 h, the mixture was poured into saturated aqueous $NH_4Cl$ (2 mL) and extracted with $Et_2O$ (3×2 mL). The combined extracts were washed with brine (3×2 mL), dried over $Na_2SO_4$, and concentrated under reduced pressure. The residue was purified by column chromatography on silica gel (SiOH-30μ Premium, 7 g, EtOAc/hexane 4:6) to give the amide (*rac*)-**13** (28.0 mg, 51%) as a colorless oil: IR (ATR) 3315, 2935, 1686, 1513, 1350, 1246, 1191, 1144, 1032, 1144, 700 $cm^{-1}$; $^1$H NMR (400 MHz, $CDCl_3$) δ 7.38-7.30 (m, 3H), 7.21 (d, *J* = 7.2 Hz, 2H), 7.05 (d, *J* = 8.3 Hz, 2H), 6.79 (d, *J* = 8.3 Hz, 2H), 6.35 (d, *J* = 6.0 Hz, 1H), 6.23 (d, *J* = 6.0 Hz, 1H), 6.02 (s, 1H), 5.74 (m, 1H), 5.66 (d, *J* = 4.0 Hz, 1H), 5.01 (dd, *J* =13.4, 13.4 Hz, 2H), 4.90 (d, *J* = 14.9 Hz, 1H), 4.43-4.34 (m, 3H), 3.97 (d, *J* = 14.9 Hz, 1H), 3.95 (s, 1H), 3.75 (s, 3H), 3.53 (m, 1H), 3.05 (m, 1H), 2.88 (d, *J* = 3.7 Hz, 1H), 2.15-1.96 (m, 2H), 1.66-1.49 (m, 2H); $^{13}$C NMR (100 MHz, $CDCl_3$) δ 172.5, 166.9, 159.4, 157.6 (q, *J* = 35.7 Hz), 137.1, 136.6, 135.4, 134.2, 129.6 (× 2), 128.9 (× 2), 128.1, 127.9 (× 2), 126.6, 119.1 (q, *J* = 287.1 Hz), 116.0, 114.3 (× 2), 90.8, 80.7, 62.8, 59.1, 55.2, 51.7, 47.1, 45.5, 44.0, 30.6, 27.5; HRMS (ESI, positive) calcd for $C_{31}H_{32}F_3N_3O_5Na^+$ [$(M+Na)^+$] 606.2186, found 606.2187.

**(*E*)- and (*Z*)-2-((6a*S**,7a*R**,8*R**,10a*S**,10b*S**,*Z*)-8-(Benzylcarbamoyl)-9-(4-methoxybenzyl)-10-oxo-1-(2,2,2-trifluoroacetyl)-1,2,3,4,6a,8,9,10,10a,10b-decahydro-7a*H*-pyrrolo[3',4':4,5]furo[3,2-*b*]azocin-7a-yl)vinyl acetate ((*rac*)-16)**

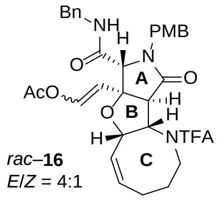


To a stirred solution of the diene (*rac*)-**13** (5.17 mg, 0.0089 mmol) in benzene (1.26 mL) at 69 °C were added vinyl acetate (0.00409 mL, 0.0443 mmol) and Zhan catalyst-1B (**14**, 0.20 mg, 0.0003 mmol). After 25 h, the mixture was concentrated under reduced pressure. The ruthenium catalyst was removed by passing through a short pad of silica gel (60 N, 600 mg, EtOAc/hexane 3:7). The filtrate was concentrated under reduced pressure to give a residue (5.85 mg) that was a mixture mainly composed of the triene (*rac*)-**15**.

The residue thus obtained, without purification, was dissolved in benzene (1.26 mL). To the stirred mixture at 69 °C was added Zhan catalyst-1B (0.20 mg, 0.0003 mmol). After 4 h, the mixture was cooled to rt and concentrated under reduced pressure. The residue was purified by column chromatography on silica gel (60 N, 600 mg, EtOAc/hexane 5:5) to give the heterotricycle (*rac*)-**16** (*E/Z* 4:1, 3.63 mg, 64%) as a brown oil: IR (ATR) 3293, 2939, 1761, 1690, 1514, 1439, 1246, 1205, 1178, 1145, 1032 $cm^{-1}$; $^{1}H$ NMR (selected for the *trans*-isomer, 400 MHz, $CDCl_3$) δ 7.43 and 7.40 (two doublets, *J* =12.7, 12.3 Hz each, 1H total), 7.36-7.26 (m, 3H), 7.20 (d, *J* = 8.5 Hz, 2H), 7.11-6.99 (m, 2H), 6.83-6.74 (m, 2H), 6.03 (m, 1H), 5.67 (d, *J* = 12.7 Hz, 1H), 5.63 (d, *J* = 12.3 Hz, 1H), 5.34 (m, 1H), 5.21 (d, *J* = 14.2 Hz, 1H), 4.68 (d, *J* = 5.5 Hz, 1H), 4.49-4.24 (m, 2H), 4.15 (t, *J* = 5.5 Hz, 1H), 3.96 (d, *J* = 14.2 Hz, 1H), 3.96 (m, 1H), 3.77 (s, 3H), 3.60 (s, 1H), 3.09 (s, 1H), 2.86 (m, 1H), 2.65 (m, 1H), 2.14 (s, 3H), 2.05 (m, 1H), 1.68-1.44 (m, 2H); $^{13}C$ NMR (selected for the major rotamer of the *trans*-isomer, 100 MHz, $CDCl_3$) δ 170.8, 167.7, 166.6,

159.5, 156.5 (q, *J* = 35.6 Hz), 138.8, 137.9, 137.2, 130.1, 129.9, 128.8 (× 2), 128.1 (× 2), 127.8, 126.9, 122.2, 116.6 (q, *J* = 287.5 Hz), 114.3 (× 2), 111.9, 83.2, 79.6, 70.7, 66.4, 58.3, 55.3, 46.6, 45.3, 43.8, 25.7, 25.3, 20.6; HRMS (ESI, positive) calcd for $C_{33}H_{34}F_3N_3O_7Na^+$ [$(M+Na)^+$] 664.2241, found 664.2239.

**(*E*)- and (*Z*)-2-((6a*S**,7a*R**,8*R**,10a*S**,10b*S**,*Z*)-8-(Benzyl(tert-butoxycarbonyl)carbamoyl)-9-(4-methoxybenzyl)-10-oxo-1-(2,2,2-trifluoroacetyl)-1,2,3,4,6a,8,9,10,10a,10b-decahydro-7a*H*-pyrrolo[3',4':4,5]furo[3,2-*b*]azocin-7a-yl)vinyl acetate ((*rac*)-17)**

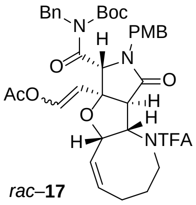

*rac*–**17**

To a stirred solution of the *N*-benzyl amide (*rac*)-**16** (337.9 mg, 0.527 mmol) in $CH_2Cl_2$ (4.7 mL) at 0 °C were added $Boc_2O$ (0.365 mL, 1.59 mmol), $Et_3N$ (0.294 mL, 2.11 mmol), and DMAP (32.16 mg, 0.263 mmol). After 17 h, the mixture was poured into saturated aqueous $NH_4Cl$ (20 mL) and extracted with EtOAc (3×20 mL). The combined extracts were washed with brine (20 mL), dried over $Na_2SO_4$, and concentrated under reduced pressure. The residue was purified by column chromatography on silica gel (SiOH-30µ Premium, 7 g, EtOAc/hexane 3:7) to give the *N*-Boc imide (*rac*)-**17** (*E/Z* 4:1, 339.4 mg, 87%) as a colorless oil: IR (ATR) 2937, 1736, 1698, 1514, 1431, 1370, 1304, 1249, 1203, 1144, 1032 $cm^{-1}$; $^1H$ NMR (selected for the *trans*-isomer, 400 MHz, $CDCl_3$) δ 7.41 and 7.38 (two doublets, *J* = 5.0, 5.0 Hz each, 1H total), 7.31-7.25 (m, 3H), 7.19 (d, *J* = 7.6 Hz, 2H), 6.96 and 6.91 (two doublets, *J* = 7.8, 7.8 Hz each, 2H total), 6.77 (d, *J* = 7.8 Hz, 2H), 5.99 (m, 1H), 5.46 (m, 1H), 5.35 (m, 1H), 4.97 (d, *J* = 14.4 Hz, 1H), 4.92 (d, *J* = 14.8 Hz, 1H), 4.79 (m, 1H), 4.64 (d, *J* = 14.8 Hz, 1H), 4.42 (m, 1H), 3.84 (m, 1H), 3.75 (s, 3H), 3.61 (d, *J* = 14.4 Hz, 1H), 3.49 (s, 1H), 3.13 (s, 1H), 2.94 (m, 1H), 2.67 (m, 1H), 2.05 and 2.02 (two singlets, 3H total), 1.95 (m, 1H), 1.81 (m, 1H), 1.57 (m, 1H), 1.28

(s, 9H); $^{13}$C NMR (selected for the major rotamer of the *trans*-isomer, 100 MHz, $CDCl_3$) δ 171.9, 171.4, 167.3, 159.4, 156.7 (q, *J* = 35.7 Hz), 151.5, 140.0, 137.2, 137.1, 130.3, 130.2, 128.3 (× 3), 128.0 (× 2), 126.3, 122.8, 116.3 (q, *J* = 288.1 Hz), 114.3, 114.1, 112.8, 83.4, 80.7, 79.8, 66.7, 59.3, 57.4, 55.2, 47.9, 46.4, 45.5, 28.2, 27.6 (× 3), 25.1, 20.5; HRMS (ESI, positive) calcd for $C_{38}H_{42}F_3N_3O_9Na^+$ [(M+Na)$^+$] 764.2765, found 764.2753.

**(6a*S**,7a*R**,8*R**,10a*S**,10b*S**,*Z*)-Methyl 9-(4-methoxybenzyl)-10-oxo-7a-(2-oxoethyl)-1-(2,2,2-trifluoroacetyl)-2,3,4,6a,7a,8,9,10,10a,10b-decahydro-1*H*-pyrrolo[3',4':4,5]furo[3,2-*b*]azocine-8-carboxylate ((*rac*)-18)**

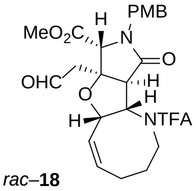


*rac*–**18**

To a stirred solution of the *N*-Boc imide (*rac*)-**17** (339.4 mg, 0.458 mmol) in MeOH (12.3 mL) at −20 °C was added $K_2CO_3$ (6.3 mg, 0.046 mmol). After 3 h, to the mixture was added $K_2CO_3$ (6.3 mg, 0.046 mmol) to complete the reaction. After 1.5 h, the mixture was poured into saturated aqueous $NH_4Cl$ (30 mL) and extracted with EtOAc (30 mL, 20 mL, and then 10 mL). The combined extracts were washed with brine (3×15 mL), dried over $Na_2SO_4$, and concentrated under reduced pressure. The residue was purified by column chromatography on silica gel (SiOH-30μ Premium, 16 g, EtOAc/hexane 4:6) to give the ester aldehyde (*rac*)-**18** (177.2 mg, 74%) as a white foam: IR (ATR) 2935, 1746, 1697, 1612, 1514, 1442, 1248, 1205, 1179, 1146, 1033 cm$^{-1}$; $^1$H NMR (400 MHz, $CDCl_3$) δ 9.69 (s, 1H), 7.04 (d, *J* = 7.8 Hz, 2H), 6.82 (d, *J* = 7.8 Hz, 2H), 5.58-5.44 (m, 2H), 5.04 (d, *J* = 14.7 Hz, 1H), 4.79 (d, *J* = 8.3 Hz, 1H), 4.19-4.00 (m, 2H), 4.05 (s, 1H), 3.85 (d, *J* = 14.7 Hz, 1H), 3.76 (s, 3H), 3.61 (s, 3H), 3.48 (s, 1H), 3.43 (d, *J* = 17.7 Hz, 1H), 3.36 (m, 1H), 2.88 (d, *J* = 17.7 Hz, 1H), 2.22 (m, 1H), 1.98-1.81 (m, 2H), 1.76 (m, 1H); $^{13}$C NMR (100 MHz, $CDCl_3$) δ 199.1, 173.1, 169.2,

159.5, 156.9 (q, *J* = 36.7 Hz), 129.7 (× 2), 128.8, 128.1, 126.7, 116.1 (q, *J* = 288.0 Hz), 114.3 (× 2), 84.9, 80.4, 73.2, 67.9, 56.1, 55.2, 52.3, 51.9, 48.7, 45.2, 27.4, 26.0; HRMS (ESI, positive) calcd for $C_{25}H_{27}F_3N_2O_7Na^+$ [$(M+Na)^+$] 547.1663, found 547.1661.

**2-((6a*S**,7a*R**,8*R**,10a*S**,10b*S**,*Z*)-9-(4-Methoxybenzyl)-8-(methoxycarbonyl)-10-oxo-1-(2,2,2-trifluoroacetyl)-2,3,4,6a,7a,8,9,10,10a,10b-decahydro-1*H*-pyrrolo[3',4':4,5]furo[3,2-*b*]azocin-7a-yl)acetic acid ((*rac*)-19)**

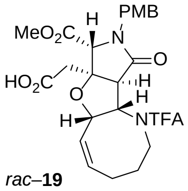

*rac*–**19**

To a stirred solution of the aldehyde (*rac*)-**18** (409.1 mg, 0.7800 mmol) in *tert*-butanol (21.0 mL) at rt was added 2-methyl-2-butene (0.412 mL, 3.893 mmol). A solution of sodium dihydrogen phosphate (102.7 mg, 0.8563 mmol) and sodium chlorite (211.6 mg, 2.340 mmol) in water (7.0 mL) was added dropwise over 10 min. After 30 min, the mixture was poured into hydrochloric acid (1 M, 30 mL) and extracted with $CH_2Cl_2$ (3×30 mL). The combined extracts were washed with brine (2×30 mL), dried over $Na_2SO_4$, and concentrated under reduced pressure. The residue was purified by trituration with $Et_2O$ three times to give the carboxylic acid (*rac*)-**19** (181.0 mg, 43%) as a white solid: IR (ATR) 2953, 1750, 1719, 1697, 1670, 1515, 1454, 1401, 1200, 1145, 1011 $cm^{-1}$; $^1H$ NMR (selected for the major rotamer, 400 MHz, $CDCl_3$) δ 7.07 (d, *J* = 7.7 Hz, 2H), 6.83 (d, *J* = 7.7 Hz, 2H), 5.60-5.47 (m, 2H), 5.04 (d, *J* = 14.6 Hz, 1H), 4.79 (d, *J* = 8.3 Hz, 1H), 4.20-4.06 (m, 2H), 4.09 (s, 1H), 3.91 (d, *J* = 14.6 Hz, 1H), 3.78 (s, 3H), 3.70 (s, 3H), 3.68 (s, 1H), 3.49-3.34 (m, 2H), 3.17 (d, *J* = 17.3 Hz, 1H), 2.29-1.68 (m, 4H); $^{13}C$ NMR (selected for the major rotamer, 100 MHz, $CD_3OD$) δ 176.0, 173.0, 170.4, 161.1, 158.3 (q, *J* = 36.1 Hz), 131.0 (×2), 130.5, 129.1, 128.2, 117.8 (q, *J* = 287.4 Hz), 115.3 (×2), 86.2, 80.9, 74.1, 69.4, 56.9, 55.7, 52.9, 52.5, 46.3, 40.4, 28.9, 26.5;

HRMS (ESI, positive) calcd for $C_{25}H_{27}F_3N_2O_8Na^+$ [$(M+Na)^+$] 563.1612, found 563.1615.

**Methyl (6a*R*,7a*S*,8*S*,10a*R*,10b*R*,*Z*)-7a-(2-(((1*R*,2*S*,5*R*)-2-isopropyl-5-methylcyclohexyl)oxy)-2-oxoethyl)-9-(4-methoxybenzyl)-10-oxo-1-(2,2,2-trifluoroacetyl)-2,3,4,6a,7a,8,9,10,10a,10b-decahydro-1*H*-pyrrolo[3',4':4,5]furo[3,2-*b*]azocine-8-carboxylate (20*) and methyl (6a*S*,7a*R*,8*R*,10a*S*,10b*S*,*Z*)-7a-(2-(((1*R*,2*S*,5*R*)-2-isopropyl-5-methylcyclohexyl)oxy)-2-oxoethyl)-9-(4-methoxybenzyl)-10-oxo-1-(2,2,2-trifluoroacetyl)-2,3,4,6a,7a,8,9,10,10a,10b-decahydro-1*H*-pyrrolo[3',4':4,5]furo[3,2-*b*]azocine-8-carboxylate (20)**

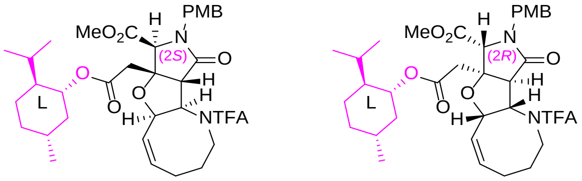


**20*** (2*S*, 53%)
retention time: 9.6 min

**20** (2*R*, 46%)
retention time: 11.8 min

To a stirred solution of the carboxylic acid (*rac*)-**19** (181.0 mg, 0.3348 mmol) in $CH_2Cl_2$ (33 mL) at rt were added L-(–)-menthol (57.6 mg, 0.368 mmol), MNBA (126.8 mg, 0.3683 mmol), $Et_3N$ (0.140 mL, 1.00 mmol), and DMAP (8.2 mg, 0.067 mmol). After 18 h, to the mixture were added L-(–)-menthol (57.6 mg, 0.368 mmol), MNBA (126.8 mg, 0.3683 mmol), $Et_3N$ (0.140 mL, 1.00 mmol), and DMAP (8.2 mg, 0.067 mmol) to complete the reaction. After 1 h, the mixture was poured into saturated aqueous $NH_4Cl$ (30 mL) and extracted with $CH_2Cl_2$ (3×30 mL). The combined extracts were dried over $Na_2SO_4$ and concentrated under reduced pressure. The residue was purified by column chromatography on silica gel (60 N, 10 g, EtOAc/hexane 3:7) to give an inseparable mixture of the menthyl ester diastereomers (dr = 6:5, 193.8 mg, 85%) as a white foam.

Purification of the mixture (dr = 6:5, 189.1 mg) by HPLC (CHIRALPACK IC, 4.6×150 mm, EtOH/hexane 0.5:9.5, 1.0 mL/min, 40 °C, detected at 254 nm) gave the iastereomerically pure menthyl esters **20*** (2*S*, 100.8

mg, $t_R$ 9.6 min) and **20** (2*R*, 87.4 mg, $t_R$ 11.8 min). The structures were determined later at the stage of **21*** and **21** after removal of the PMB group (see below).

Data for the menthyl ester **20*** (2*S*): retention time 9.6 min; $[\alpha]^{26.6}_D$ +24.8 (*c* 5.04, $CHCl_3$); IR (ATR) 2952, 2870, 1734, 1697, 1514, 1455, 1370, 1247, 1200, 1145, 1037 $cm^{-1}$; $^1H$ NMR (selected for the major rotamer, 400 MHz, $CDCl_3$) δ 7.06 (d, *J* = 8.4 Hz, 2H), 6.80 (d, *J* = 8.4 Hz, 2H), 5.55-5.45 (m, 2H), 4.98 (d, *J* = 14.7 Hz, 1H), 4.76 (d, *J* = 8.3 Hz, 1H), 4.62 (ddd, *J* = 11.0, 11.0, 4.4 Hz, 1H), 4.19 (d, *J* = 8.3 Hz, 1H), 4.07 (s, 1H), 4.01 (m, 1H), 3.98 (d, *J* = 14.7 Hz, 1H), 3.76 (s, 1H), 3.75 (s, 3H), 3.64 (s, 3H), 3.41 (m, 1H), 3.24 (d, *J* = 17.2 Hz, 1H), 3.16 (d, *J* = 17.2 Hz, 1H), 2.18 (m, 1H), 2.01-1.89 (m, 2H), 1.85 (m, 1H), 1.79 (m, 1H), 1.70 (m, 1H), 1.66-1.56 (m, 2H), 1.39 (m, 1H), 1.31 (m, 1H), 1.05-0.89 (m, 2H), 0.89-0.79 (m, 7H), 0.70 (d, *J* = 6.7 Hz, 3H); $^{13}C$ NMR (selected for the major rotamer, 100 MHz, $CDCl_3$) δ 173.7, 169.3, 169.1, 159.3, 156.8 (q, *J* = 36.3 Hz), 129.7 (× 2), 128.4, 128.3, 127.1, 116.2 (q, *J* = 288.3 Hz), 114.2 (× 2), 85.1, 79.8, 74.8, 72.1, 67.6, 55.2, 55.0, 52.3, 51.3, 46.7, 45.1, 40.3, 39.9, 34.1, 31.3, 27.3, 26.1, 26.0, 23.4, 21.9, 20.6, 16.3; HRMS (ESI, positive) calcd for $C_{35}H_{45}F_3N_2O_8Na^+$ [$(M+Na)^+$] 701.3020, found 701.3014.

Data for the menthyl ester **20** (2*R*): retention time 11.8 min; $[\alpha]^{26.7}_D$ -73.7 (*c* 4.37, $CHCl_3$); IR (ATR) 2954, 2928, 2869, 1698, 1514, 1455, 1247, 1200, 1145, 1103, 1036 $cm^{-1}$; $^1H$ NMR (selected for the major rotamer, 400 MHz, $CDCl_3$) δ 7.06 (d, *J* = 7.7 Hz, 2H), 6.80 (d, *J* = 7.7 Hz, 2H), 5.55-5.43 (m, 2H), 5.00 (d, *J* = 14.6 Hz, 1H), 4.76 (d, *J* = 8.4 Hz, 1H), 4.62 (ddd, *J* = 11.1, 11.1, 4.0 Hz, 1H), 4.17 (d, *J* = 8.4 Hz, 1H), 4.11 (s, 1H), 4.05 (m, 1H), 3.95 (d, *J* = 14.6 Hz, 1H), 3.76 (s, 1H), 3.75 (s, 3H), 3.65 (s, 3H), 3.40 (m, 1H), 3.23 (s, 2H), 2.19 (m, 1H), 2.01-1.89 (m, 2H), 1.89-1.77 (m, 2H), 1.72 (m, 1H), 1.67-1.56 (m, 2H), 1.40 (m, 1H), 1.29 (m, 1H), 0.98 (m, 1H), 0.90 (m, 1H), 0.87-0.79 (m, 7H), 0.67 (d, *J* = 6.9 Hz, 3H); $^{13}C$ NMR (selected for the major rotamer, 100 MHz, $CDCl_3$) δ 173.7, 169.6, 169.2, 159.3, 156.8 (q, *J* = 36.3 Hz), 129.7 (× 2), 128.5, 128.4, 127.1, 116.2 (q, *J* = 288.1 Hz), 114.2 (× 2), 85.2, 79.8, 74.7, 72.5, 67.8, 55.2, 55.0,

52.3, 51.4, 46.8, 45.1, 40.7, 39.6, 34.1, 31.3, 27.2, 26.1, 25.8, 23.2, 22.0, 20.7, 16.0; HRMS (ESI, positive) calcd for $C_{35}H_{45}F_3N_2O_8Na^+$ [$(M+Na)^+$] 701.3020, found 701.3014.

**Methyl (6a*R*,7a*S*,8*S*,10a*R*,10b*R*,*Z*)-7a-(2-(((1*R*,2*S*,5*R*)-2-isopropyl-5-methylcyclohexyl)oxy)-2-oxoethyl)-10-oxo-1-(2,2,2-trifluoroacetyl)-2,3,4,6a,7a,8,9,10,10a,10b-decahydro-1*H*-pyrrolo[3',4':4,5]furo[3,2-*b*]azocine-8-carboxylate (21*)**

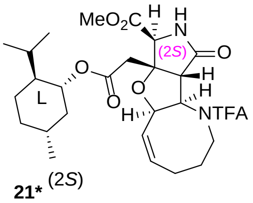

**21*** (2*S*)

To a stirred solution of the *N*-PMB amide **20*** (2*S*, 10.4 mg, 0.0153 mmol) in $CH_3CN$ (0.60 mL) and water (0.60 mL) at −10 °C was added CAN (125.9 mg, 0.2297 mmol). After 3.5 h, the mixture was diluted with EtOAc (1 mL) and poured into water (1 mL). The aqueous layer was separated and extracted with EtOAc (4×1 mL). The combined extracts were washed with brine (2 mL), dried over $Na_2SO_4$, filtered through a pad of silica gel (60 N, 1 g, EtOAc), and concentrated under reduced pressure at rt to a volume of ca. 1 mL. The residual solution was poured into saturated aqueous $Na_2S_2O_3$ (2 mL), and the mixture was vigorously stirred at rt. After 2 h, the mixture was extracted with EtOAc (4×1 mL). The combined extracts were washed with brine (2 mL), dried over $Na_2SO_4$, and concentrated under reduced pressure. The residue was purified by column chromatography on silica gel (60 N, 600 mg, EtOAc/hexane 8:2) to give the unprotected amide **21*** (2*S*, 7.5 mg, 88%) as a colorless oil: $[\alpha]^{24.6}_D$ +1.83 (*c* 3.47, $CHCl_3$); IR (ATR) 3350, 2954, 2927, 2872, 1698, 1456, 1372, 1202, 1146, 1094, 1010 $cm^{-1}$; $^1H$ NMR (selected for the major rotamer, 400 MHz, $CDCl_3$) δ 6.75 (m, 1H), 5.61-5.49 (m, 2H), 4.96 (d, *J* = 8.5 Hz, 1H), 4.54 (ddd, *J* = 10.9, 10.9, 4.3 Hz, 1H), 4.27 (s, 1H), 4.23 (d, *J* = 8.5 Hz, 1H), 4.08 (m, 1H), 3.74 (s, 3H), 3.48 (s, 1H), 3.46 (d, *J* = 17.3 Hz, 1H), 3.31 (m, 1H), 3.24 (d, *J* = 17.3 Hz, 1H), 2.22 (m, 1H), 1.99 (m, 1H), 1.91

(m, 1H), 1.82-1.68 (m, 3H), 1.64-1.54 (m, 2H), 1.41-1.25 (m, 2H), 1.03-0.87 (m, 2H), 0.87-0.77 (m, 7H), 0.69 (d, *J* = 6.6 Hz, 3H); $^{13}C$ NMR (selected for the major rotamer, 100 MHz, $CDCl_3$) δ 176.0, 169.6, 169.3, 156.7 (q, *J* = 36.5 Hz), 128.9, 128.1, 116.2 (q, *J* = 288.3 Hz), 87.4, 79.5, 75.1, 73.7, 64.1, 55.7, 52.5, 51.9, 46.6, 40.3, 40.2, 34.0, 31.3, 27.8, 26.2, 25.6, 23.4, 21.9, 20.5, 16.4; HRMS (ESI, positive) calcd for $C_{27}H_{37}F_3N_2O_7Na^+$ [$(M+Na)^+$] 581.2445, found 581.2448.

**Methyl (6a*S*,7a*R*,8*R*,10a*S*,10b*S*,*Z*)-7a-(2-(((1*R*,2*S*,5*R*)-2-isopropyl-5-methylcyclohexyl)oxy)-2-oxoethyl)-10-oxo-1-(2,2,2-trifluoroacetyl)-2,3,4,6a,7a,8,9,10,10a,10b-decahydro-1*H*-pyrrolo[3',4':4,5]furo[3,2-*b*]azocine-8-carboxylate (21)**

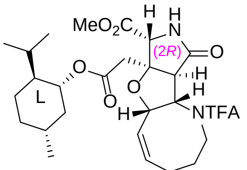


**21** (2*R*)

To a stirred solution of the *N*-PMB amide **20** (2*R*, 87.4 mg, 0.129 mmol) in $CH_3CN$ (5.0 mL) and water (5.0 mL) at −10 °C was added CAN (1.063 g, 1.939 mmol). After 3.5 h, the mixture was diluted with EtOAc (10 mL) and poured into water (10 mL). The aqueous layer was separated and extracted with EtOAc (4×10 mL). The combined extracts were washed with brine (20 mL), dried over $Na_2SO_4$, filtered through a pad of silica gel (60 N, 10 g, EtOAc), and concentrated under reduced pressure at rt to a volume of ca. 10 mL. The residual solution was poured into saturated aqueous $Na_2S_2O_3$ (20 mL), and the mixture was vigorously stirred at rt. After 2 h, the mixture was extracted with EtOAc (4×10 mL). The combined extracts were washed with brine (20 mL), dried over $Na_2SO_4$, and concentrated under reduced pressure. The residue was purified by column chromatography on silica gel (60 N, 2 g, EtOAc/hexane 5:5) to give the unprotected amide **21** (2*R*, 57.9 mg, 80%) as a colorless oil: $[\alpha]^{26.4}_D$ -52.5 (*c* 2.90, $CHCl_3$); IR (ATR) 3346, 2954, 2930, 2871, 1698, 1455, 1371, 1201, 1146, 1092, 1010 $cm^{-1}$; $^1H$ NMR (selected for the major rotamer, 400 MHz, $CDCl_3$) δ 6.78 (m, 1H),

5.61-5.46 (m, 2H), 4.97 (d, *J* = 8.1 Hz, 1H), 4.57 (ddd, *J* = 11.2, 11.2, 4.5 Hz, 1H), 4.28 (s, 1H), 4.24 (d, *J* = 8.1 Hz, 1H), 4.08 (m, 1H), 3.76 (s, 3H), 3.48 (d, *J* = 17.4 Hz, 1H), 3.48 (s, 1H), 3.29 (m, 1H), 3.26 (d, *J* = 17.4 Hz, 1H), 2.22 (m, 1H), 1.99 (m, 1H), 1.89 (m, 1H), 1.82-1.69 (m, 3H), 1.66-1.54 (m, 2H), 1.40 (m, 1H), 1.28 (m, 1H), 1.02-0.89 (m, 2H), 0.88-0.76 (m, 7H), 0.64 (d, *J* = 6.7 Hz, 3H); $^{13}C$ NMR (selected for the major rotamer, 100 MHz, $CDCl_3$) δ 176.0, 170.0, 169.5, 156.7 (q, *J* = 36.5 Hz), 128.9, 128.2, 116.2 (q, *J* = 288.1 Hz), 87.7, 79.5, 74.8, 73.9, 64.3, 55.6, 52.6, 51.9, 46.8, 40.7, 40.0, 34.1, 31.3, 27.8, 26.0, 25.6, 23.3, 22.0, 20.6, 16.1; HRMS (ESI, positive) calcd for $C_{27}H_{37}F_3N_2O_7Na^+$ [$(M+Na)^+$] 581.2445, found 581.2446.

**(6a*R*,7a*S*,8*S*,10a*R*,10b*R*,*Z*)-7a-(Carboxymethyl)-10-oxo-2,3,4,6a,7a,8,9,10,10a,10b-decahydro-1*H*-pyrrolo[3',4':4,5]furo[3,2-*b*]azocine-8-carboxylic acid ((2*S*)-TKM-38, 3*)**

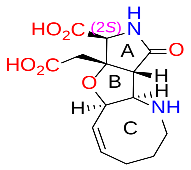


(2S)–**TKM–38** (**3***)

The ester amide **21*** (2*S*, 69.3 mg, 0.124 mmol) was dissolved in a solution of KOH in MeOH (1 M, 11.2 mL, 11.2 mmol). After stirring at 40 °C for 16 h, the mixture was poured into water (2 mL) and concentrated by blowing with air. The residue was dissolved in water (1 mL), neutralized with hydrochloric acid (12 M, 0.5 mL), and subjected to ion-exchange column chromatography (Dowex® 50W x8-200, $H^+$ form, 12.5 g). The column was washed with water until the eluate became neutral and then eluted with ammonium hydroxide (5 M). Positive fractions in the ninhydrin test were combined and concentrated by blowing with air to give the glutamate analog (2*S*)-TKM-38 (**3***, 29.6 mg, 77%) as a brown oil: $[\alpha]^{24.9}_D$ -31.4 (*c* 1.20, MeOH/$H_2O$ = 1:1); IR (ATR) 3093, 3069, 3046, 1697, 1573, 1395, 1291, 1185, 1121, 1073, 1007 $cm^{-1}$; $^1H$ NMR (TFA salt, 400 MHz, $D_2O$) δ 5.90 (m, 1H), 5.51 (brdd, *J* = 11.8, 3.7 Hz, 1H), 5.03 (s, 1H), 4.41 (s,

1H), 4.24 (dd, $J$ = 5.8, 2.8 Hz, 1H), 3.68 (d, $J$ = 2.8 Hz, 1H), 3.48 (ddd, $J$ = 14.1, 4.9, 4.9 Hz, 1H), 3.24 (ddd, $J$ = 14.1, 10.1, 4.2 Hz, 1H), 3.18 (d, $J$ = 17.3 Hz, 1H), 3.08 (d, $J$ = 17.3 Hz, 1H), 2.34-2.13 (m, 2H), 1.92 (m, 1H), 1.74 (m, 1H); $^{13}$C NMR (100 MHz, $D_2O$) δ 177.0, 174.7, 173.9, 133.8, 124.4, 84.5, 77.8, 68.2, 63.1, 56.1, 45.5, 44.8, 23.6, 23.1; HRMS (ESI, positive) calcd for $C_{14}H_{19}N_2O_6^+$ [(M+H)$^+$] 311.1228, found 311.1237.

**(6a*S*,7a*R*,8*R*,10a*S*,10b*S*,*Z*)-7a-(Carboxymethyl)-10-oxo-2,3,4,6a,7a,8,9,10,10a,10b-decahydro-1*H*-pyrrolo[3',4':4,5]furo[3,2-*b*]azocine-8-carboxylic acid ((2*R*)-TKM-38, 3)**

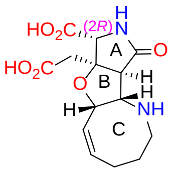


(2*R*)–TKM–38 (3)

The ester amide **21** (2*R*, 4.3 mg, 0.0078 mmol) was dissolved in a solution of KOH in MeOH (1 M, 0.700 mL, 0.700 mmol). After stirring at 40 °C for 18 h, the mixture was poured into water (1 mL) and concentrated by blowing with air. The residue was dissolved in water, neutralized with hydrochloric acid (12 M, 0.1 mL), and subjected to ion-exchange column chromatography (Dowex® 50W x8-200, H$^+$ form, 800 mg). The column was washed with water until the eluate became neutral and then eluted with ammonium hydroxide (5 M). Positive fractions in the ninhydrin test were combined and concentrated by blowing with air to give the glutamate analog (2*R*)-TKM-38 (**3**, 2.2 mg, 90%) as a brown oil: $[\alpha]^{25.0}_D$ +31.1 (*c* 1.01, MeOH/$H_2O$ = 1:1).

The other spectroscopic data were in good agreement with those for **3*** (see above).

## References

[1] M. Oikawa, M. Ikoma, M. Sasaki, M.B. Gill, G.T. Swanson, K. Shimamoto, R. Sakai, Eur. J. Org. Chem. 2009 (2009) 5531-5548.
