# Supplementary material for: Menthyl esterification allows chiral resolution for the synthesis of artificial glutamate analogs
Source: Beilstein J Org Chem. 2021 Feb 24;17:540–50. doi: 10.3762/bjoc.17.48 (PMC7934734; doi:10.3762/bjoc.17.48)
Supplement: File 2 — NMR spectra of all new compounds. [file Beilstein_J_Org_Chem-17-540-s002.pdf]

BEILSTEIN JOURNAL OF ORGANIC CHEMISTRY

Supporting Information

for

Menthyl esterification allows chiral resolution for the synthesis of artificial glutamate analogs

Kenji Morokuma, Shuntaro Tsukamoto, Kyosuke Mori, Kei Miyako, Ryuichi Sakai, Raku Irie and Masato Oikawa

Beilstein J. Org. Chem. **2021,** *17,* 540–550. doi:10.3762/bjoc.17.48

NMR spectra of all new compounds

License and Terms: This is a supporting information file under the terms of the Creative Commons Attribution License (https://creativecommons.org/licenses/by/4.0). Please note that the reuse, redistribution and reproduction in particular requires that the author(s) and source are credited and that individual graphics may be subject to special legal provisions.
The license is subject to the *Beilstein Journal of Organic Chemistry* terms and conditions: (https://www.beilstein-journals.org/bjoc/terms)

Contents:

NMR spectra for all new compounds **SII-2 ~ SII-39**

SII-2

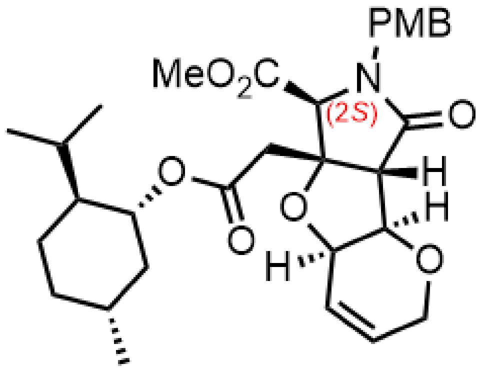

9* (2*S*)

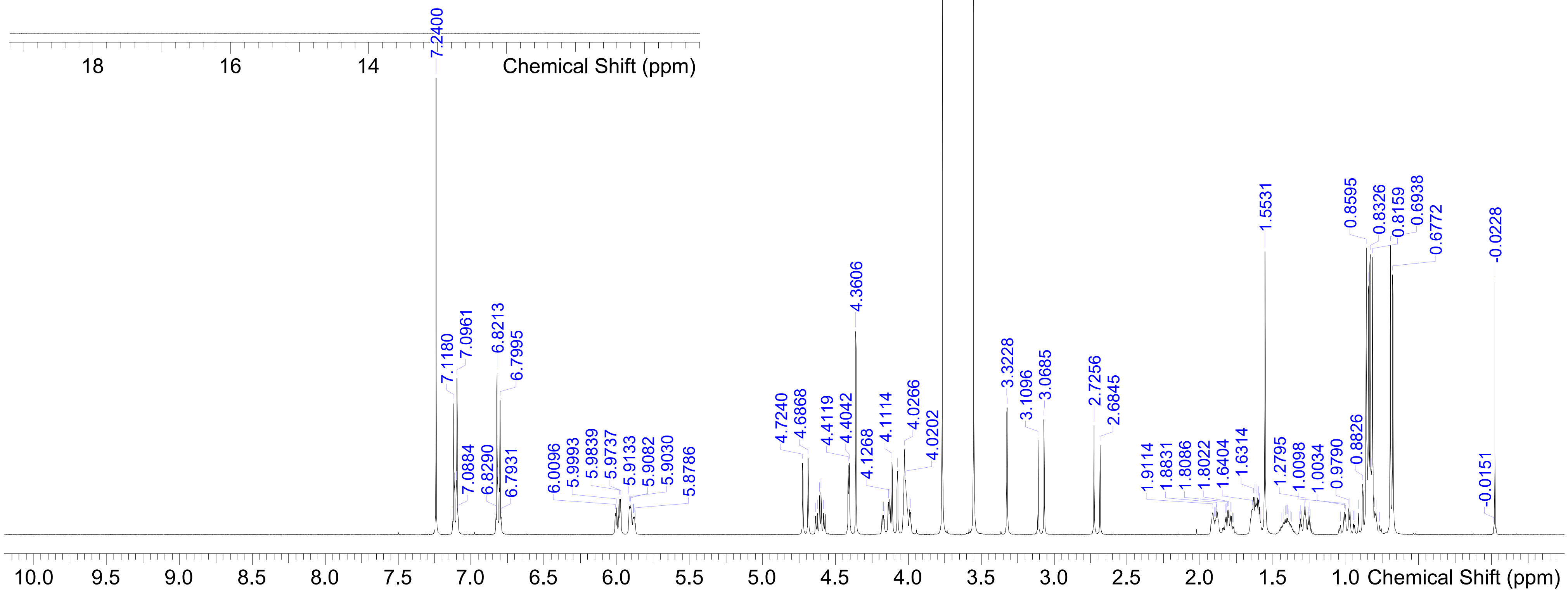

Date 29 Jan 2018 20:04:36

AA70061-002

**Nucleus**	1H
**Pulse Sequence**	zg30_Bruker_AVIIIHD400N-2
**Solvent**	CHLOROFORM-d

**Acquisition Time (sec)**	1.9464
**Frequency (MHz)**	400.0300
**Receiver Gain**	202.37
**Sweep Width (Hz)**	8417.00

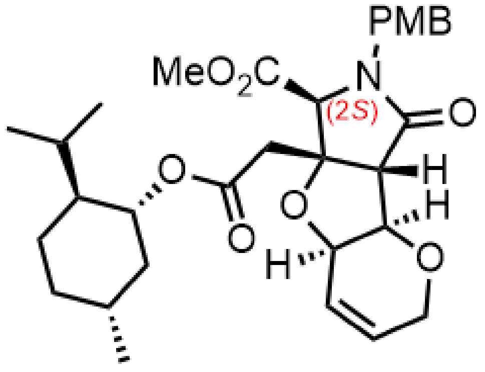

9* (2*S*)

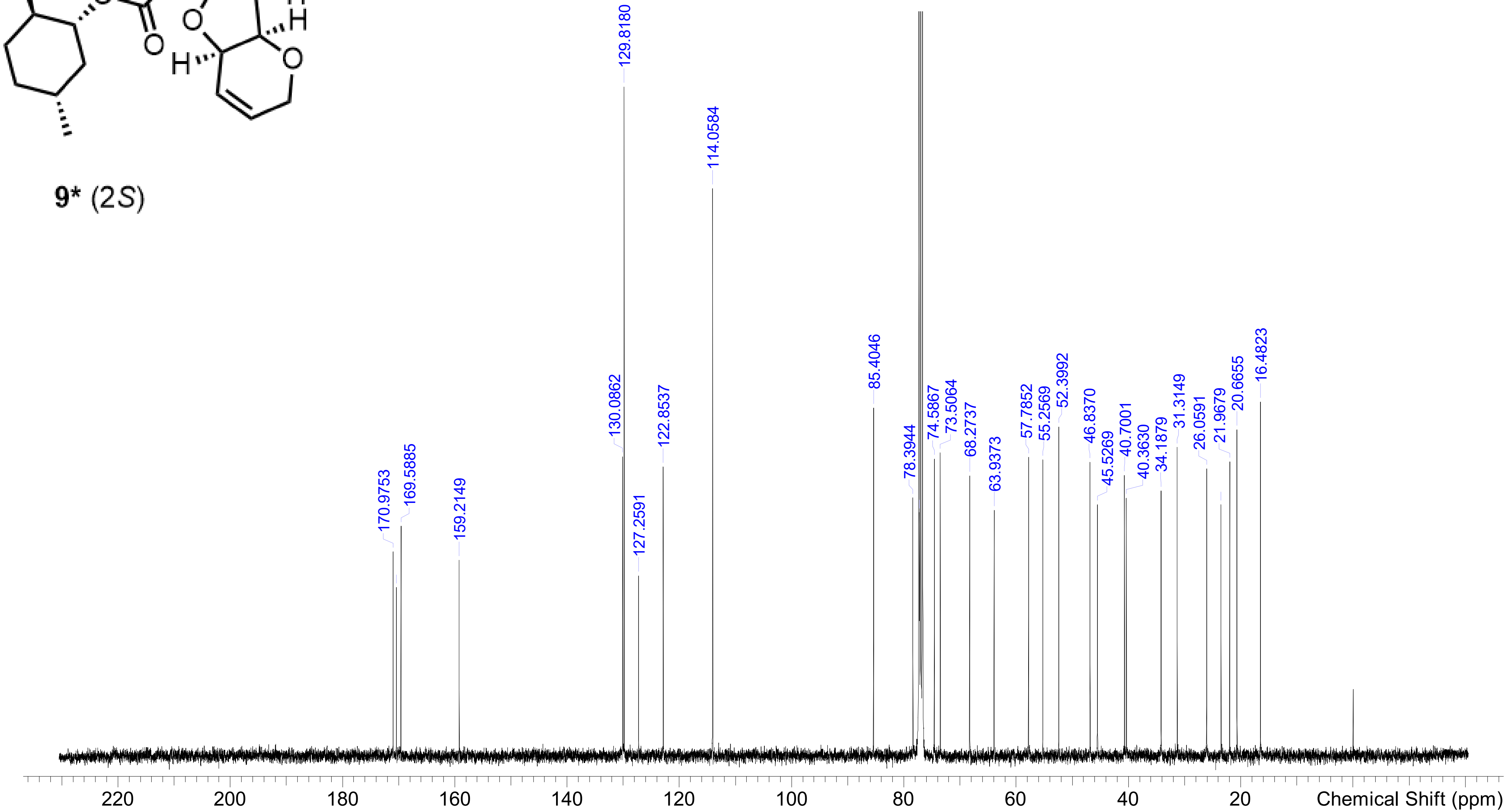

Date 30 Jan 2018 07:23:28

AA70061-002

**Nucleus**	13C
**Pulse Sequence**	zgpg30_Bruker_AVIIIHD400N-2
**Solvent**	CHLOROFORM-d
**Acquisition Time (sec)**	1.2976
**Frequency (MHz)**	100.5876
**Receiver Gain**	202.37
**Sweep Width (Hz)**	25251.75

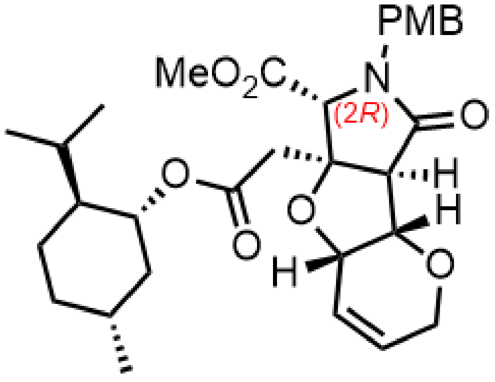

9 (2*R*)

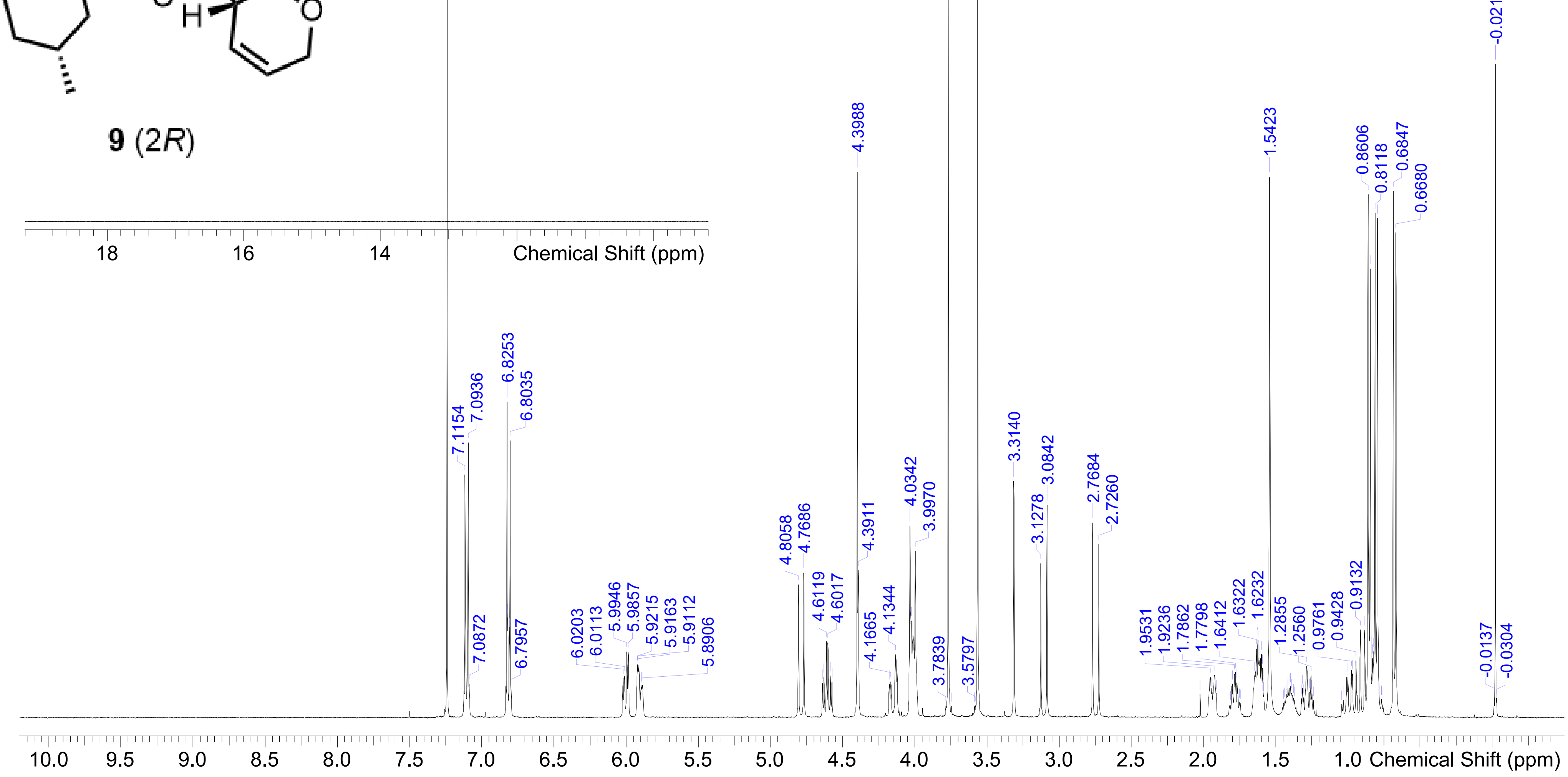

Date 23 Jan 2018 17:52:55

AA70051-003

**Nucleus**	1H
**Pulse Sequence**	zg30_Bruker_AVANCEIII400N
**Solvent**	CHLOROFORM-d

**Acquisition Time (sec)**	1.9464
**Frequency (MHz)**	400.1800
**Receiver Gain**	128.00
**Sweep Width (Hz)**	8417.00

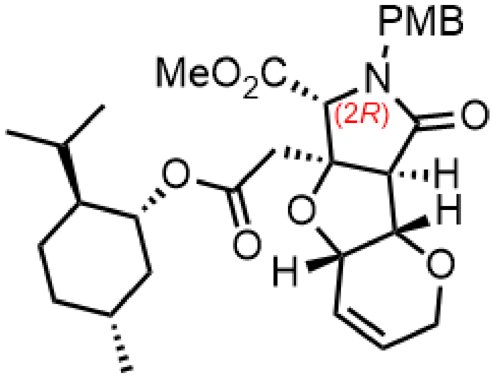

9 (2*R*)

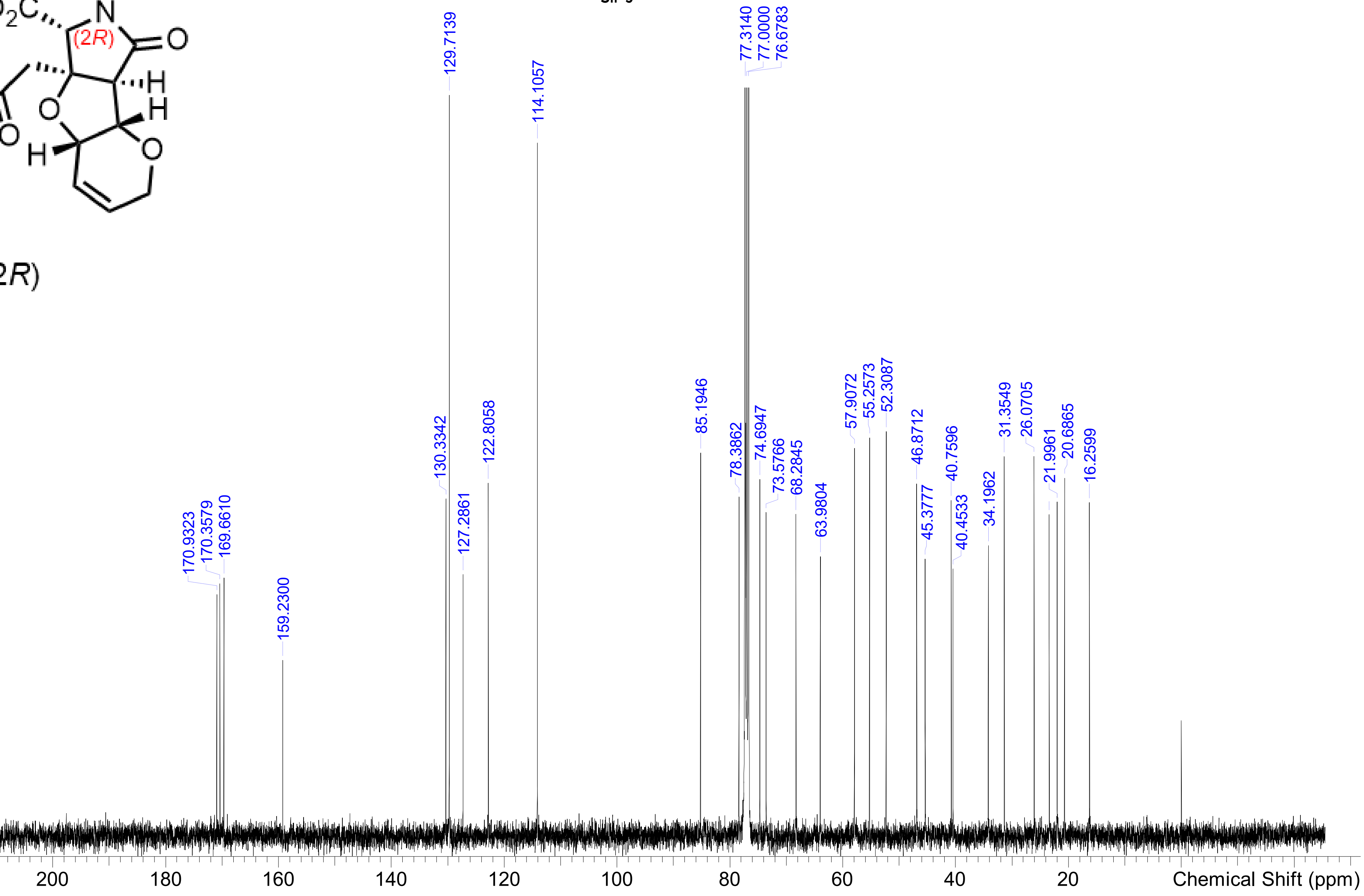

Date 24 Jan 2018 05:25:30

AA70051-003

**Nucleus**	13C
**Pulse Sequence**	zgpg30_Bruker_AVANCEIII400N
**Solvent**	CHLOROFORM-d

**Acquisition Time (sec)**	1.2976
**Frequency (MHz)**	100.6253
**Receiver Gain**	203.00
**Sweep Width (Hz)**	25251.75

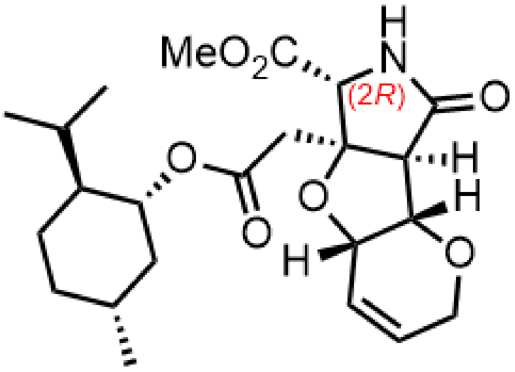

10 (2*R*)

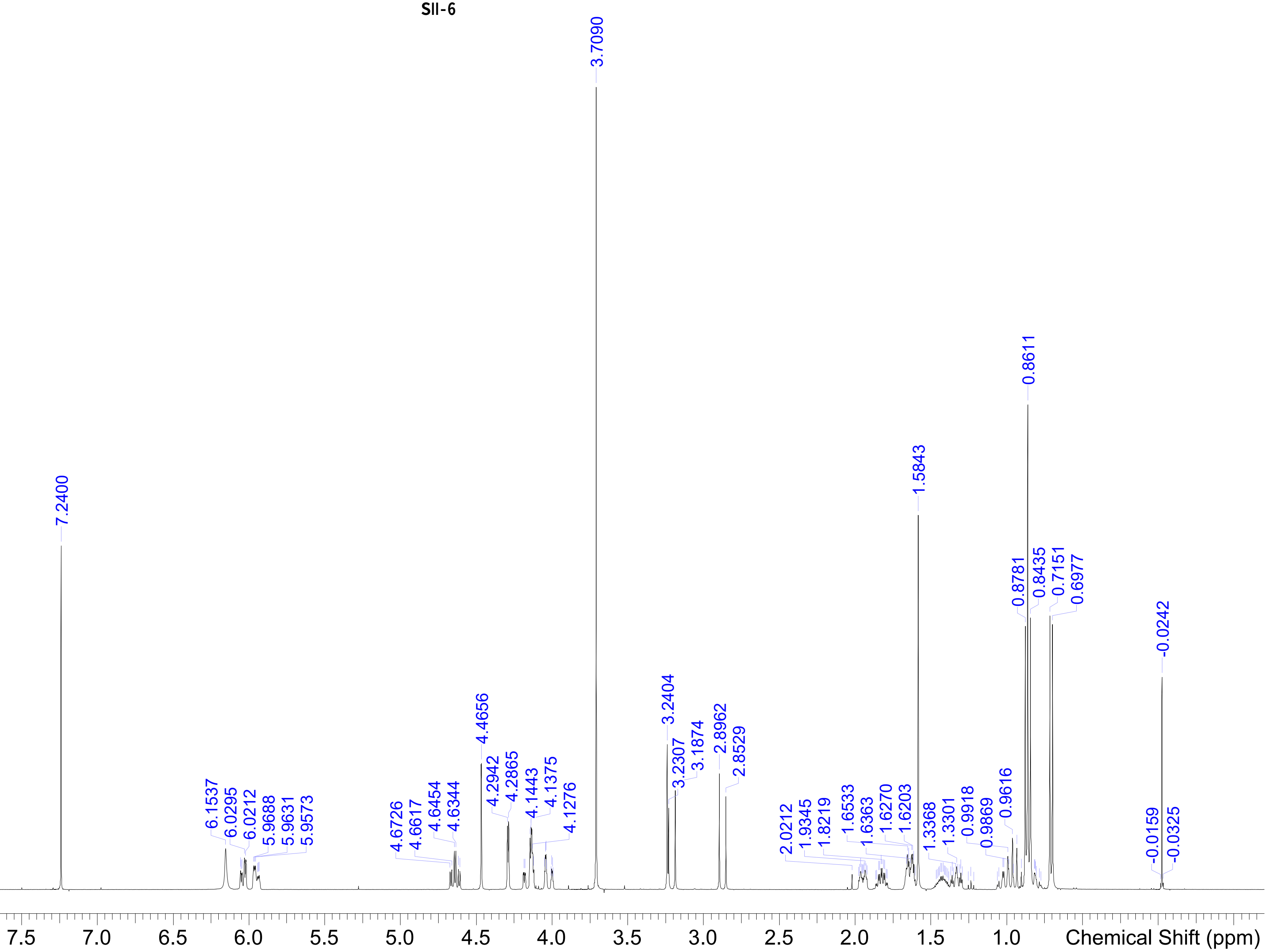

Date 16 Nov 2020 22:05:37

Kenji Morokuma
AA70065-002

**Nucleus**	1H	**Acquisition Time (sec)**	3.8928	**D1**	1
**Pulse Sequence**	zg30_Bruker_AVANCEII400M	**Frequency (MHz)**	400.1300	**NS**	32
**Solvent**	CHLOROFORM-d	**Receiver Gain**	406.00	**SI**	65536
		**Sweep Width (Hz)**	8417.38	**TD**	65536

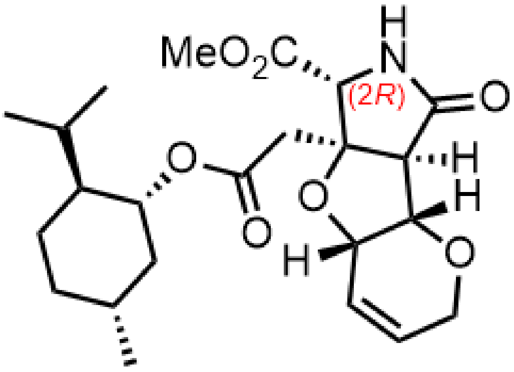

10 (2*R*)

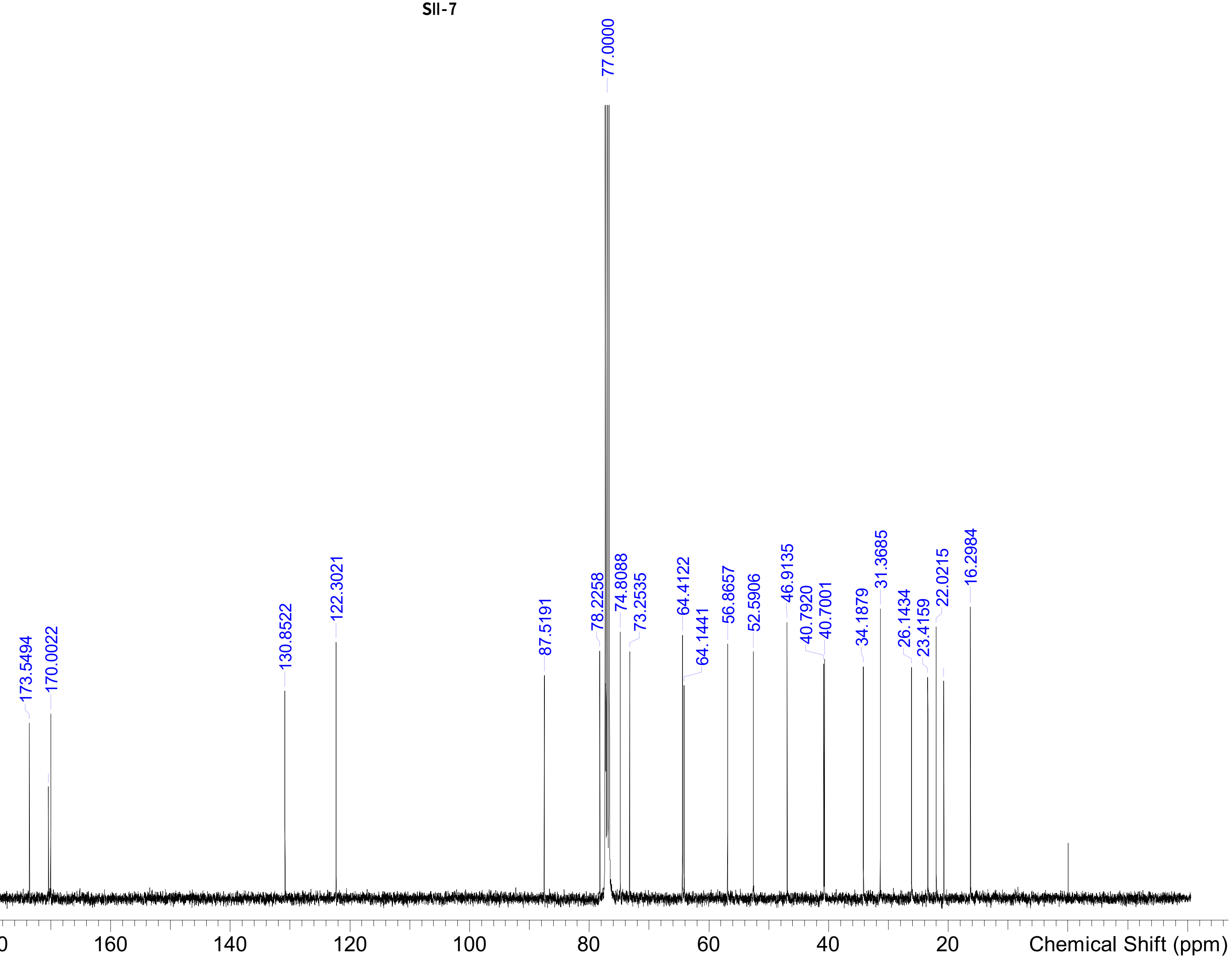

Date 24 Jan 2018 07:30:39

AA70052-001

Nucleus 13C
Pulse Sequence zgpg30_Bruker_AVIIIHD400N-2
Solvent CHLOROFORM-d

Acquisition Time (sec) 1.2976
Frequency (MHz) 100.5876
Receiver Gain 202.37
Sweep Width (Hz) 25251.75

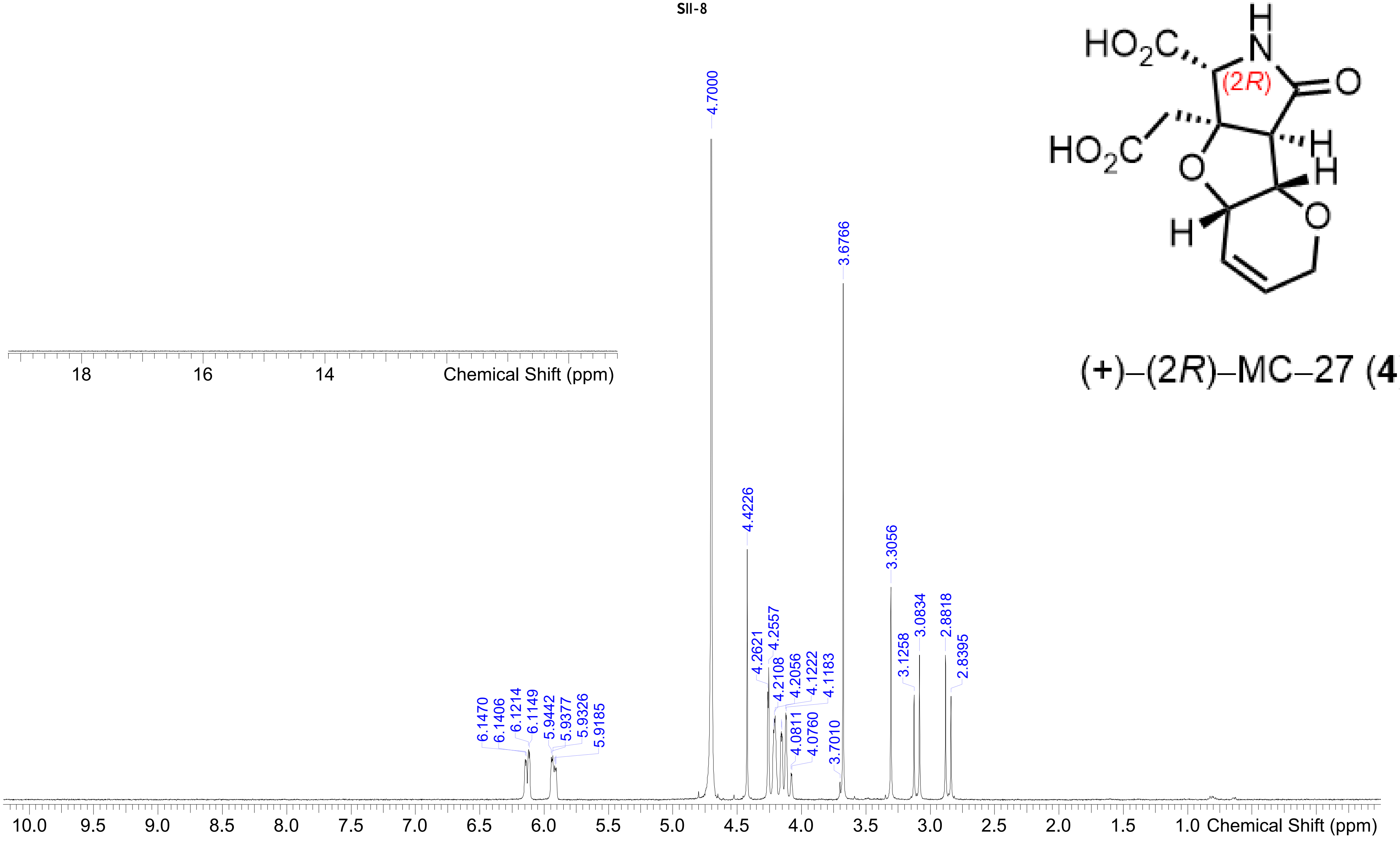

(+)–(2*R*)–MC–27 (**4**)

Date 23 Jan 2018 13:48:34

AA70069-001

**Nucleus**	1H
**Pulse Sequence**	zg30_Bruker_AVANCEII400M
**Solvent**	DEUTERIUM OXIDE

**Acquisition Time (sec)**	1.9464
**Frequency (MHz)**	400.1300
**Receiver Gain**	724.00
**Sweep Width (Hz)**	8417.00

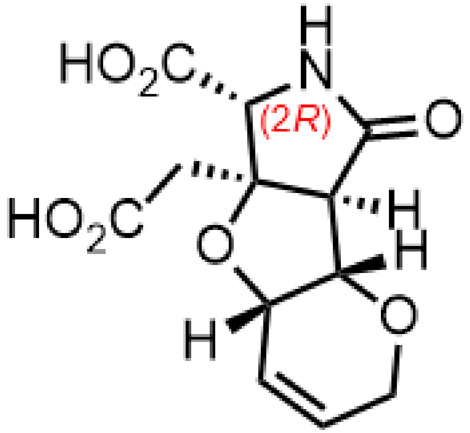

(+)–(2*R*)–MC–27 (**4**)

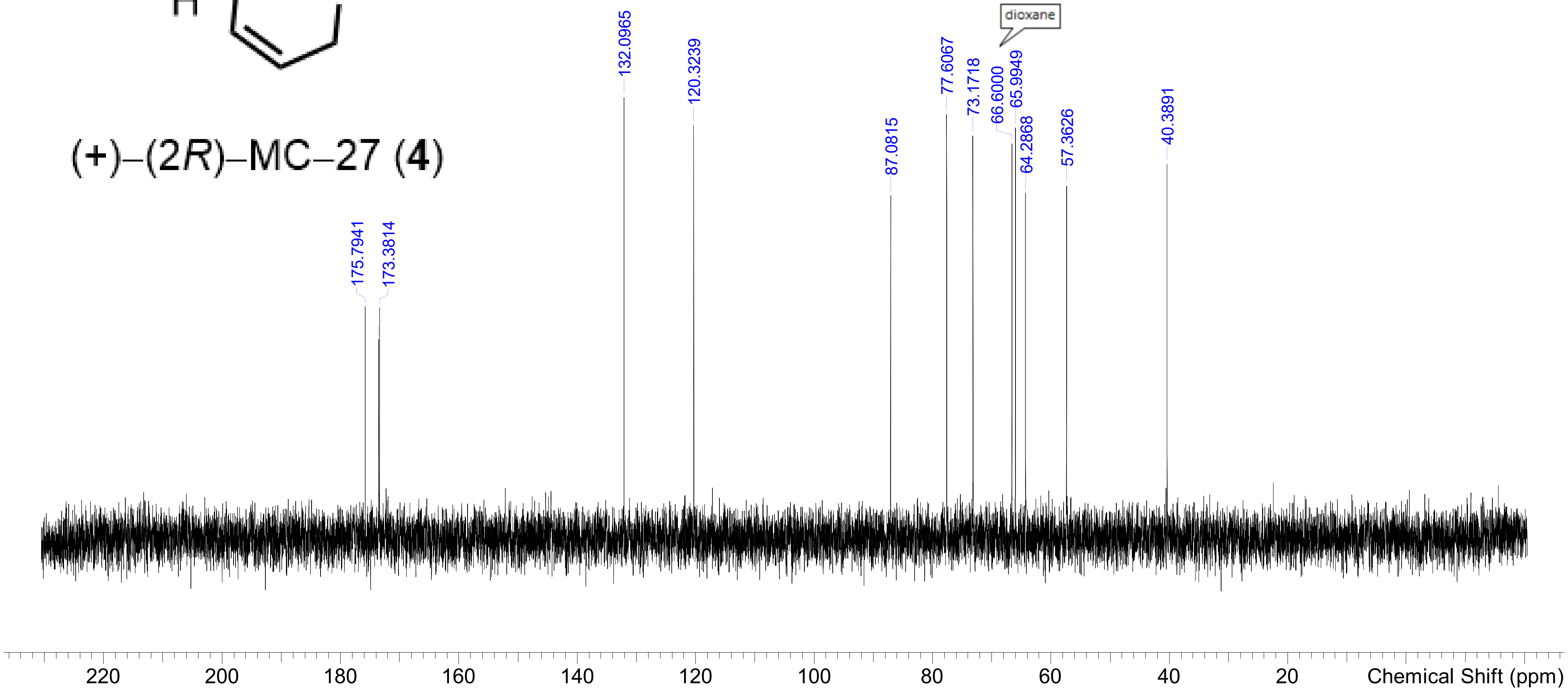

Date 24 Jan 2018 07:32:40

AA70069-001

**Nucleus**	13C
**Pulse Sequence**	zgpg30_Bruker_AVANCEII400M
**Solvent**	DEUTERIUM OXIDE

**Acquisition Time (sec)**	1.2976
**Frequency (MHz)**	100.6128
**Receiver Gain**	2050.00
**Sweep Width (Hz)**	25251.75

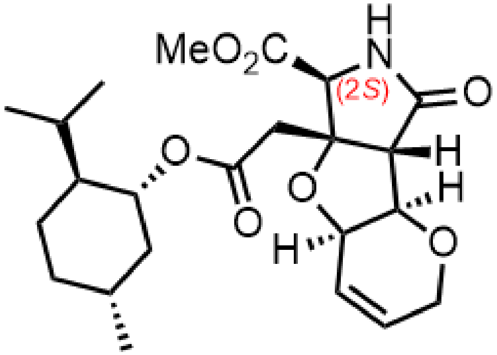

10* (2*S*)

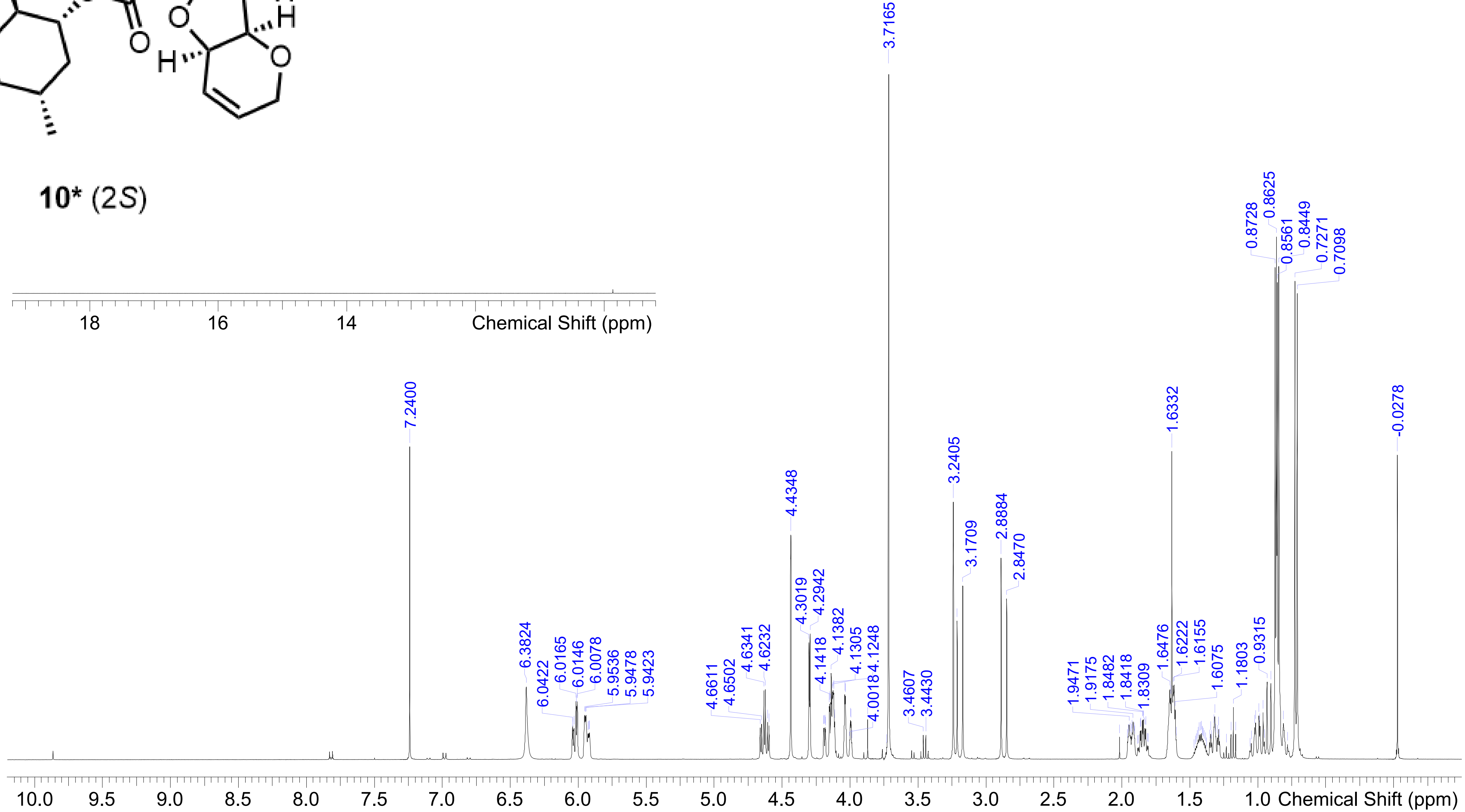

**Date** 20 Mar 2019 20:14:25	**Nucleus**	1H	**Acquisition Time (sec)**	3.8928	**D1** 1
Kenji Morokuma	**Pulse Sequence**	zg30_Bruker_AVANCEIII400N	**Frequency (MHz)**	400.1800	**NS** 16
AA70071-001	**Solvent**	CHLOROFORM-d	**Receiver Gain**	71.80	**SI** 65536
			**Sweep Width (Hz)**	8417.38	**TD** 65536

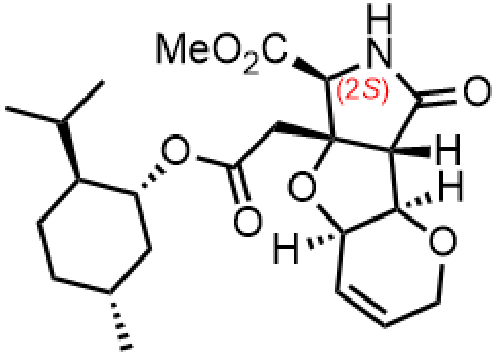

10* (2*S*)

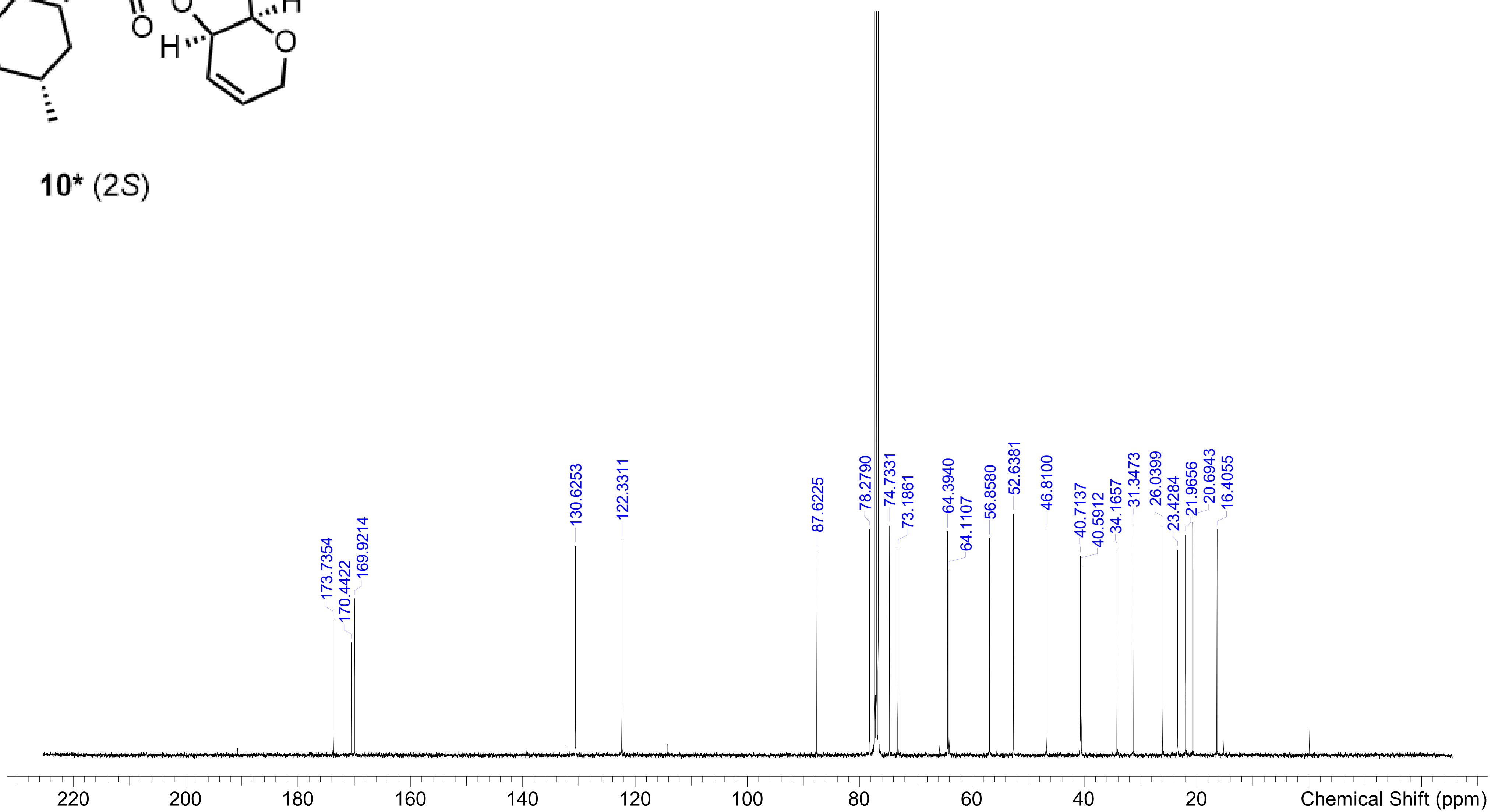

Date 21 Mar 2019 03:54:45

Kenji Morokuma
AA70071-001

Nucleus 13C
Pulse Sequence zgpg30_Bruker_AVANCEIII400N
Solvent CHLOROFORM-d

Acquisition Time (sec) 1.2976
Frequency (MHz) 100.6253
Receiver Gain 203.00
Sweep Width (Hz) 25251.75

D1 2
NS 8192
SI 32768
TD 65536

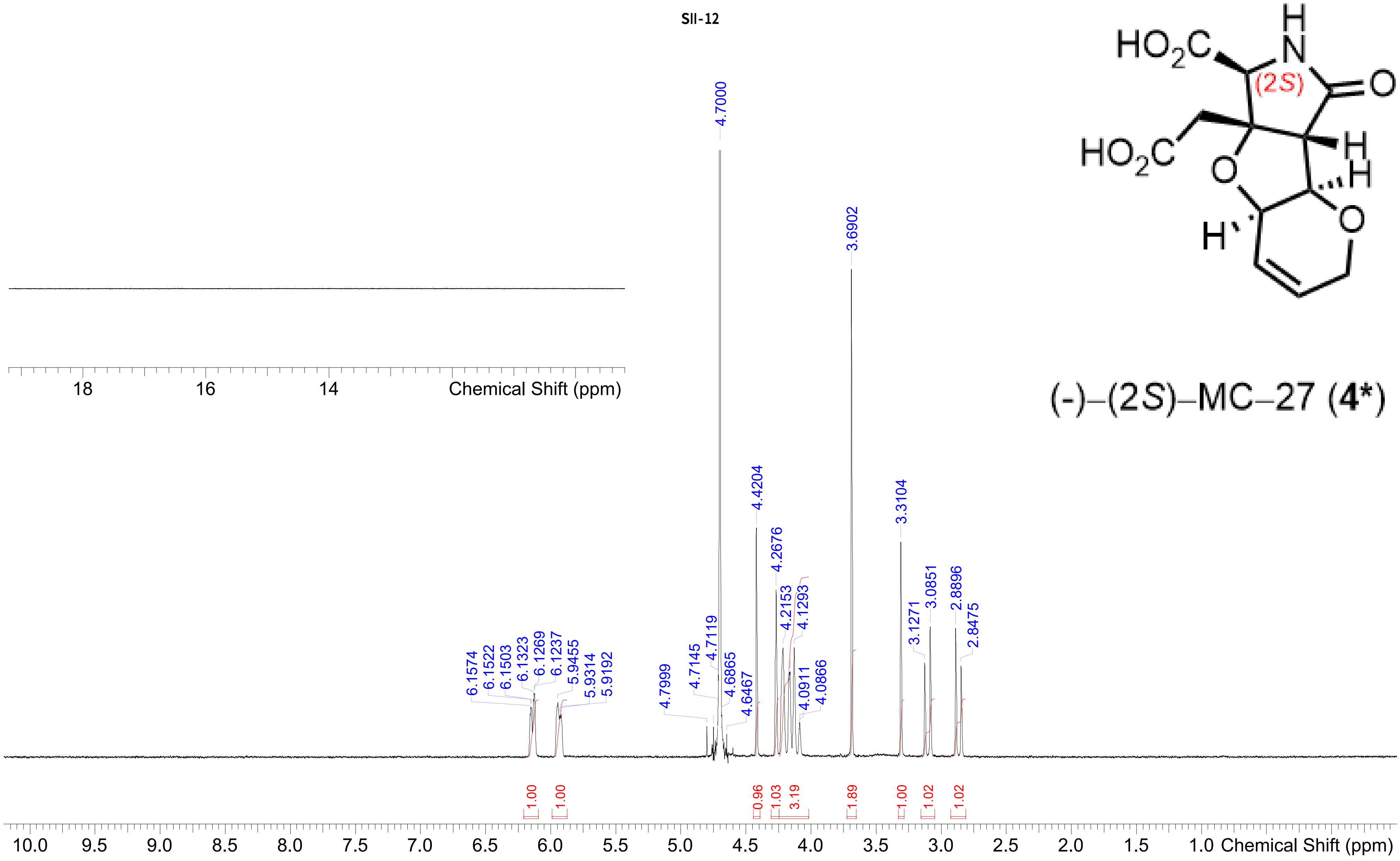

(-)–(2*S*)–MC–27 (**4***)

Date 17 Jan 2020 16:34:58

Kenji Morokuma
AA70073-001

**Nucleus**	1H	**Acquisition Time (sec)**	3.8928
**Pulse Sequence**	zg30_Bruker_AVANCEII400M	**Frequency (MHz)**	400.1300
**Solvent**	DEUTERIUM OXIDE	**Receiver Gain**	724.00
		**Sweep Width (Hz)**	8417.38

**D1**	1
**NS**	64
**SI**	65536
**TD**	65536

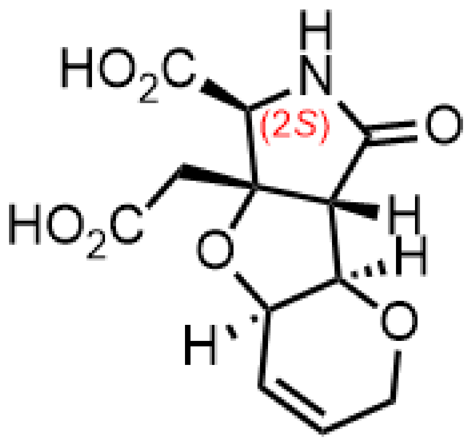

(-)–(2*S*)–MC–27 (**4***)

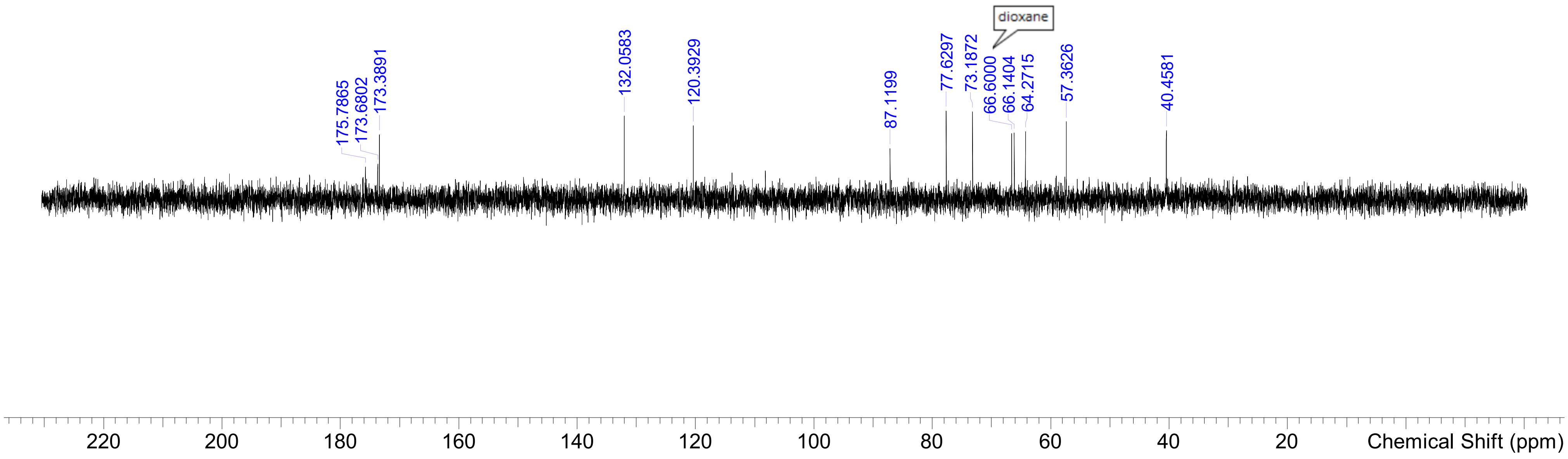

Date 18 Jan 2020 01:55:11

Kenji Morokuma
AA70073-001

**Nucleus**	13C	**Acquisition Time (sec)**	1.2976	**D1**	2
**Pulse Sequence**	zgpg30_Bruker_AVANCEII400M	**Frequency (MHz)**	100.6128	**NS**	4096
**Solvent**	DEUTERIUM OXIDE	**Receiver Gain**	512.00	**SI**	32768
		**Sweep Width (Hz)**	25251.75	**TD**	65536

NOESY spectrum of menthyl ester diastereomer 10

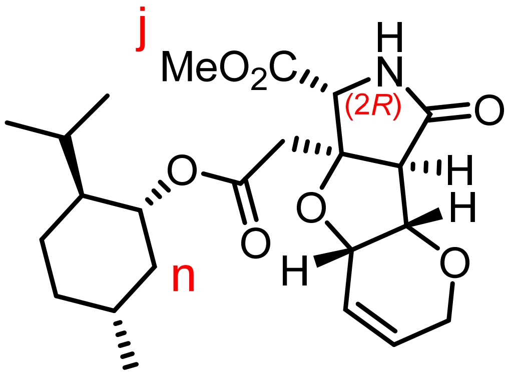

10 (2*R*)

fig. NOESY (400 MHz, CDCl3)

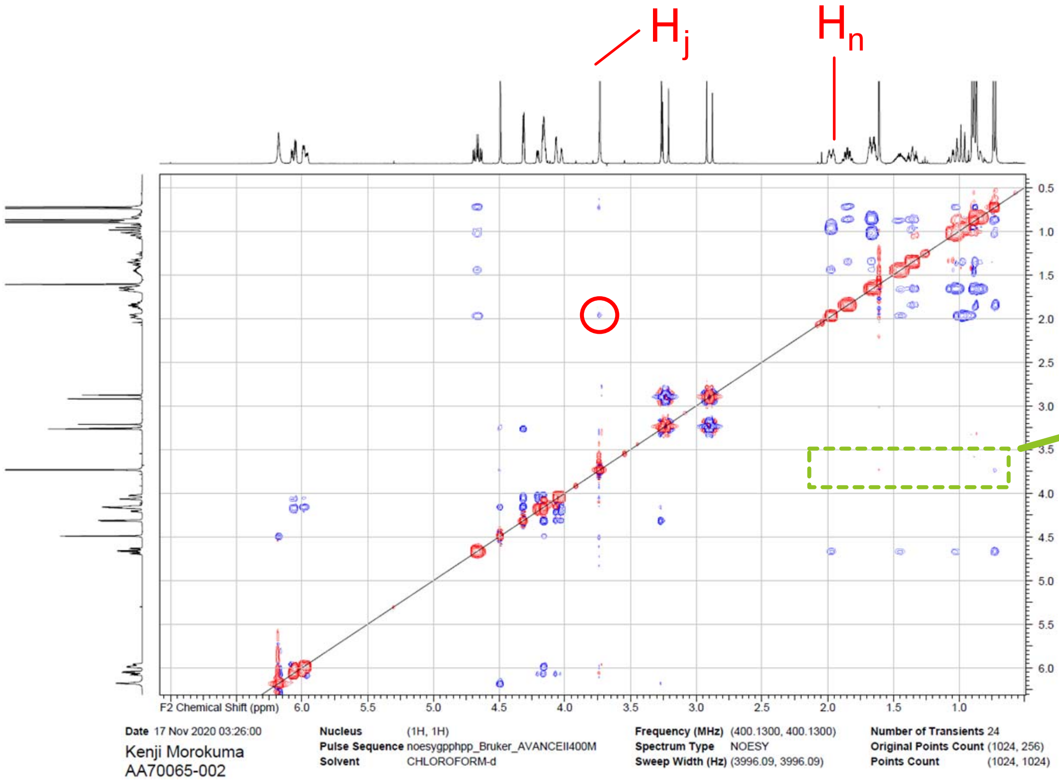

a spectrum deeply digged by a low threshold

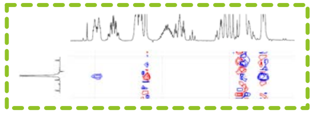

NOESY spectrum of menthyl ester diastereomer 10*

fig. NOESY (400 MHz, CDCl3)

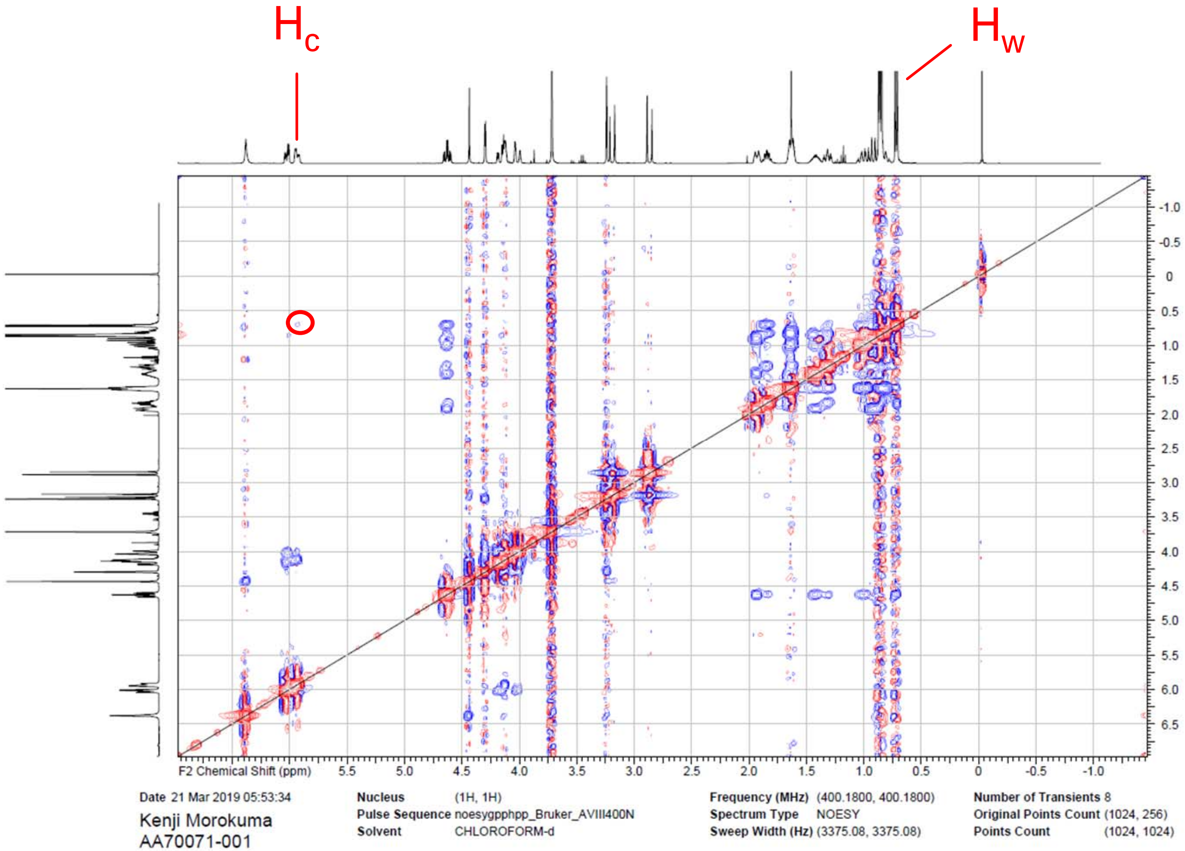

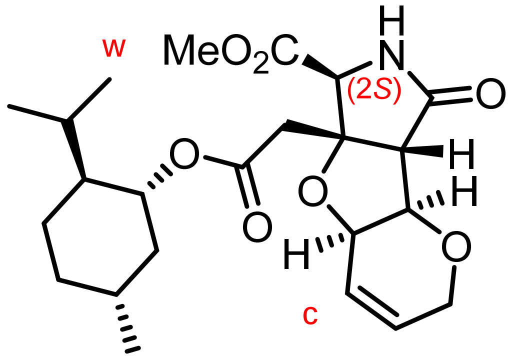

10* (2*S*)

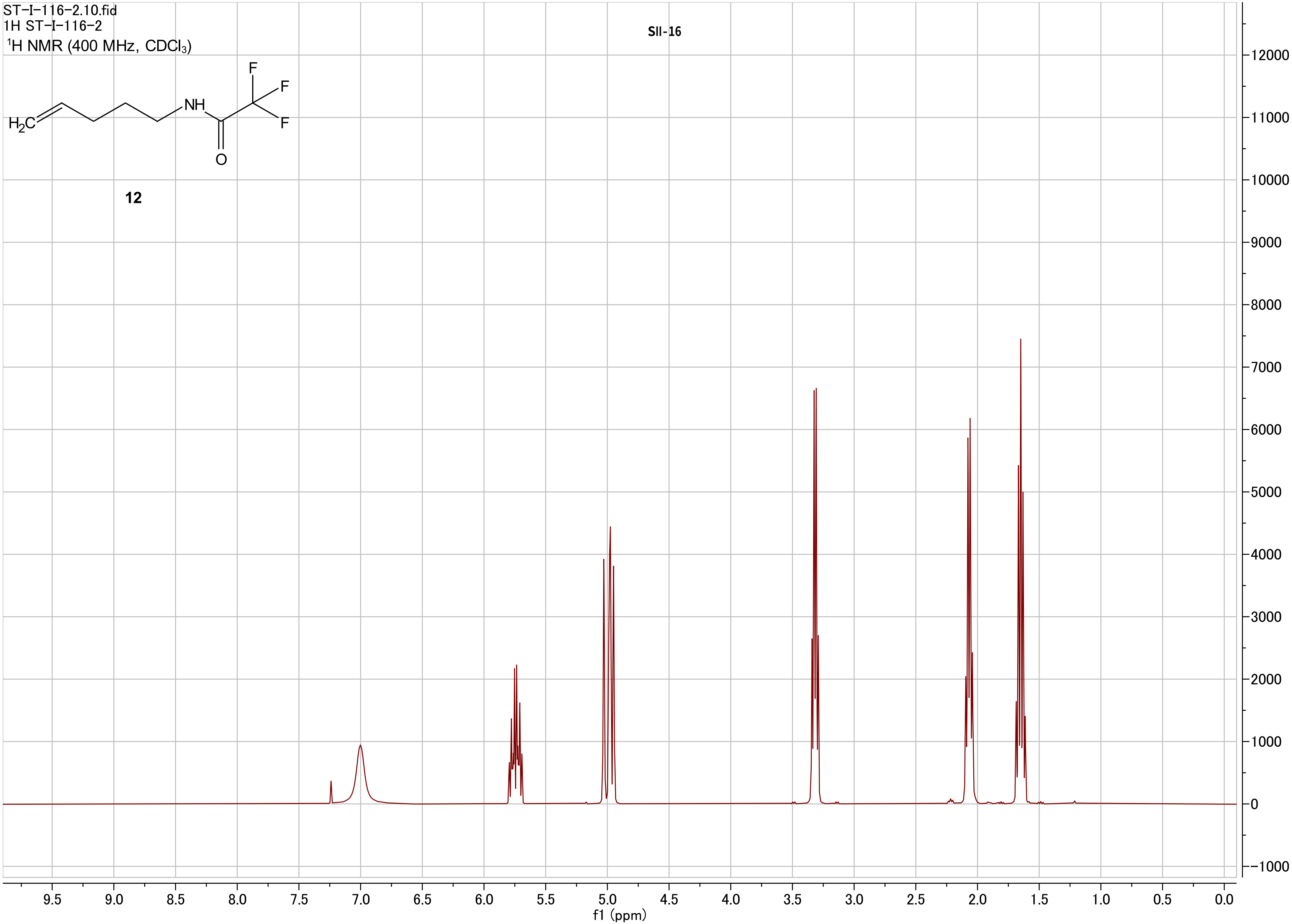

12

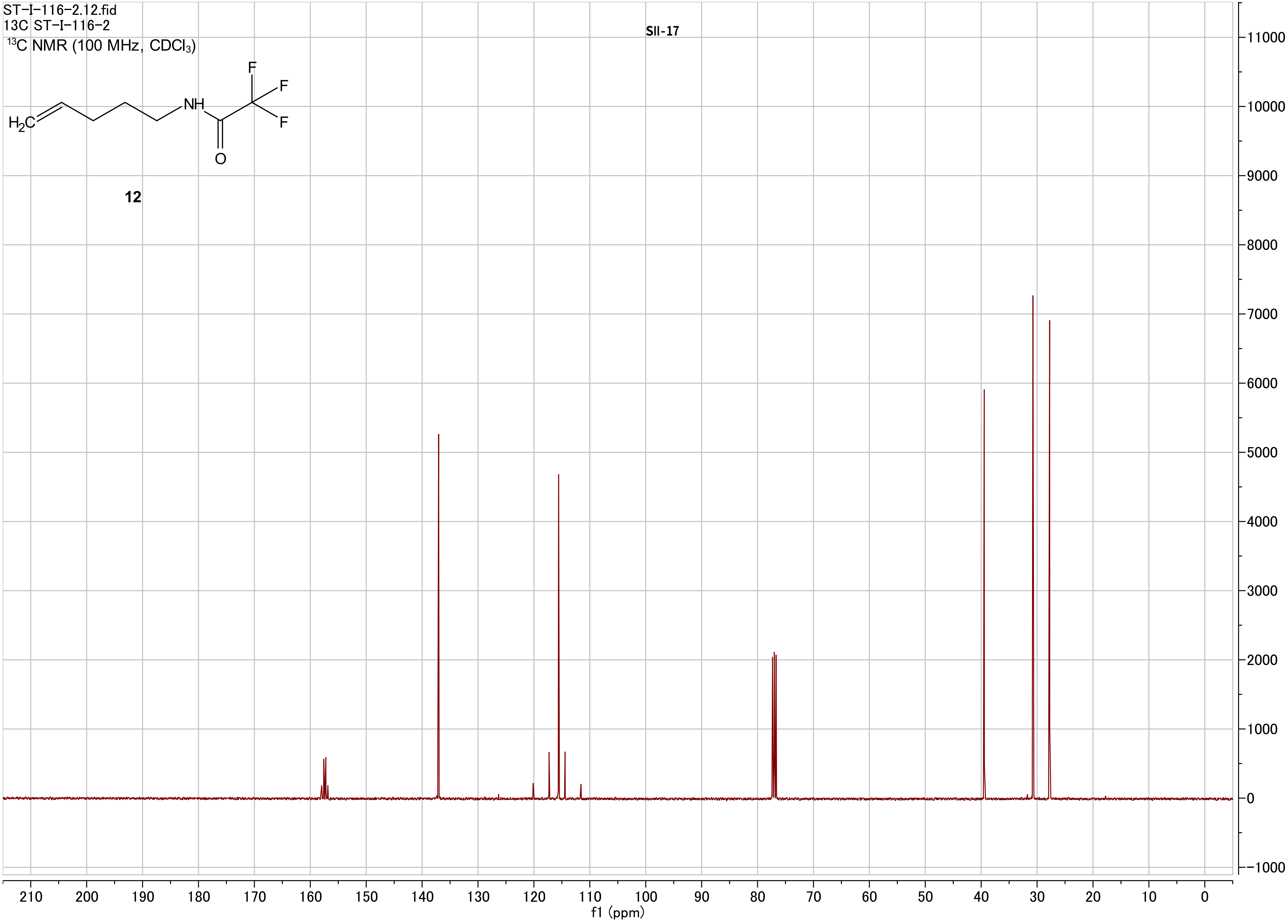
ST-I-116-2.12.fid
13C ST-I-116-2
13C NMR (100 MHz, CDCl3)
H2C
NH
F
F
F
O
12
f1 (ppm)
210
200
190
180
170
160
150
140
130
120
110
100
90
80
70
60
50
40
30
20
10
0
11000
10000
9000
8000
7000
6000
5000
4000
3000
2000
1000
0
-1000

ST-I-117-1.10.fid
1H ST-I-117-1
^{1}H NMR (400 MHz, $CDCl_3$)

SII-18

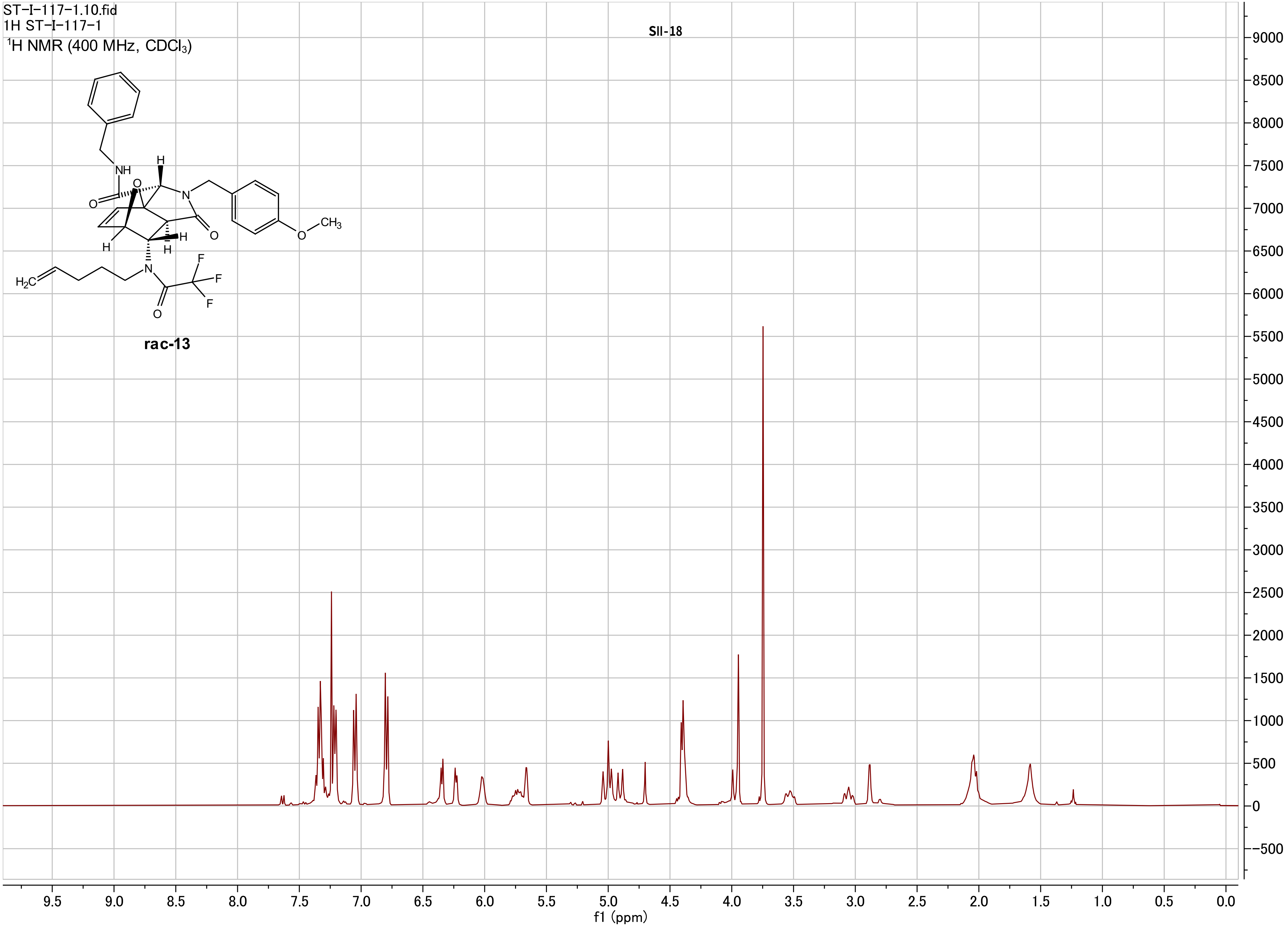

rac-13

ST-I-126-1.30.fid
13C ST-I-126-1
^{13}C NMR (100 MHz, $CDCl_3$)

SII-19

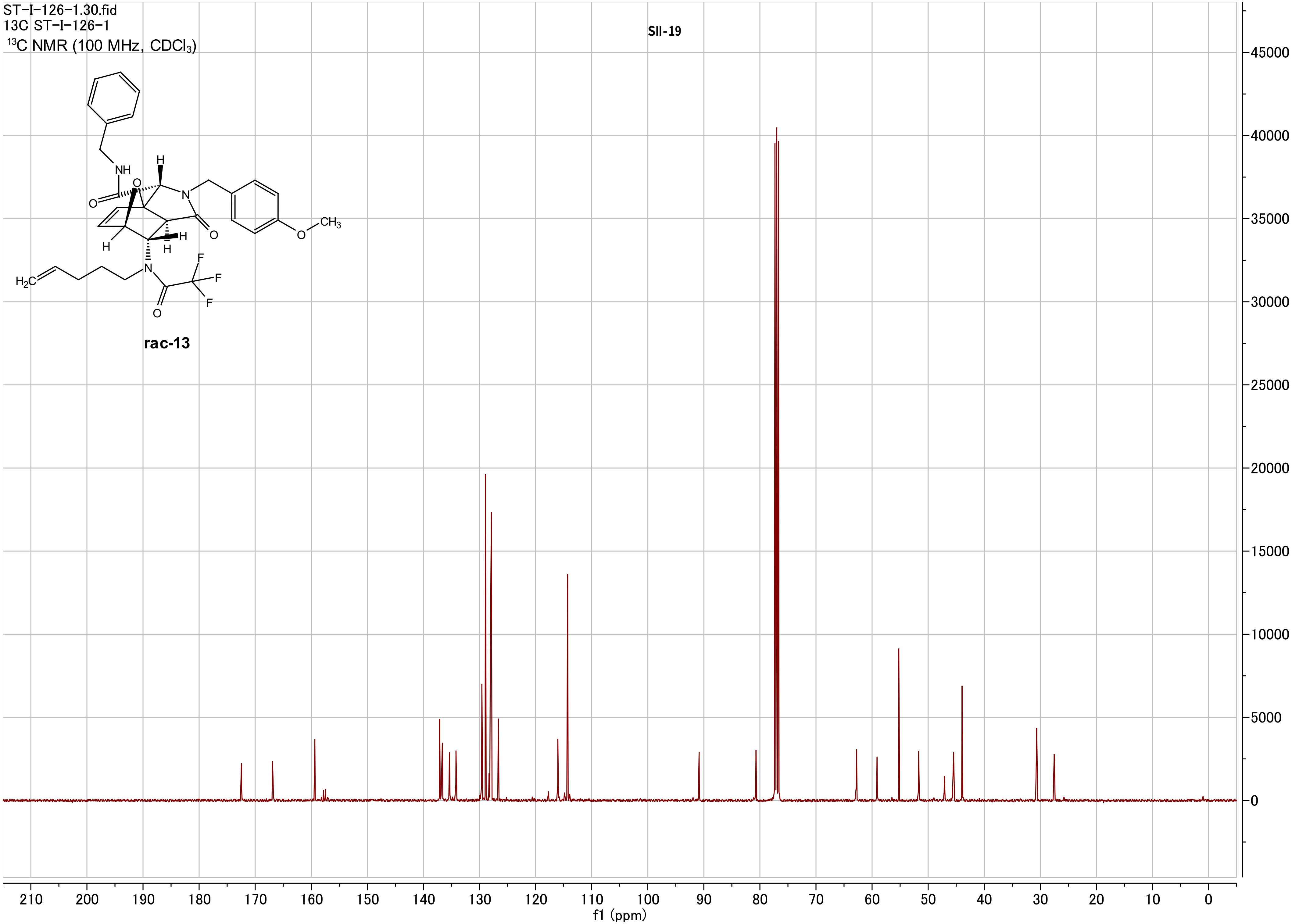

rac-13

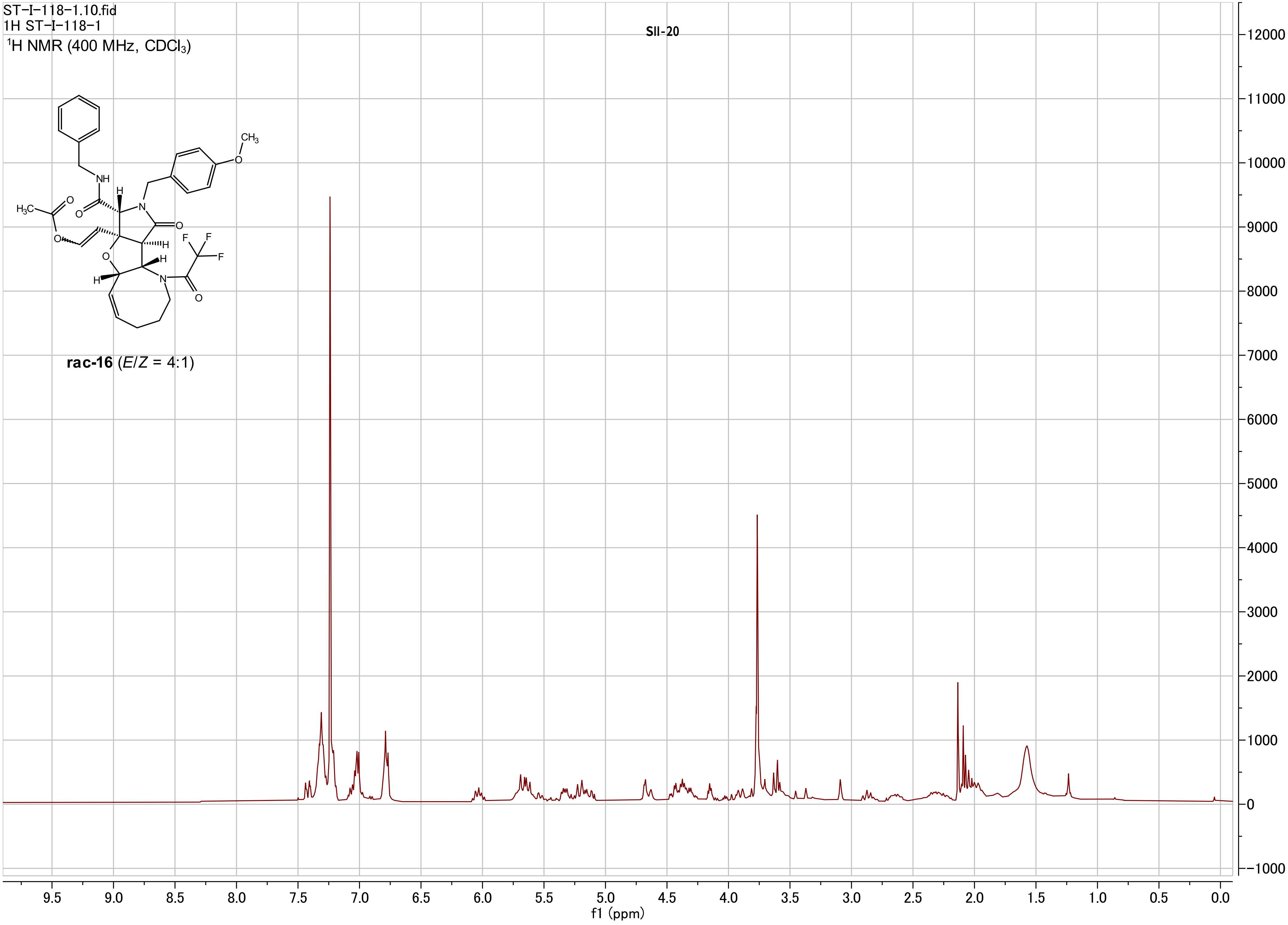
ST-I-118-1.10.fid
1H ST-I-118-1
^{1}H NMR (400 MHz, $CDCl_3$)
SII-20
rac-16 (E/Z = 4:1)
f1 (ppm)

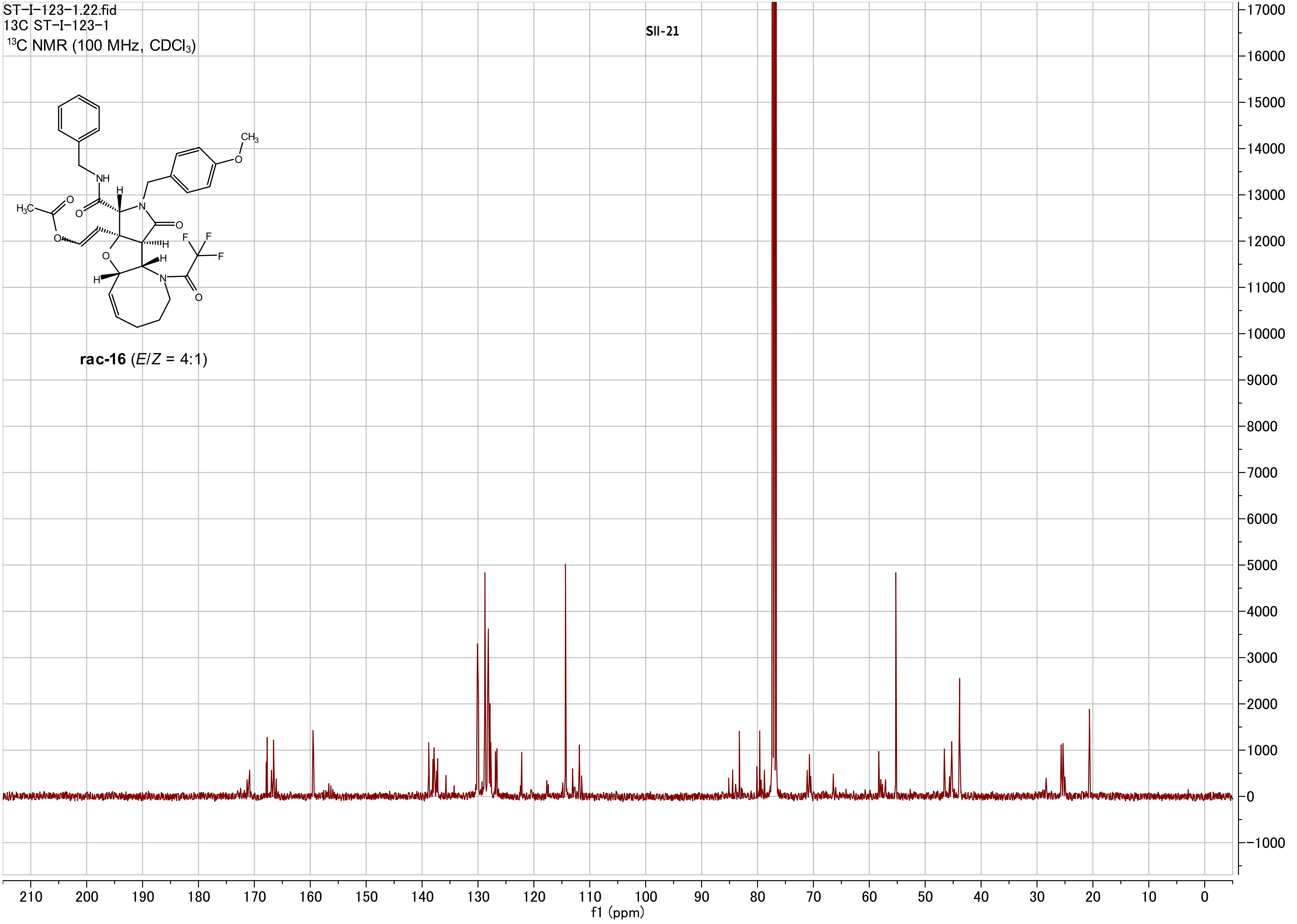
ST-I-123-1.22.fid
13C ST-I-123-1
13C NMR (100 MHz, CDCl3)
rac-16 (E/Z = 4:1)
f1 (ppm)

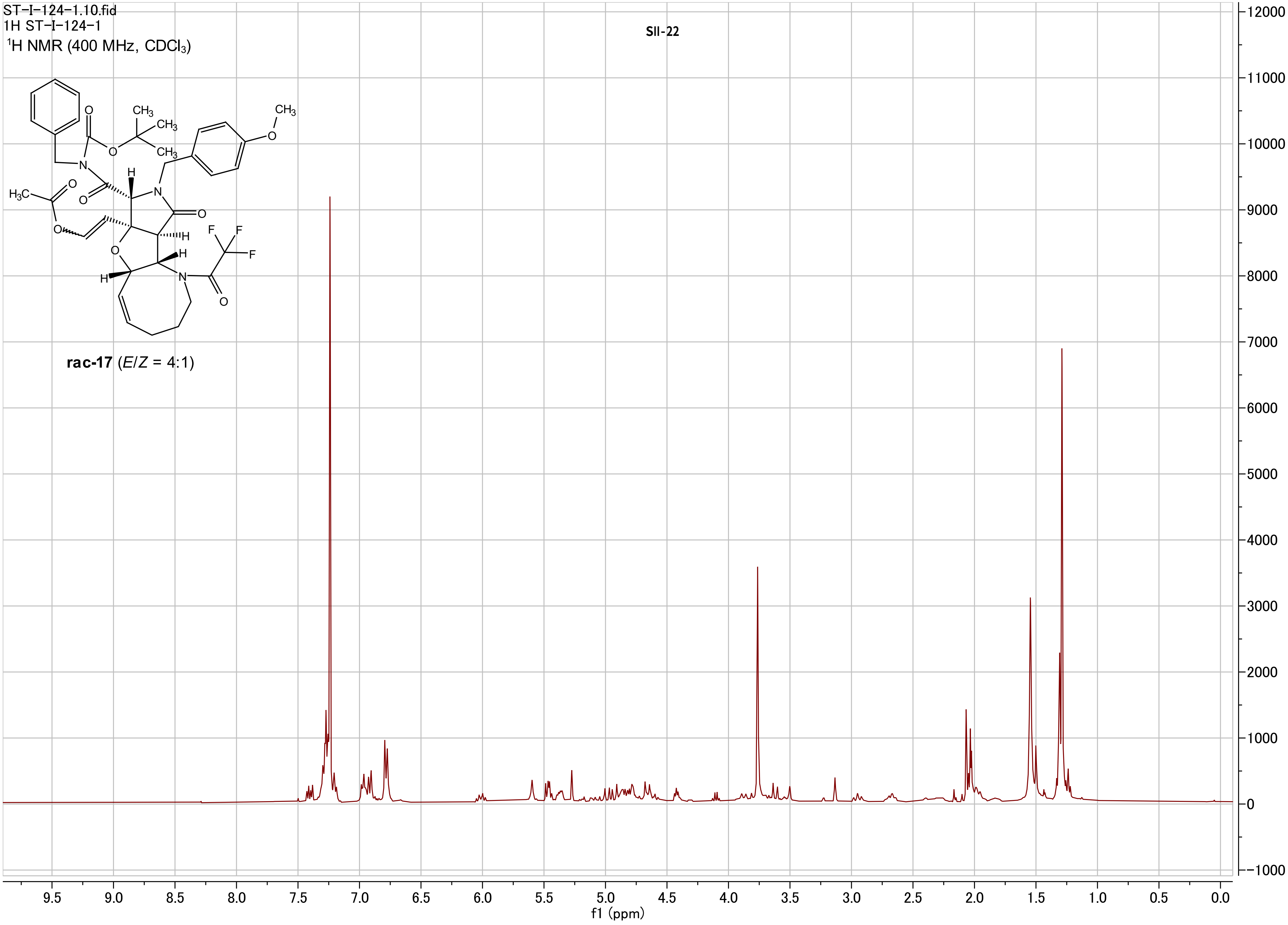
ST-I-124-1.10.fid
1H ST-I-124-1
1H NMR (400 MHz, CDCl3)
SII-22
rac-17 (E/Z = 4:1)
f1 (ppm)

ST-II-016-1.23.fid
13C ST-II-016-1
^{13}C NMR (100 MHz, $CDCl_3$)

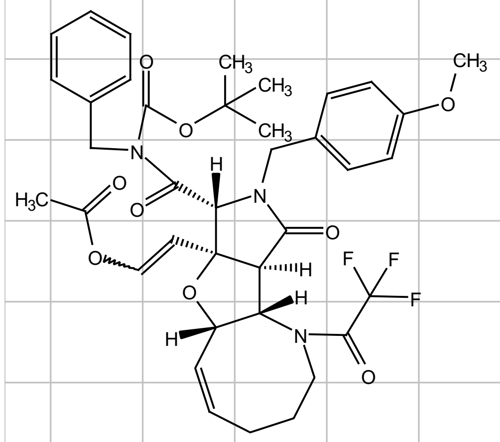

rac-17 (*E*/*Z* = 4:1)

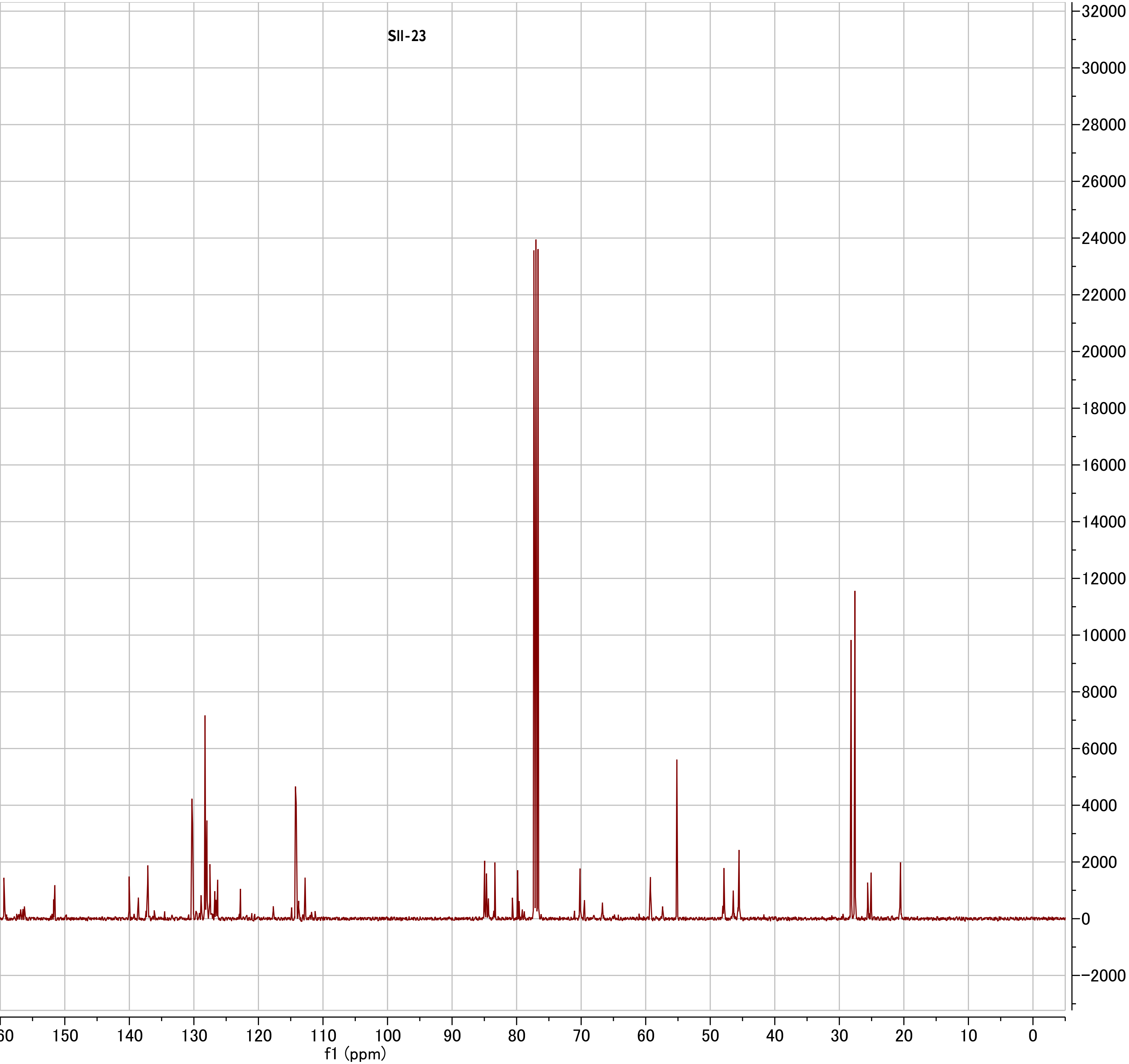

ST-II-017-3.10.fid
1H ST-II-017-3
^{1}H NMR (400 MHz, $CDCl_3$)

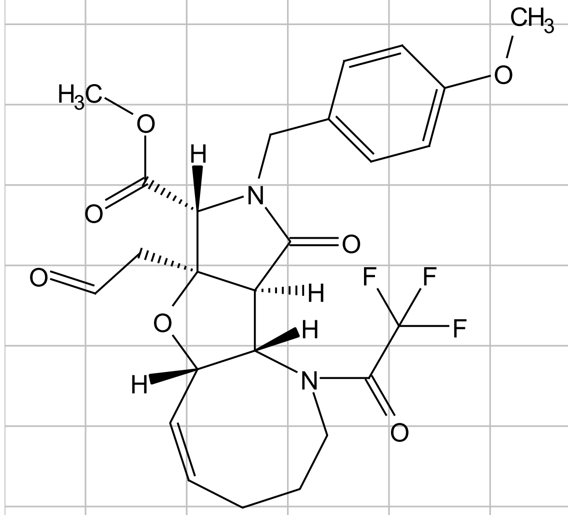

rac-18

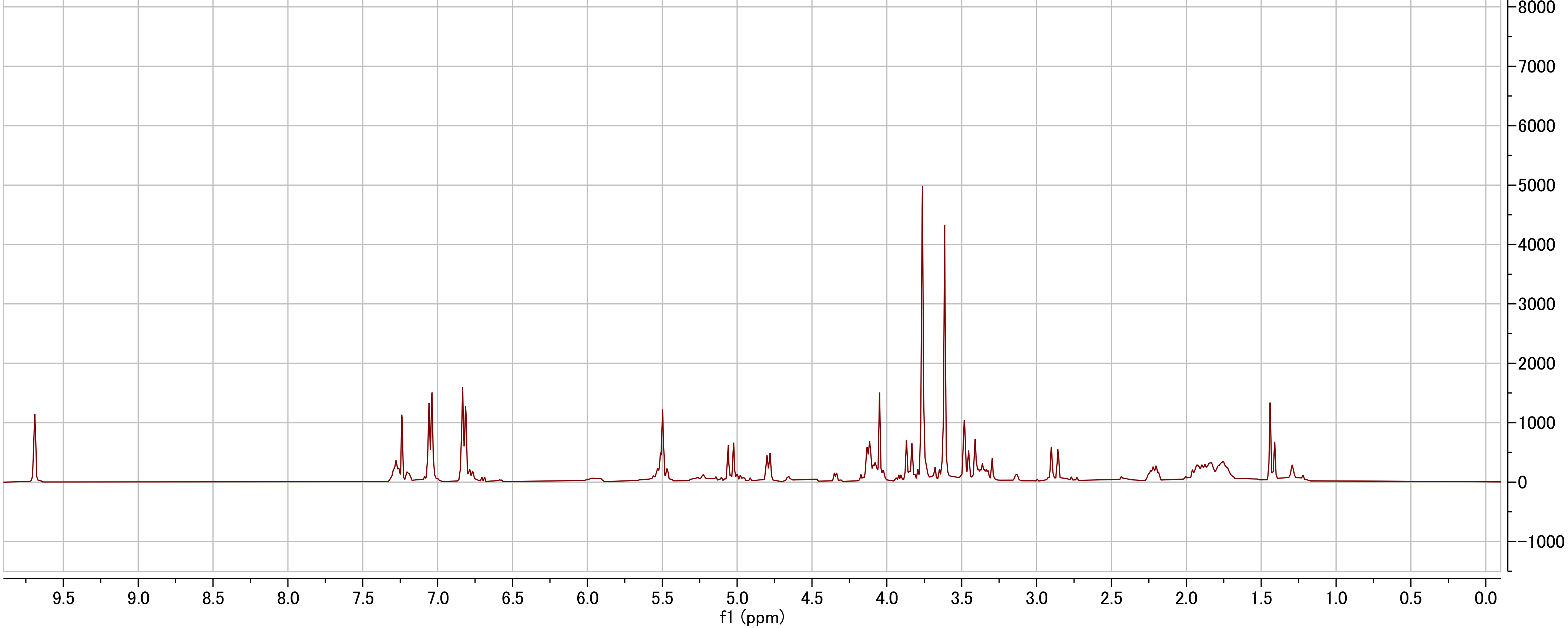

ST-II-017-3.15.fid
13C ST-II-017-3
^{13}C NMR (100 MHz, $CDCl_3$)

SII-25

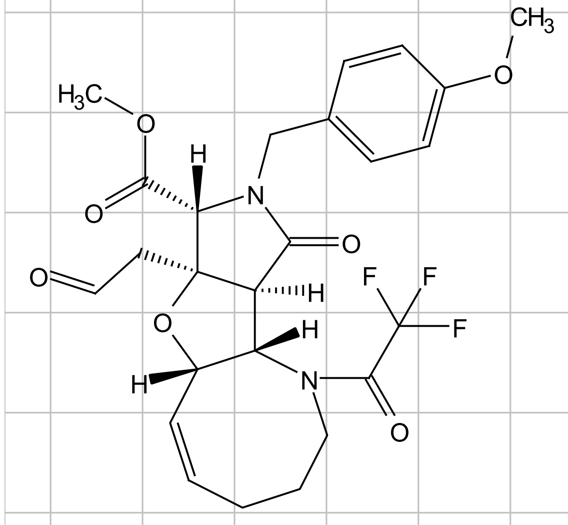

rac-18

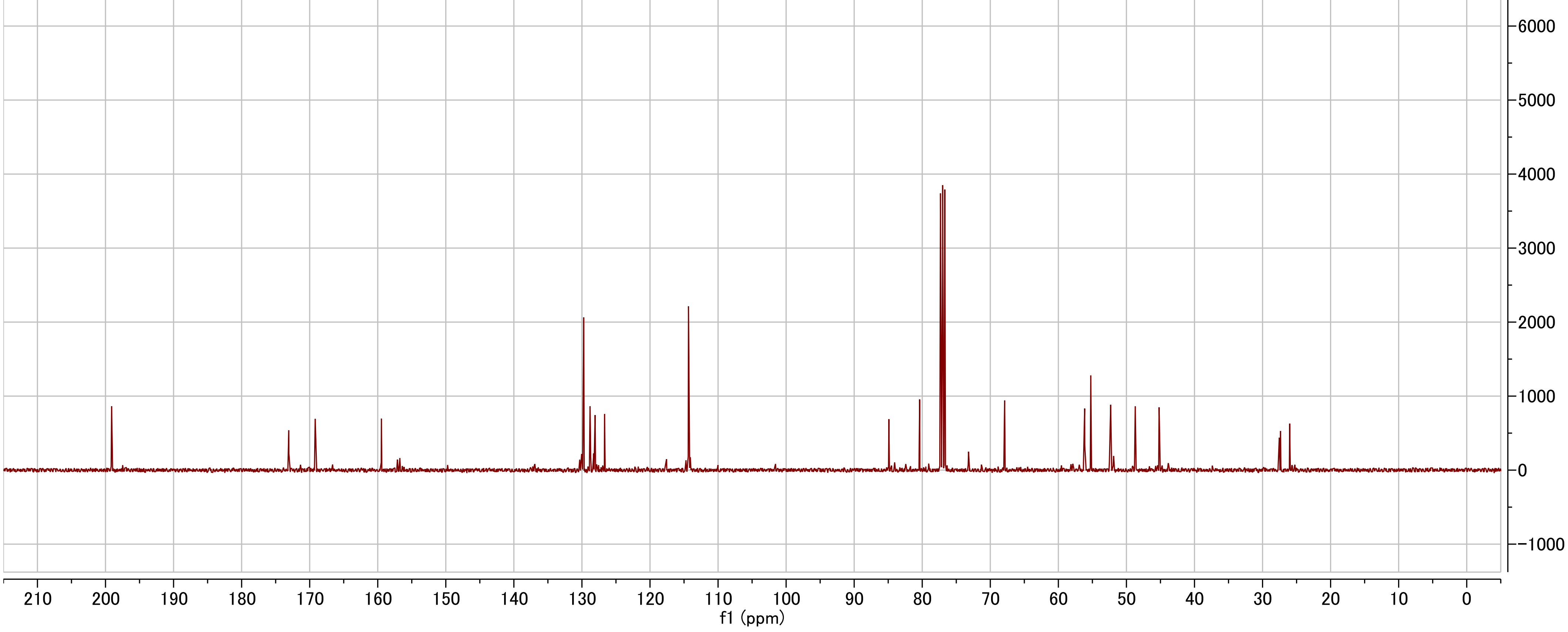

ST-II-043-1.10.fid
1H ST-II-043-1
^{1}H NMR (400 MHz, $CDCl_3$)

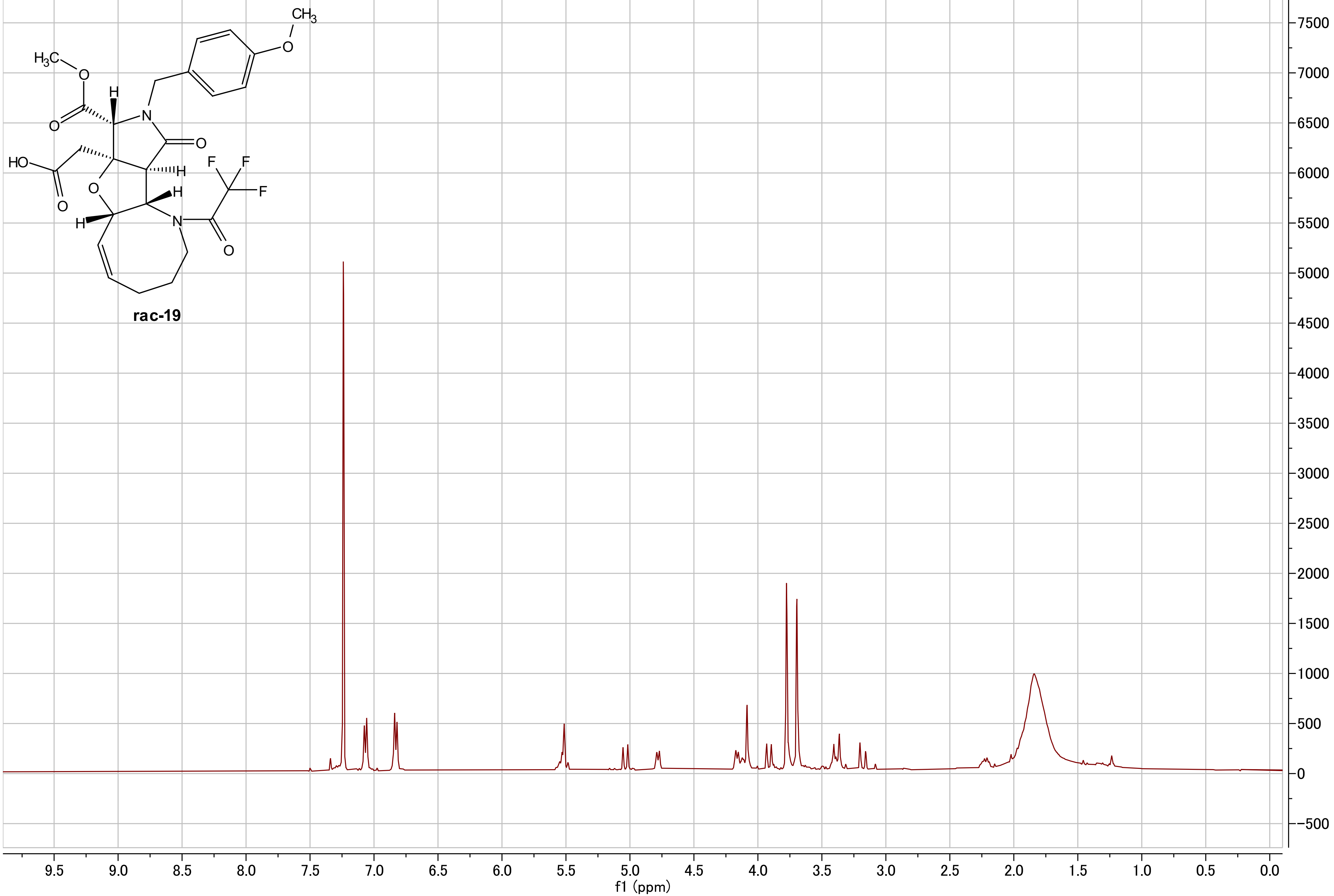

rac-19

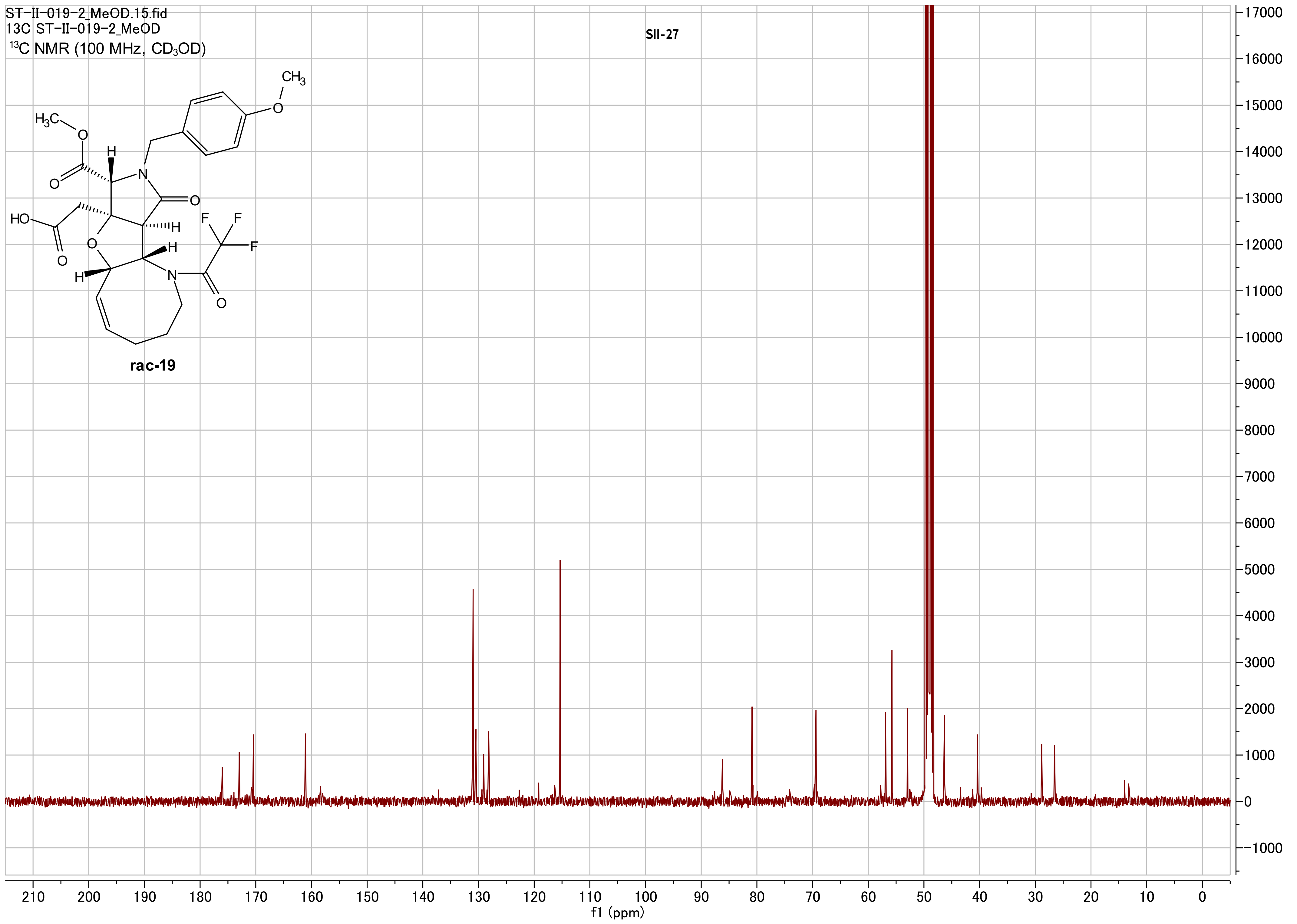
ST−II−019−2_MeOD.15.fid
13C ST−II−019−2_MeOD
13C NMR (100 MHz, CD3OD)
SII-27
rac-19
f1 (ppm)

ST-II-048-1.10.fid
1H ST-II-048-1
^{1}H NMR (400 MHz, $CDCl_3$)

SII-28

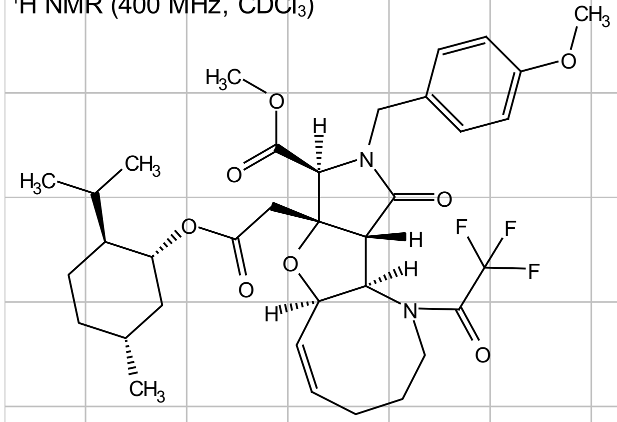

20* (2S)

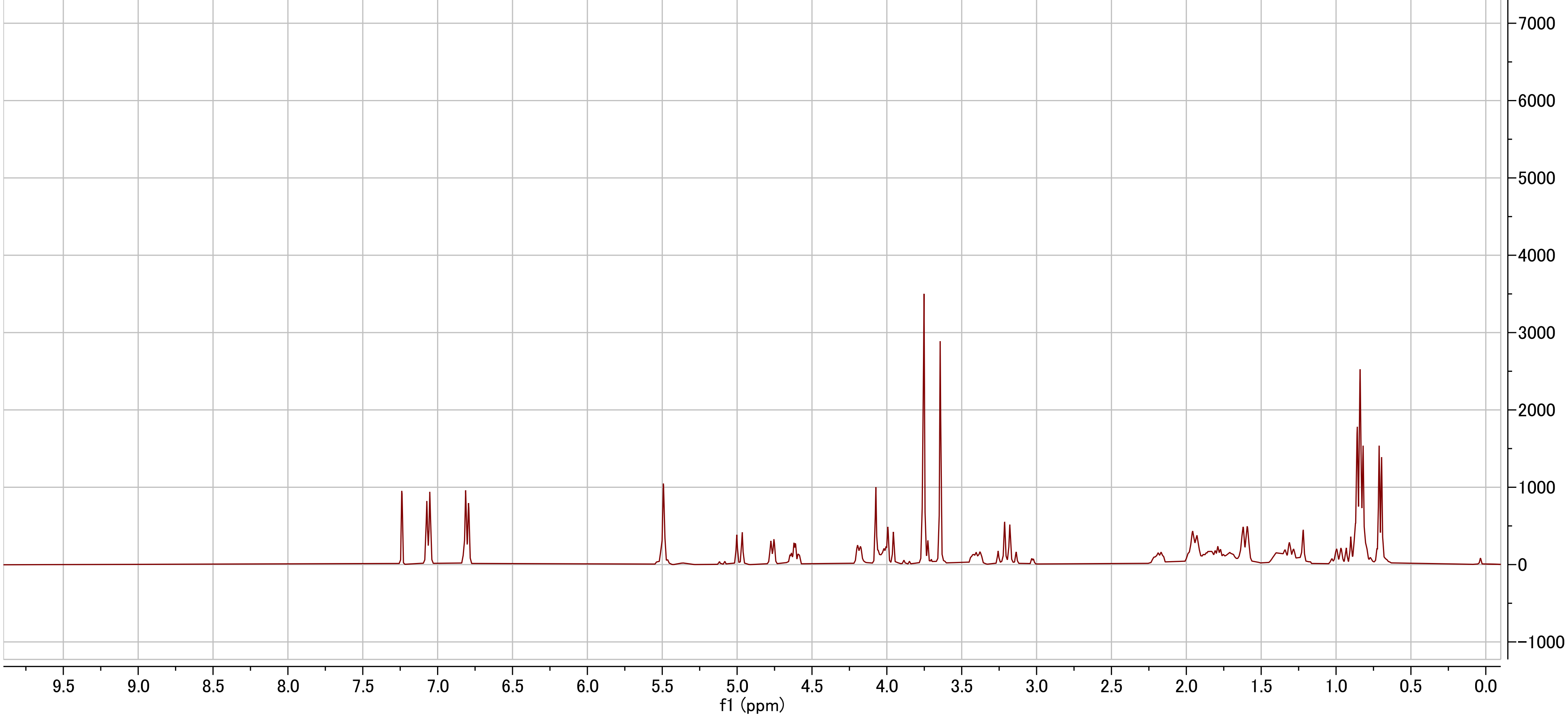

ST-II-048-1.11.fid
13C ST-II-048-1
^{13}C NMR (100 MHz, $CDCl_3$)

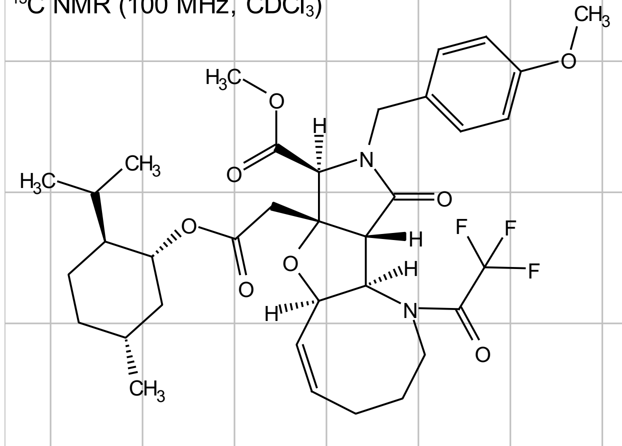

20* (2S)

SII-29

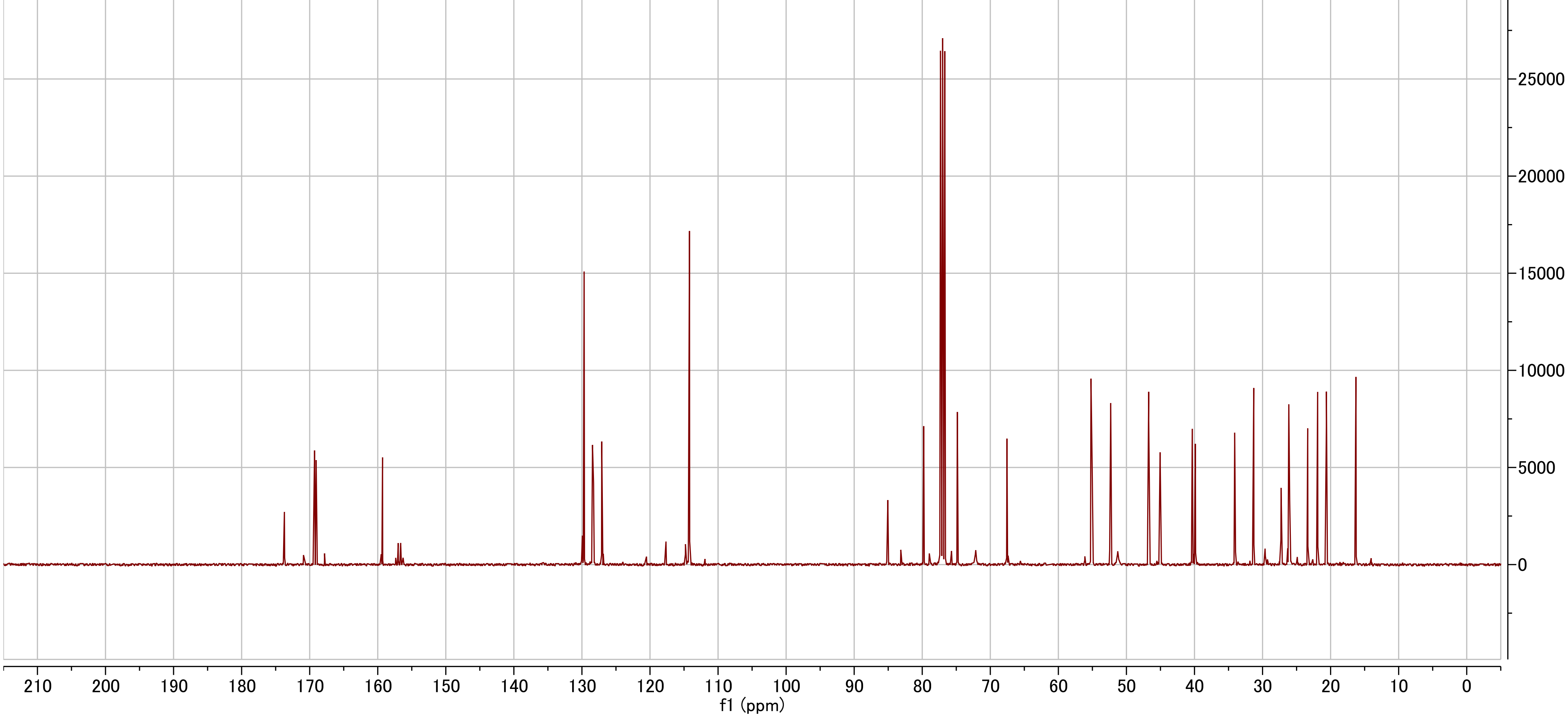

ST–II–048–2.10.fid
1H ST–II–048–2
^{1}H NMR (400 MHz, $CDCl_3$)

SII-30

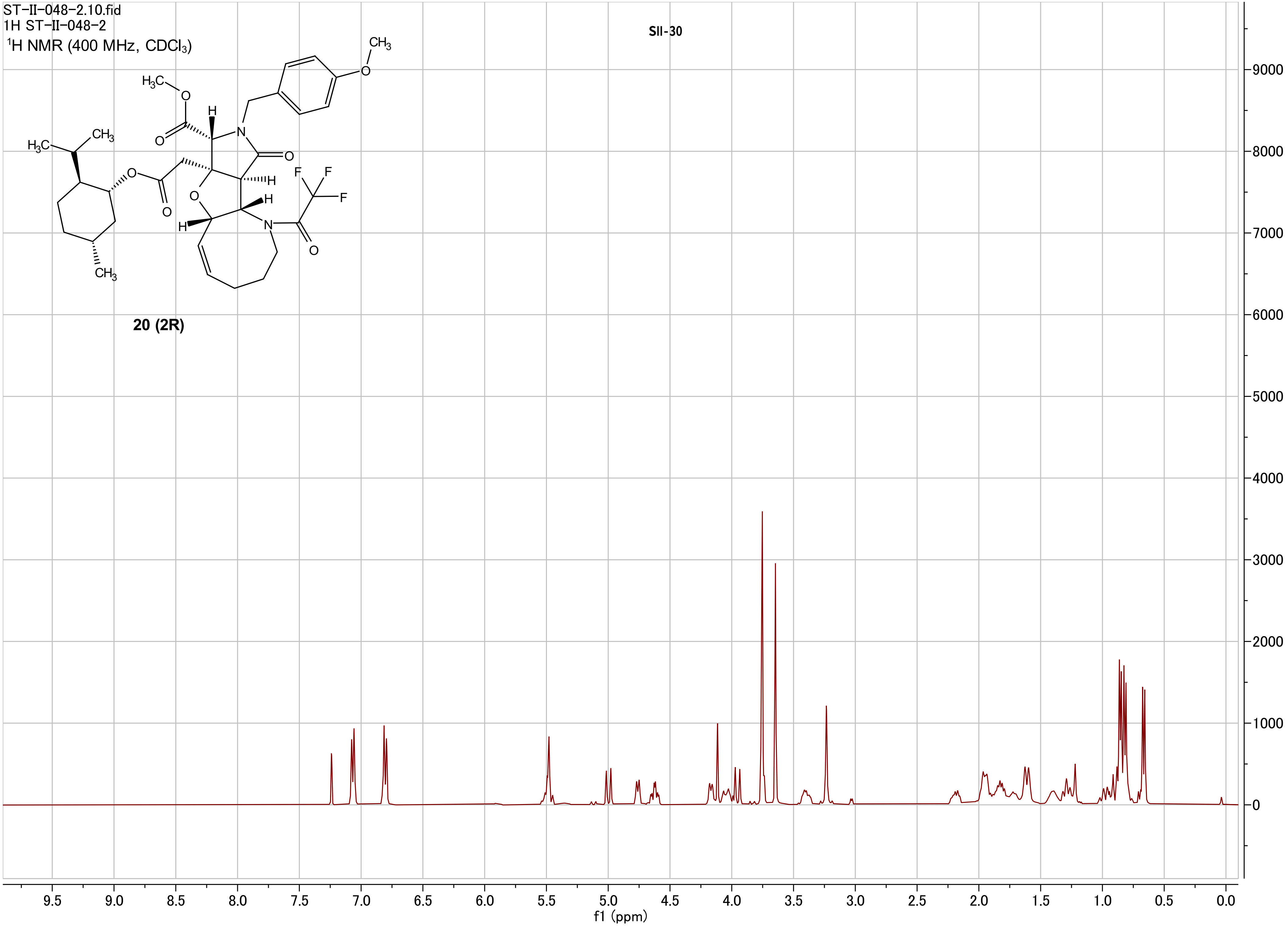

ST-II-048-2.11.fid
13C ST-II-048-2
^{13}C NMR (100 MHz, $CDCl_3$)

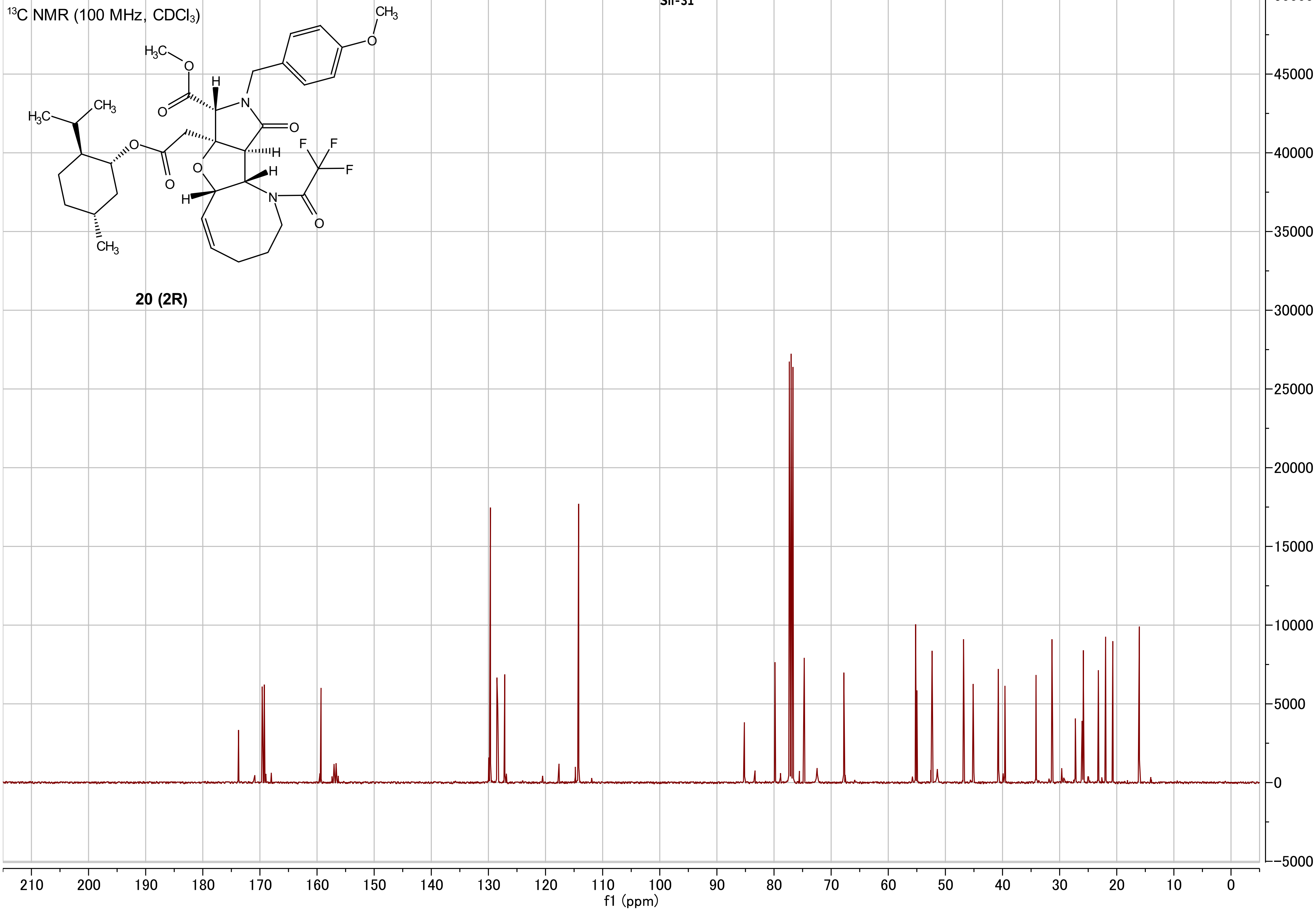

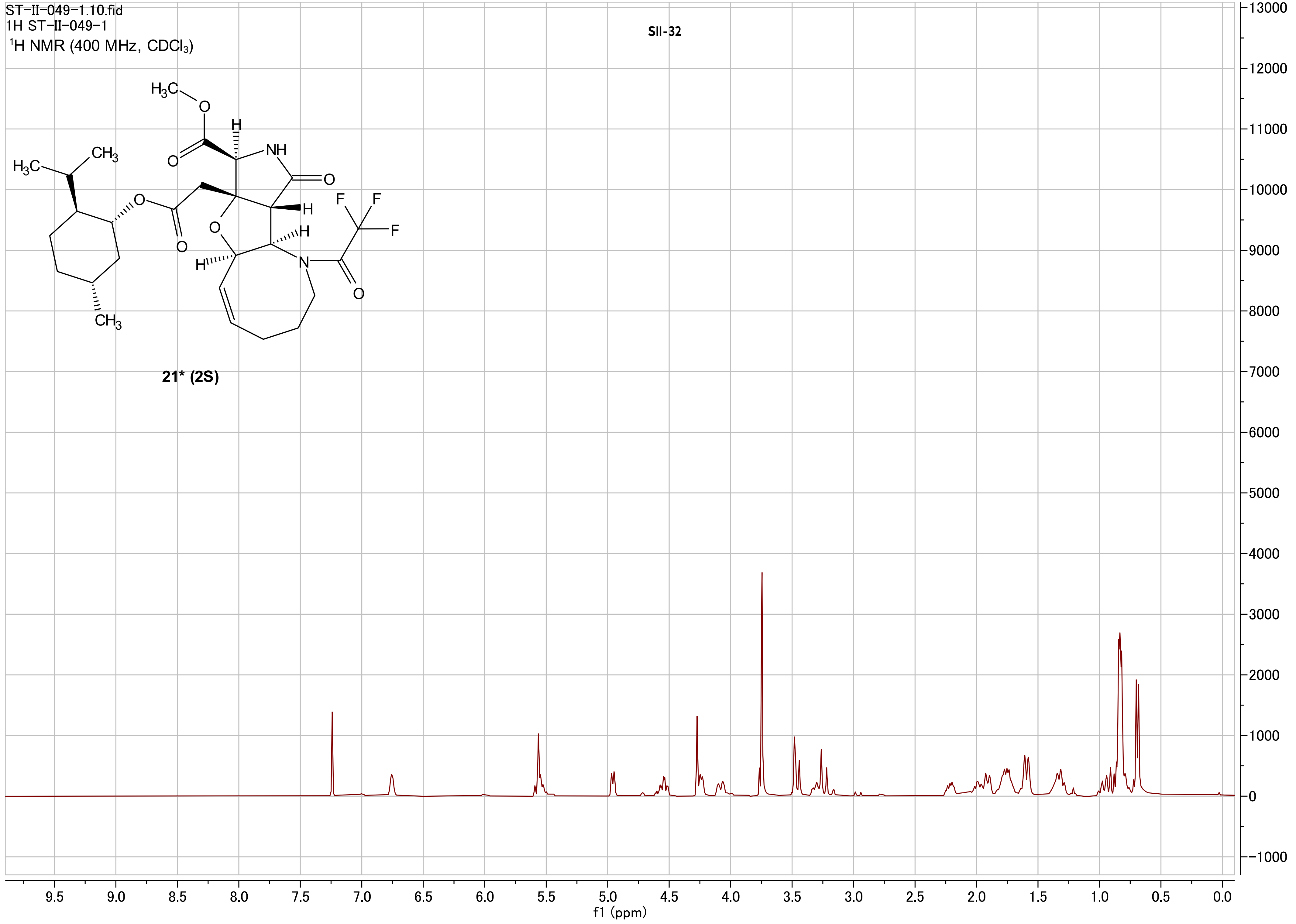
ST-II-049-1.10.fid
1H ST-II-049-1
1H NMR (400 MHz, CDCl3)
SII-32
21* (2S)
f1 (ppm)

ST-II-049-1.20.fid
13C ST-II-049-1
^{13}C NMR (100 MHz, $CDCl_3$)

SII-33

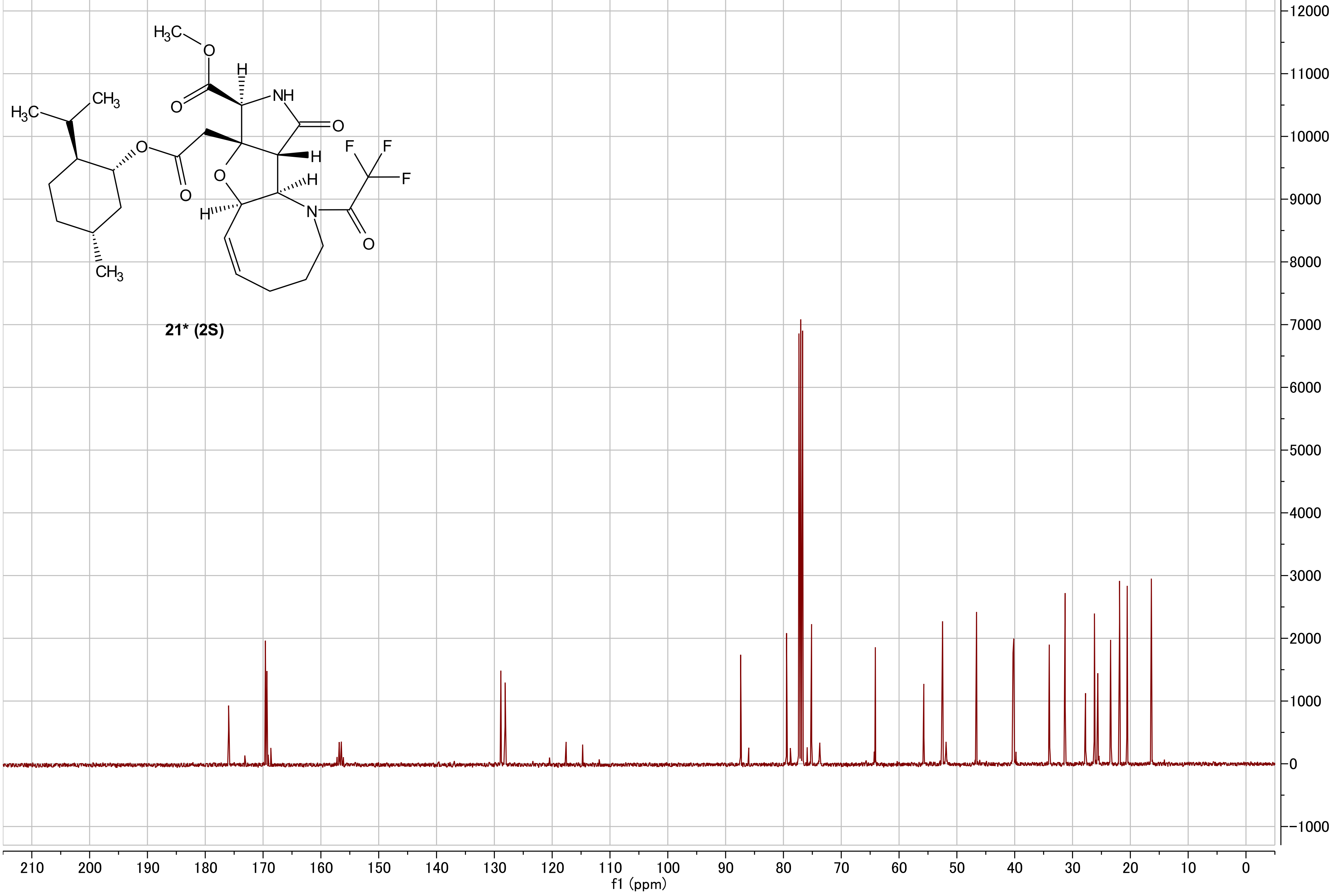

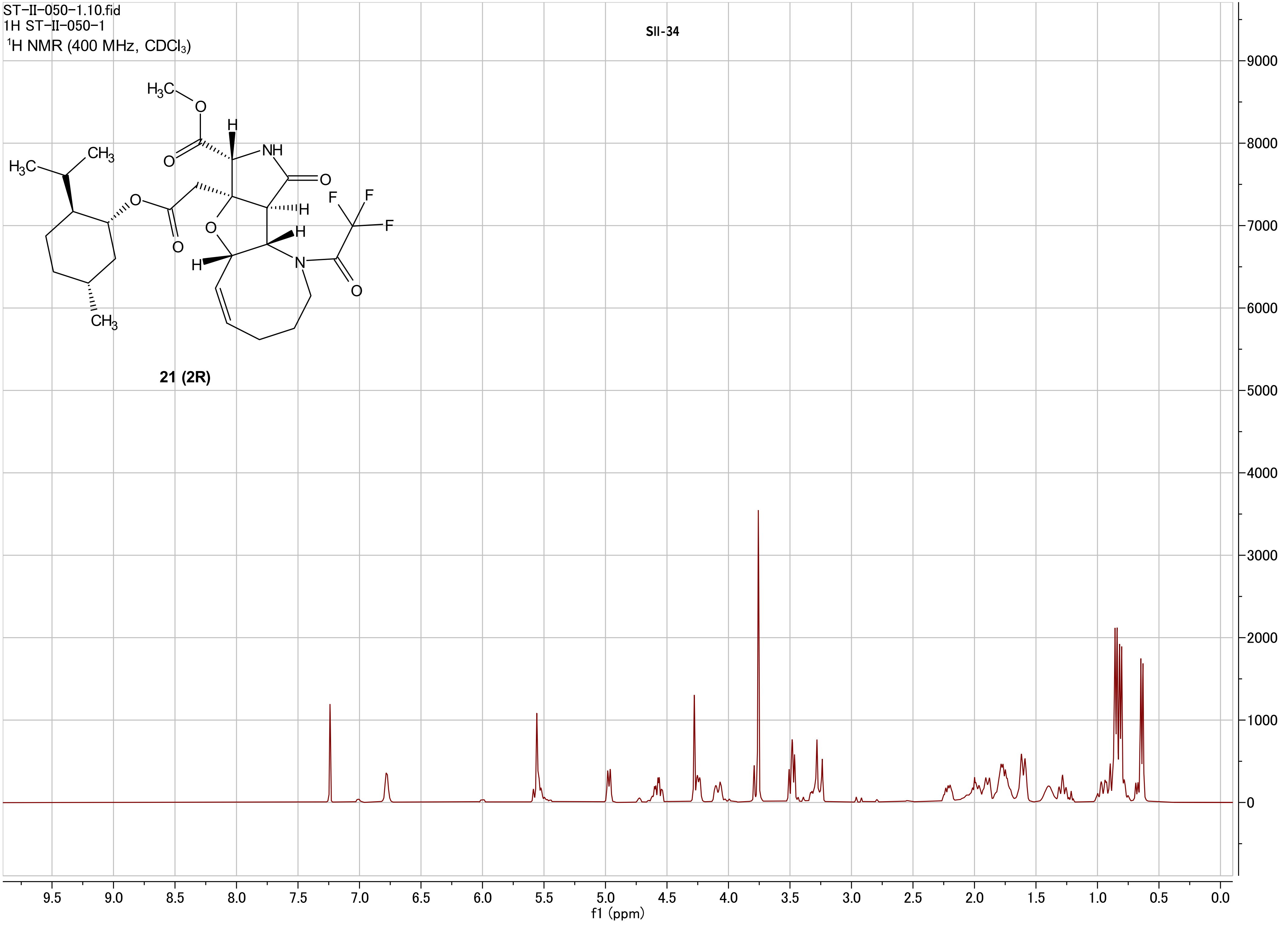
ST-II-050-1.10.fid
1H ST-II-050-1
1H NMR (400 MHz, CDCl3)
21 (2R)
f1 (ppm)

ST-II-050-1.13.fid
13C ST-II-050-1
^{13}C NMR (100 MHz, $CDCl_3$)

SII-35

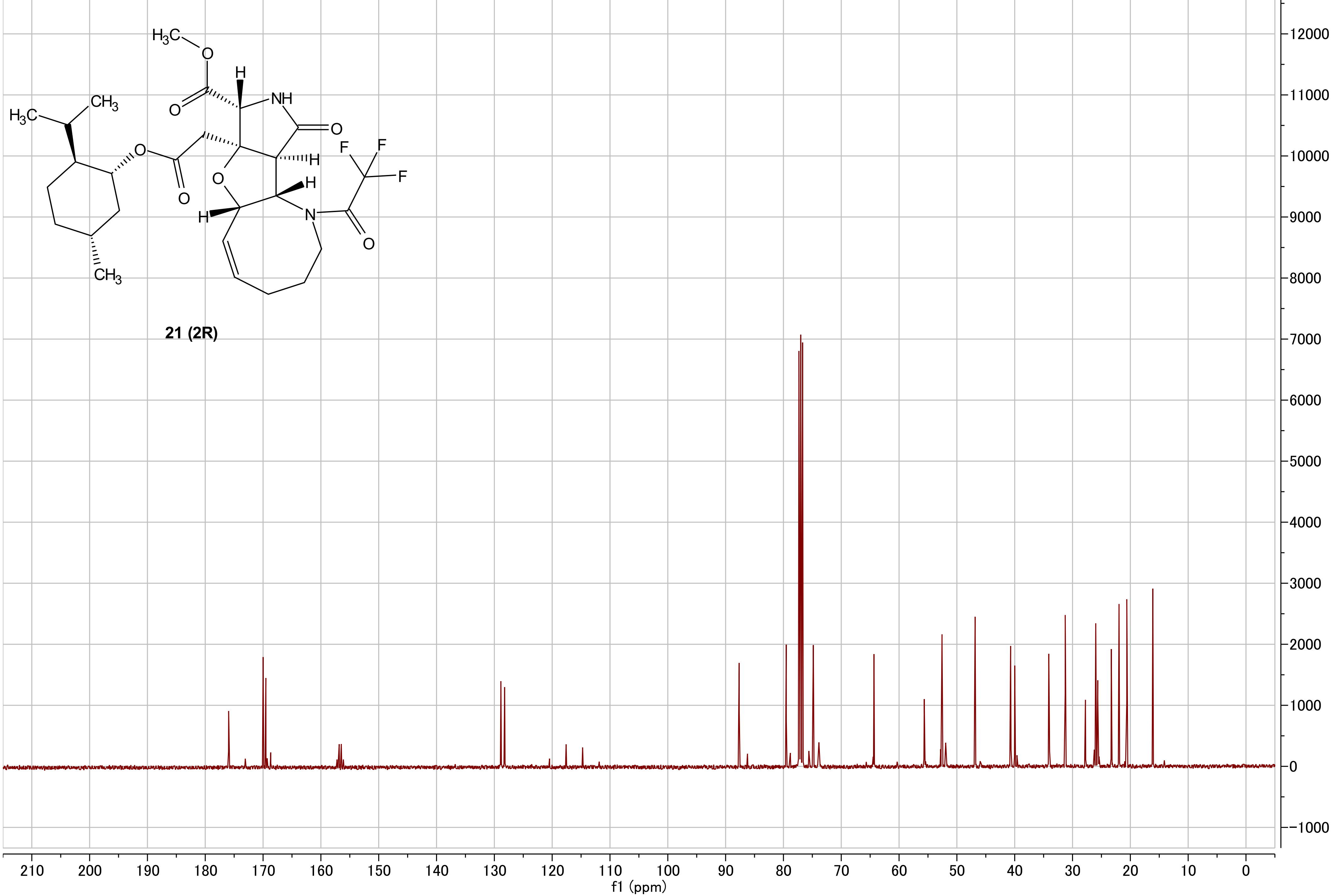

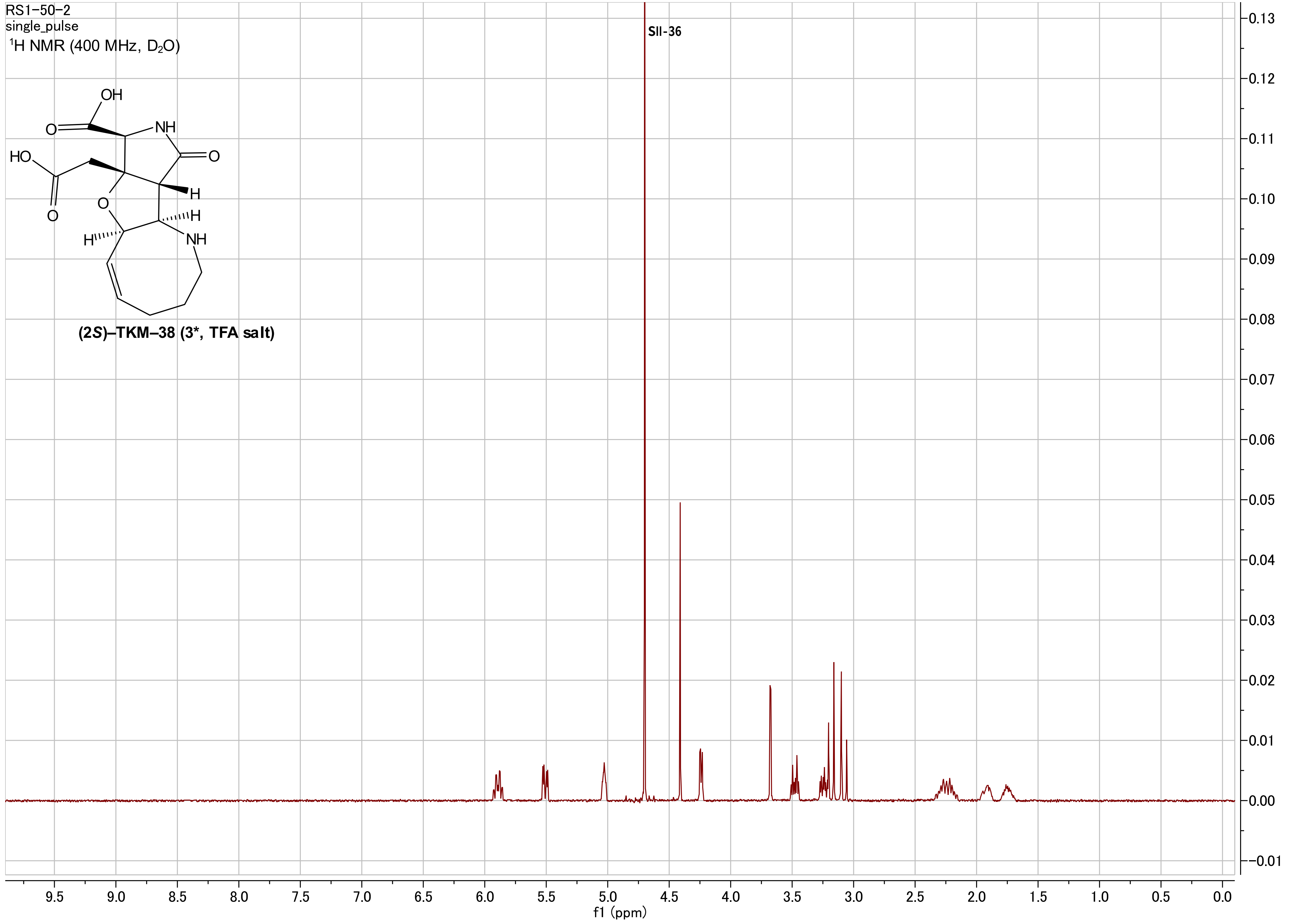
RS1-50-2
single_pulse
^{1}H NMR (400 MHz, D_2O)
OH
O
NH
HO
O
H
O
O
H
H
NH
(2S)–TKM–38 (3*, TFA salt)
SII-36
0.13
0.12
0.11
0.10
0.09
0.08
0.07
0.06
0.05
0.04
0.03
0.02
0.01
0.00
−0.01
9.5
9.0
8.5
8.0
7.5
7.0
6.5
6.0
5.5
5.0
4.5
4.0
3.5
3.0
2.5
2.0
1.5
1.0
0.5
0.0
f1 (ppm)

ST-II-052-1.11.fid
13C ST-II-052-1
^{13}C NMR (100 MHz, D_2O)

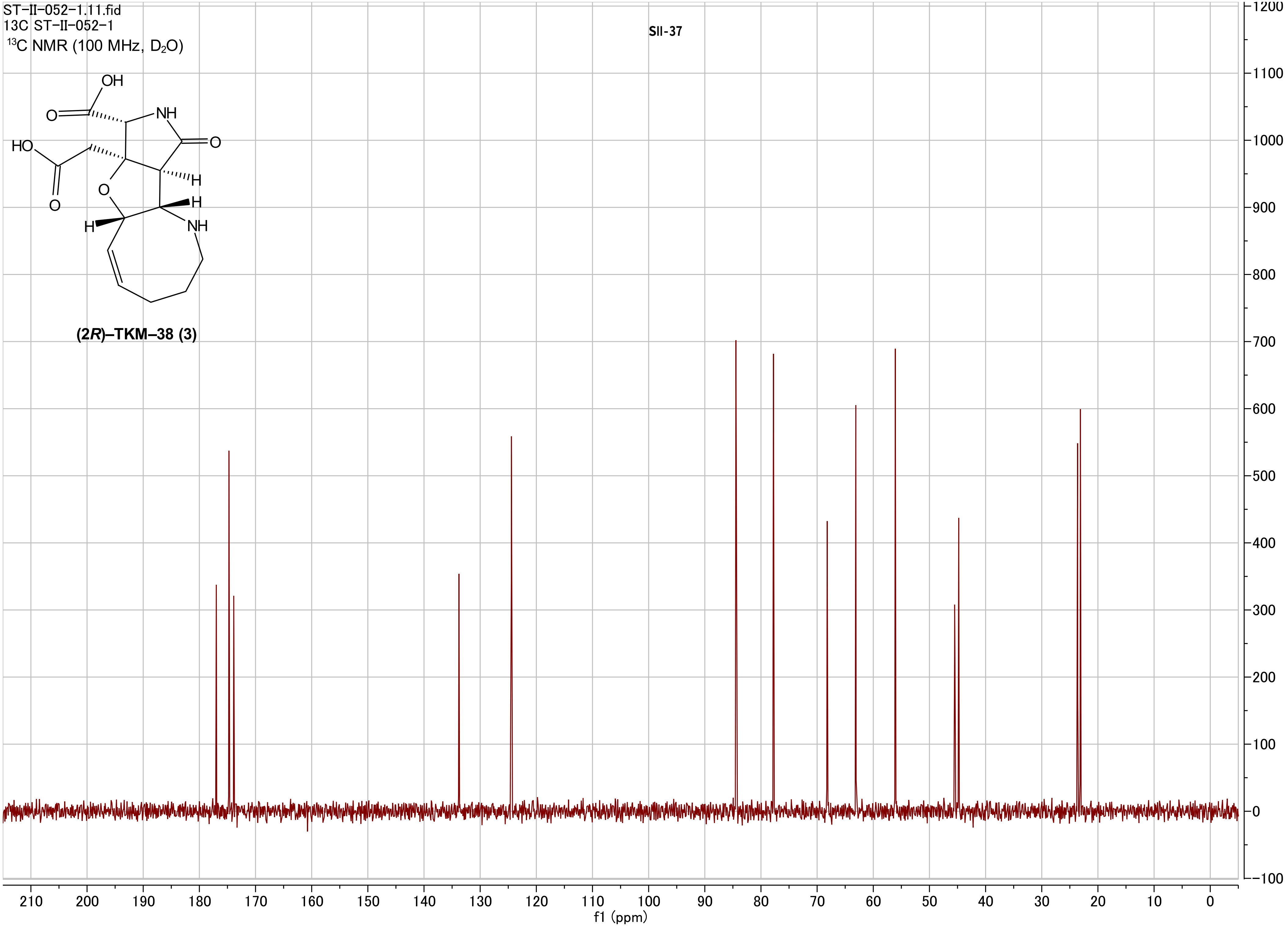

21* (2*S*)

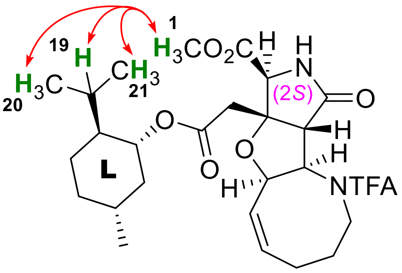

NOESY

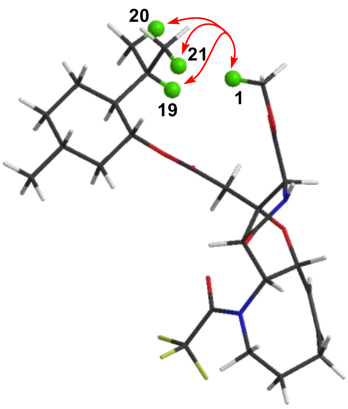

CONFLEX
population 89%

21 (2*R*)

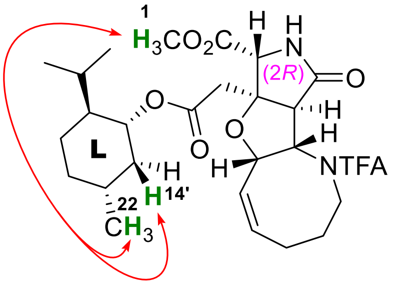

NOESY

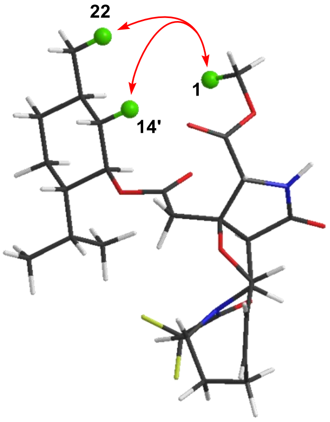

CONFLEX
population 77%

21* (2*S*)

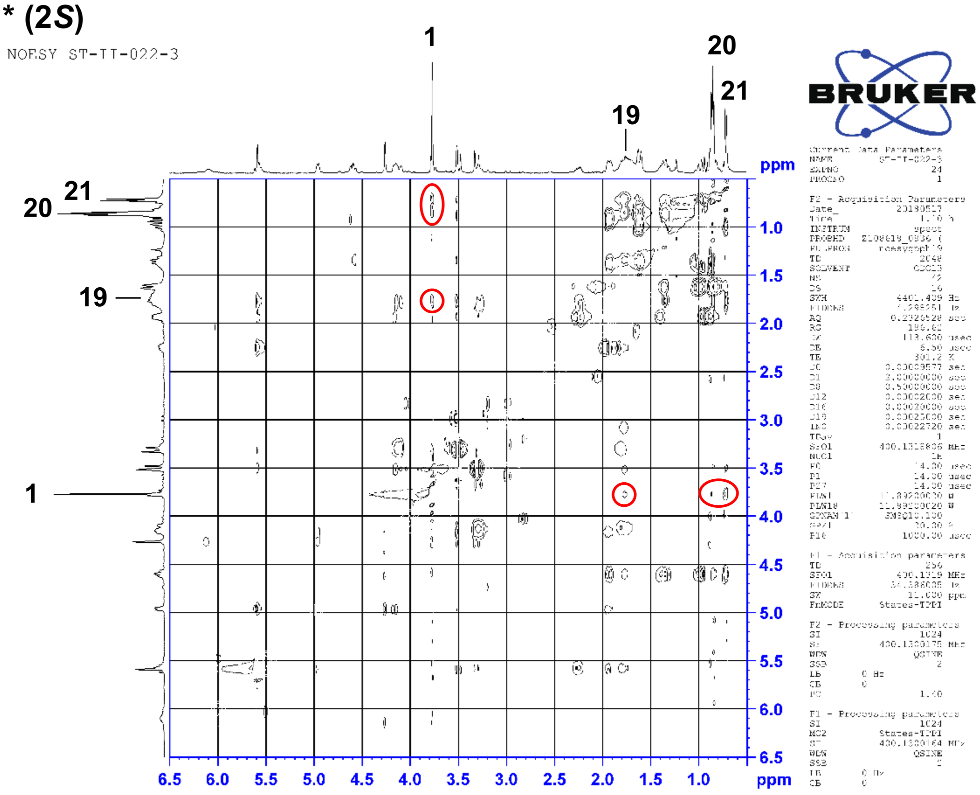
NOESY ST-TT-022-3
1
20
21
19
BRUKER
ppm
20
21
19
1
1.0
1.5
2.0
2.5
3.0
3.5
4.0
4.5
5.0
5.5
6.0
6.5
6.5 6.0 5.5 5.0 4.5 4.0 3.5 3.0 2.5 2.0 1.5 1.0 ppm

21 (2*R*)

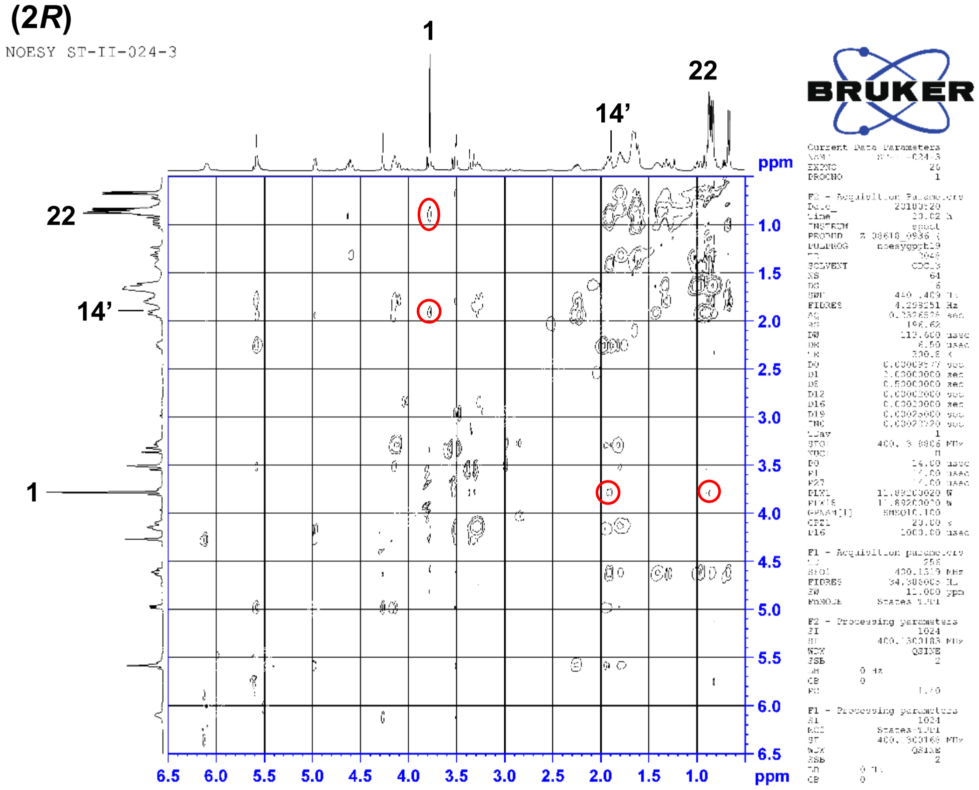
NOESY ST-TT-024-3
1
22
14'
BRUKER
ppm
22
14'
1
1.0
1.5
2.0
2.5
3.0
3.5
4.0
4.5
5.0
5.5
6.0
6.5
6.5 6.0 5.5 5.0 4.5 4.0 3.5 3.0 2.5 2.0 1.5 1.0 ppm
