# Supplementary material for: Menthyl esterification allows chiral resolution for the synthesis of artificial glutamate analogs
Source: Beilstein J Org Chem. 2021 Feb 24;17:540–50. doi: 10.3762/bjoc.17.48 (PMC7934734; doi:10.3762/bjoc.17.48)

BEILSTEIN JOURNAL OF ORGANIC CHEMISTRY

Supporting Information

for

Menthyl esterification allows chiral resolution for the synthesis of artificial glutamate analogs

Kenji Morokuma, Shuntaro Tsukamoto, Kyosuke Mori, Kei Miyako, Ryuichi Sakai, Raku Irie and Masato Oikawa

Beilstein J. Org. Chem. **2021,** *17,* 540–550. doi:10.3762/bjoc.17.48

X-ray structure of the menthyl ester 10

License and Terms: This is a supporting information file under the terms of the Creative Commons Attribution License (https://creativecommons.org/licenses/by/4.0). Please note that the reuse, redistribution and reproduction in particular requires that the author(s) and source are credited and that individual graphics may be subject to special legal provisions.
The license is subject to the *Beilstein Journal of Organic Chemistry* terms and conditions: (https://www.beilstein-journals.org/bjoc/terms)

Contents:

X-ray structure for menthyl ester **10** **SIII-2 ~ SIII-6**

Structure for menthyl ester 10:

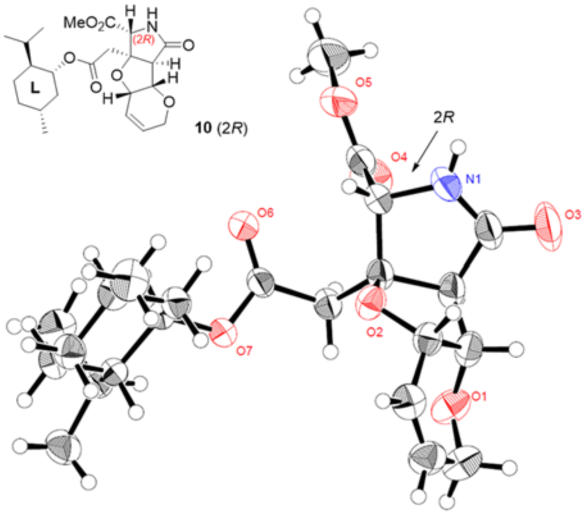

CCDC accession number: 2030829

Summary for 20200831_AA70052-002

Formula: C23 H33 N1 O7

******* Unit Cell Parameters *******

a:	6.49242(16)
b:	7.62140(18)
c:	45.6289(11)
alpha:	90.000
beta:	90.000
gamma:	90.000
volume:	2257.78(9)

********* Model Refinement *********

R1 factor[I>2.0sigma(I)]:	0.0329
R factor[all data]:	0.0361
wR factor[all data]:	0.0889
goodness of fit:	1.066
# of observations:	4090
# of variables:	284
refl/para ratio:	14.4
maximum shift/error:	0.01
Refinement program:	SHELXL 2018/3
Refinement mode:	Single
Flack Parameter:	-0.01(5)

****** Space Group Information *****

symbol:	P212121
number:	19
centricity:	acentric
Z value:	4
formula weight:	435.52
calculated density:	1.281
mu (cm-1):	7.799
crystal system:	orthorhombic
laue group:	mmm
lattice type:	P

****** Reflection Corrections ******

absorption applied:	Yes
abs. type:	SYM
abs. range:	0.815-1.000
decay applied:	No
decay (%):	0.00
redundants averaged:	Yes

******* Reflection Processing ******

total # processed:	24175
total # unique:	4090
R merge (%):	3.03
Wilson B:	3.70

***** Experimental Information ****

radiation:	Cu
wavelength:	1.54187
max. 2theta:	136.4
sin(theta)/lambda:	0.6021
temperature (C):	-30.0

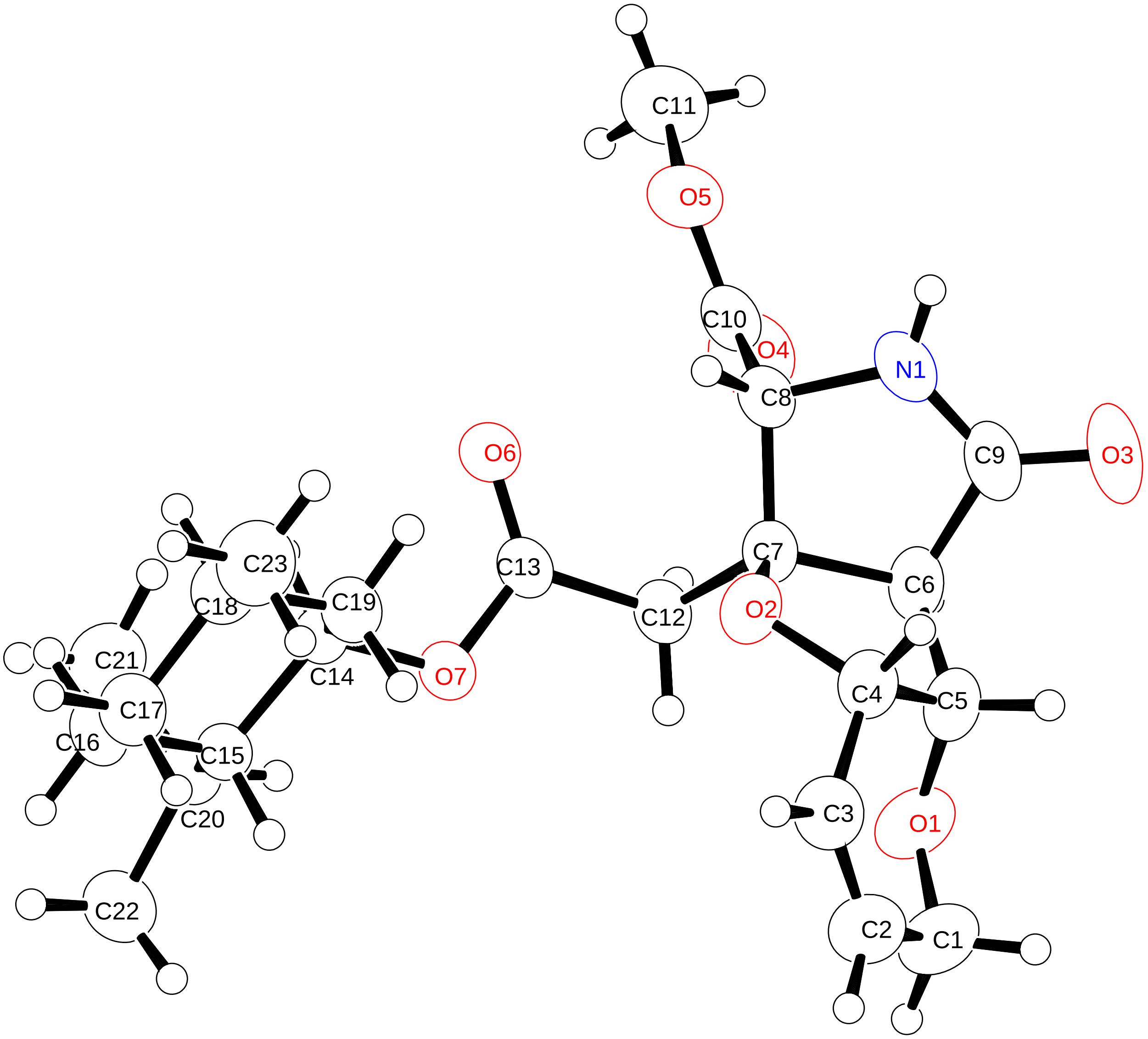
C11
O5
C10
O4
N1
C8
O6
C9
O3
C23
C7
C13
C19
C6
C18
C12
O2
C21
C14
O7
C4
C5
C17
C16
C15
C3
O1
C20
C22
C2
C1

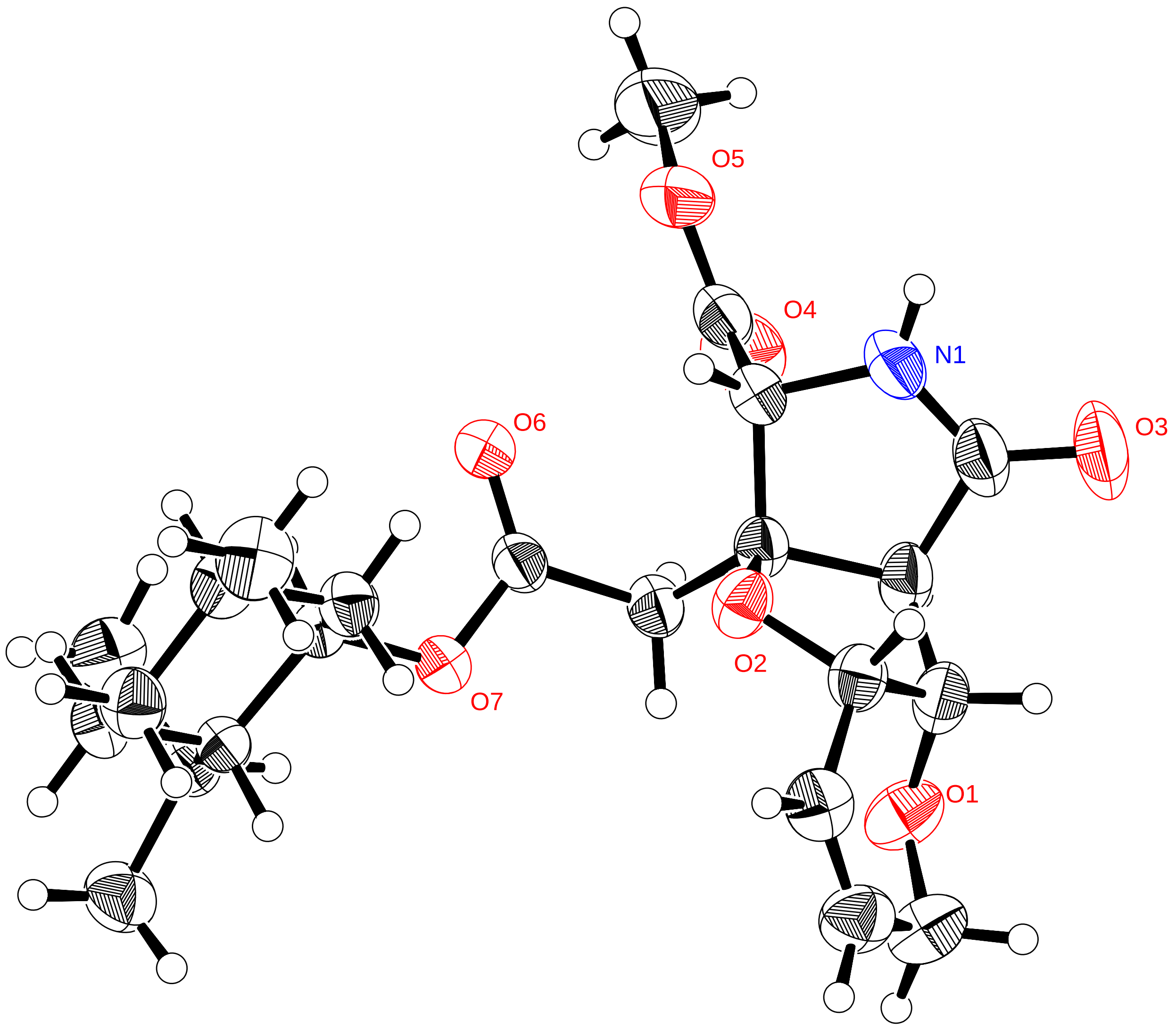
O5
O4
N1
O6
O3
O2
O7
O1

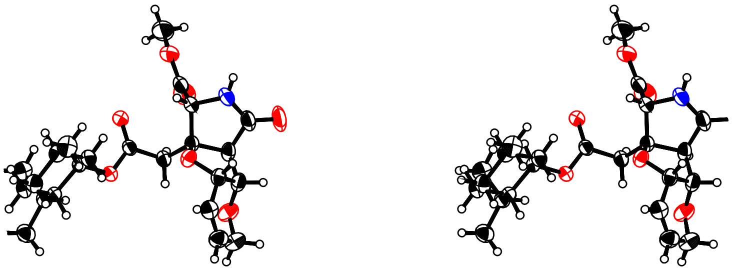

Supplement: File 3 — X-ray structure of the menthyl ester 10. [file Beilstein_J_Org_Chem-17-540-s003.pdf]
