# Supplementary material for: Menthyl esterification allows chiral resolution for the synthesis of artificial glutamate analogs
Source: Beilstein J Org Chem. 2021 Feb 24;17:540–50. doi: 10.3762/bjoc.17.48 (PMC7934734; doi:10.3762/bjoc.17.48)
Supplement: File 5 — Stereo diagrams for 10, 21*, and 21 as well as superimposed structures of the stable conformers of 10*. [file Beilstein_J_Org_Chem-17-540-s005.pdf]

BEILSTEIN JOURNAL OF ORGANIC CHEMISTRY

Supporting Information

for

Menthyl esterification allows chiral resolution for the synthesis of artificial glutamate analogs

Kenji Morokuma, Shuntaro Tsukamoto, Kyosuke Mori, Kei Miyako, Ryuichi Sakai, Raku Irie and Masato Oikawa

Beilstein J. Org. Chem. **2021,** *17,* 540–550. doi:10.3762/bjoc.17.48

Stereo diagrams for 10, 21*, and 21 as well as superimposed structures of the stable conformers of 10*

License and Terms: This is a supporting information file under the terms of the Creative Commons Attribution License (https://creativecommons.org/licenses/by/4.0). Please note that the reuse, redistribution and reproduction in particular requires that the author(s) and source are credited and that individual graphics may be subject to special legal provisions.
The license is subject to the *Beilstein Journal of Organic Chemistry* terms and conditions: (https://www.beilstein-journals.org/bjoc/terms)

Contents:

Stereo diagram of the most stable conformer in Figure 3, generated by CONFLEX (MMFF94S) for **10** SV-2

Stereo diagram of the most stable conformer in Figure 7, generated by CONFLEX (MMFF94S) for **21*** SV-3

Stereo diagram of the most stable conformer in Figure 8, generated by CONFLEX (MMFF94S) for **21** SV-4

Superimposed structures of the top 7 stable conformers (97.5% total population) generated by CONFLEX (MMFF94S) for **10*** (2*S*) show the single bonds between the heterotricycle and the menthyl ring are freely rotating SV-5

Stereo diagram of the most stable conformer generated by CONFLEX (MMFF94S) for **10** (parallel viewing)

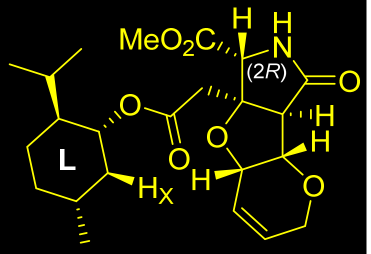

(2*R*)–isomer

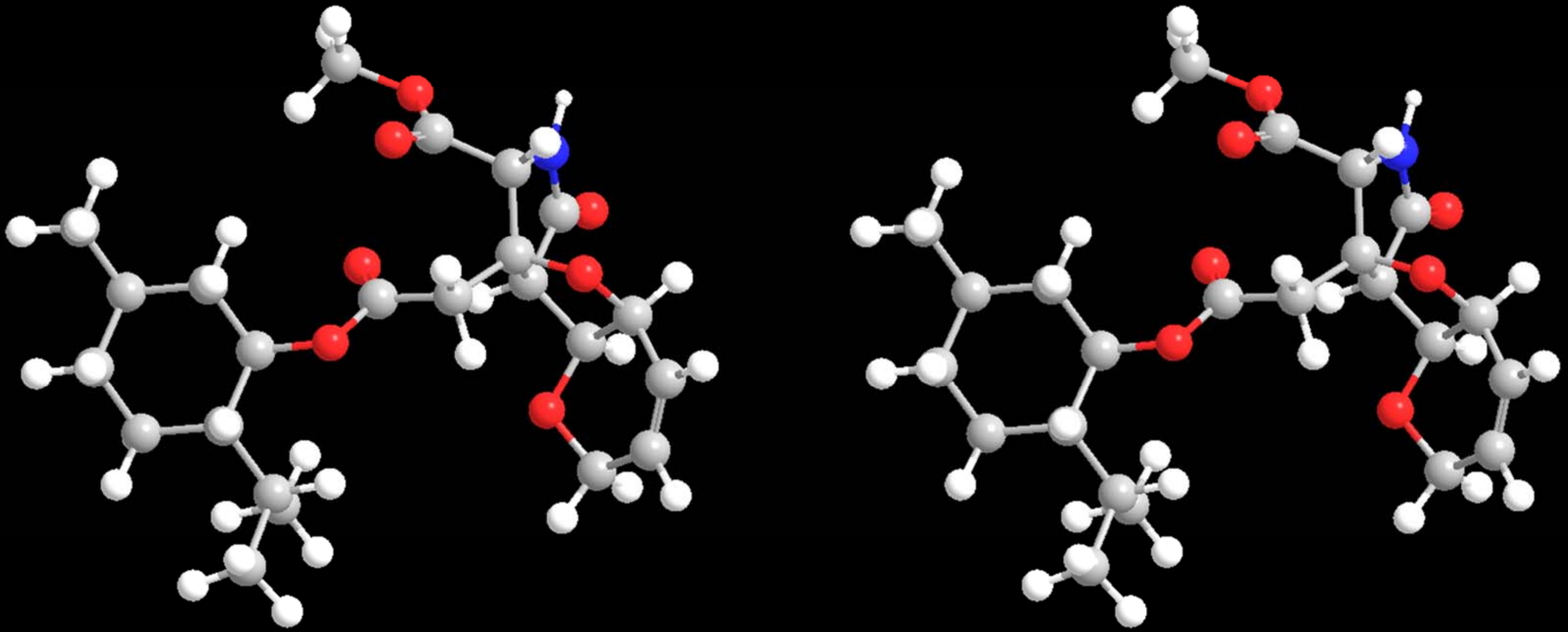

Stereo diagram of the most stable conformer generated by CONFLEX (MMFF94S) for **21*** (2*S*) (parallel viewing)

NOESY observed for **21***

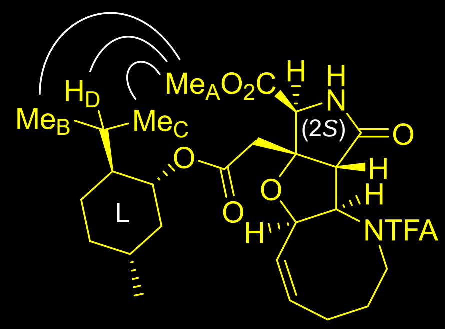

(2*S*)–isomer

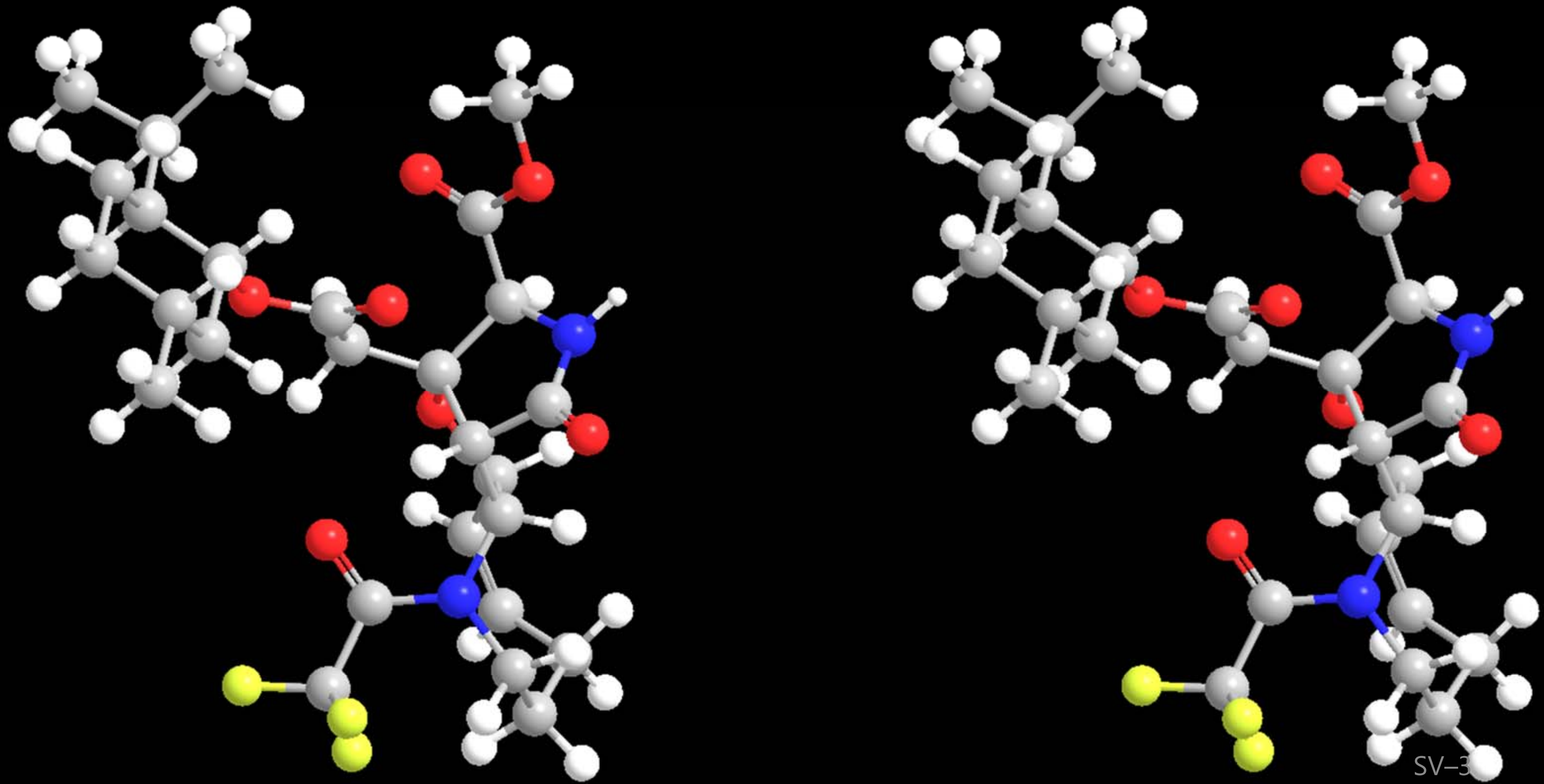

Stereo diagram of the most stable conformer generated by CONFLEX (MMFF94S) for **21** (2*R*) (parallel viewing)

NOESY
observed for **21**

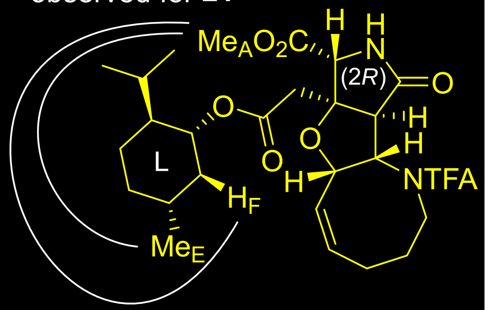

(2*R*)–isomer

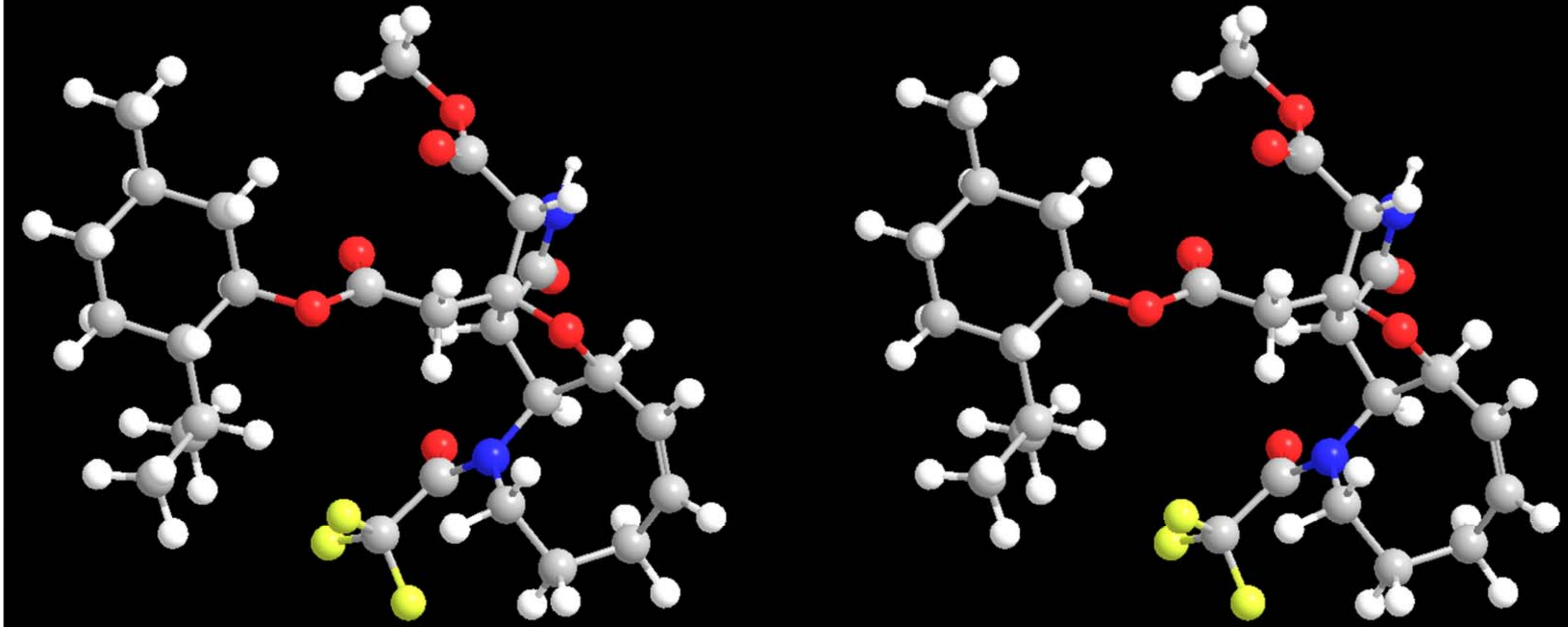

Superimposed structures of the top 7 stable conformers (97.5% total population) generated by CONFLEX (MMFF94S) for **10*** (2*S*) show the single bonds between the heterotricycle and the menthyl ring are freely rotating

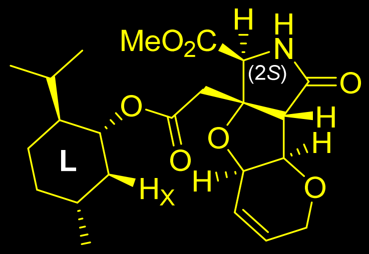

(2*S*)–isomer

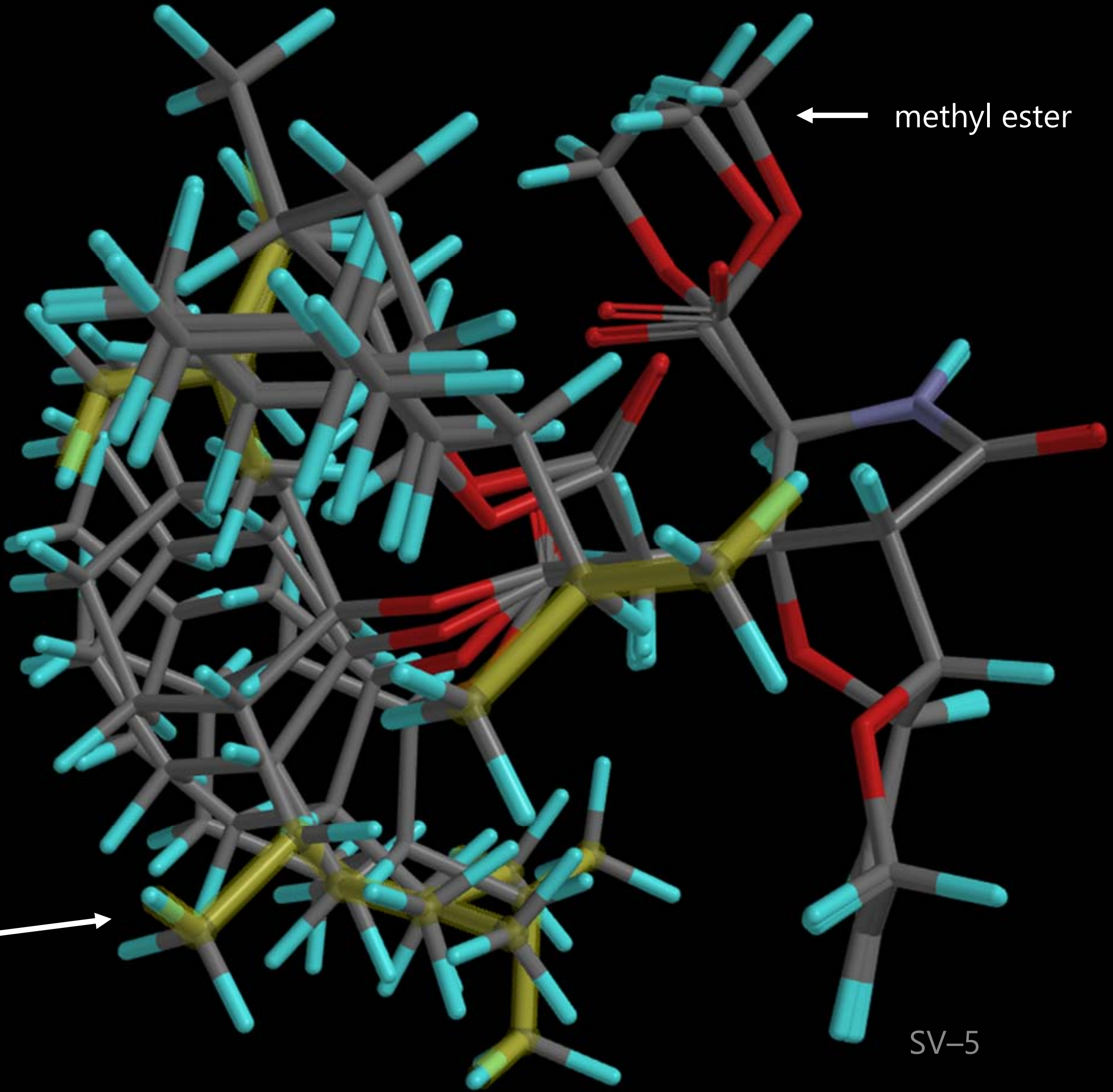

methyl ester

isopropyl C–C bond indicated by yellow line
