# Supplementary material for: Menthyl esterification allows chiral resolution for the synthesis of artificial glutamate analogs
Source: Beilstein J Org Chem. 2021 Feb 24;17:540–50. doi: 10.3762/bjoc.17.48 (PMC7934734; doi:10.3762/bjoc.17.48)

BEILSTEIN JOURNAL OF ORGANIC CHEMISTRY

Supporting Information

for

Menthyl esterification allows chiral resolution for the synthesis of artificial glutamate analogs

Kenji Morokuma, Shuntaro Tsukamoto, Kyosuke Mori, Kei Miyako, Ryuichi Sakai, Raku Irie and Masato Oikawa

Beilstein J. Org. Chem. **2021,** *17,* 540–550. doi:10.3762/bjoc.17.48

Assignments and intensities of all NOESY crosspeaks observed for 10*, 10, 21*, and 21

License and Terms: This is a supporting information file under the terms of the Creative Commons Attribution License (https://creativecommons.org/licenses/by/4.0). Please note that the reuse, redistribution and reproduction in particular requires that the author(s) and source are credited and that individual graphics may be subject to special legal provisions.
The license is subject to the *Beilstein Journal of Organic Chemistry* terms and conditions: (https://www.beilstein-journals.org/bjoc/terms)

Contents:

Assignments and intensities of all NOESY cross peaks observed for **10*** (2*S*) SVI-2

Assignments and intensities of all NOESY cross peaks observed for **10** (2*R*) SVI-6

Assignments and intensities of all NOESY cross peaks observed for **21*** (2*S*) SV-11

Assignments and intensities of all NOESY cross peaks observed for **21** (2*R*) SV-13

Assignments and intensities of all NOESY cross peaks observed for **10*** (2*S*)

^{1}H NMR for **10*** (2*S*) (400 MHz, $CDCl_3$)

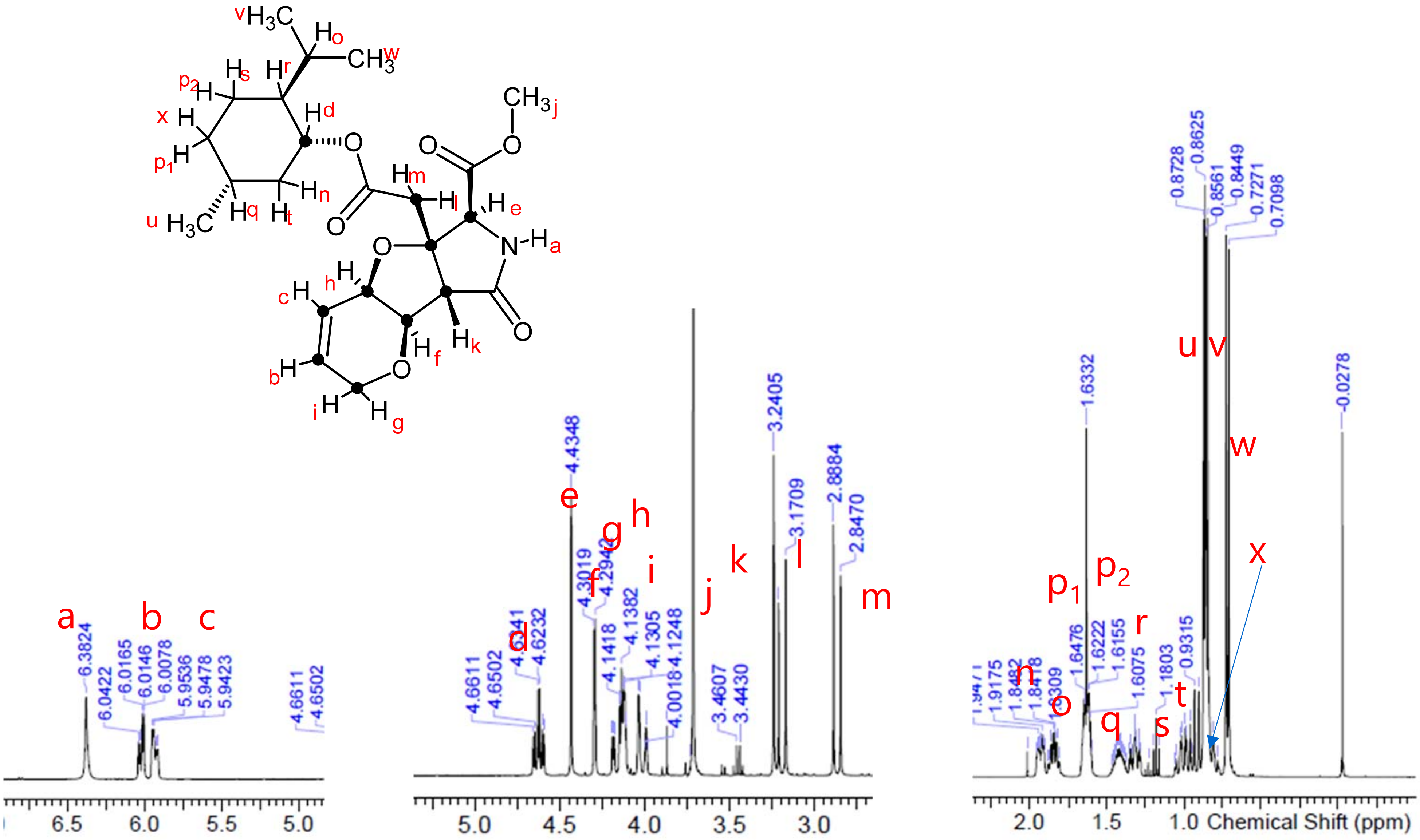

NOESY analysis of **10*** (2*S*) (400 MHz, $CDCl_3$) #1/2

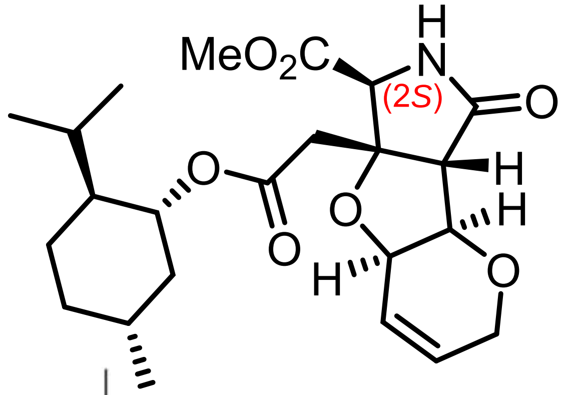

10* (2*S*)

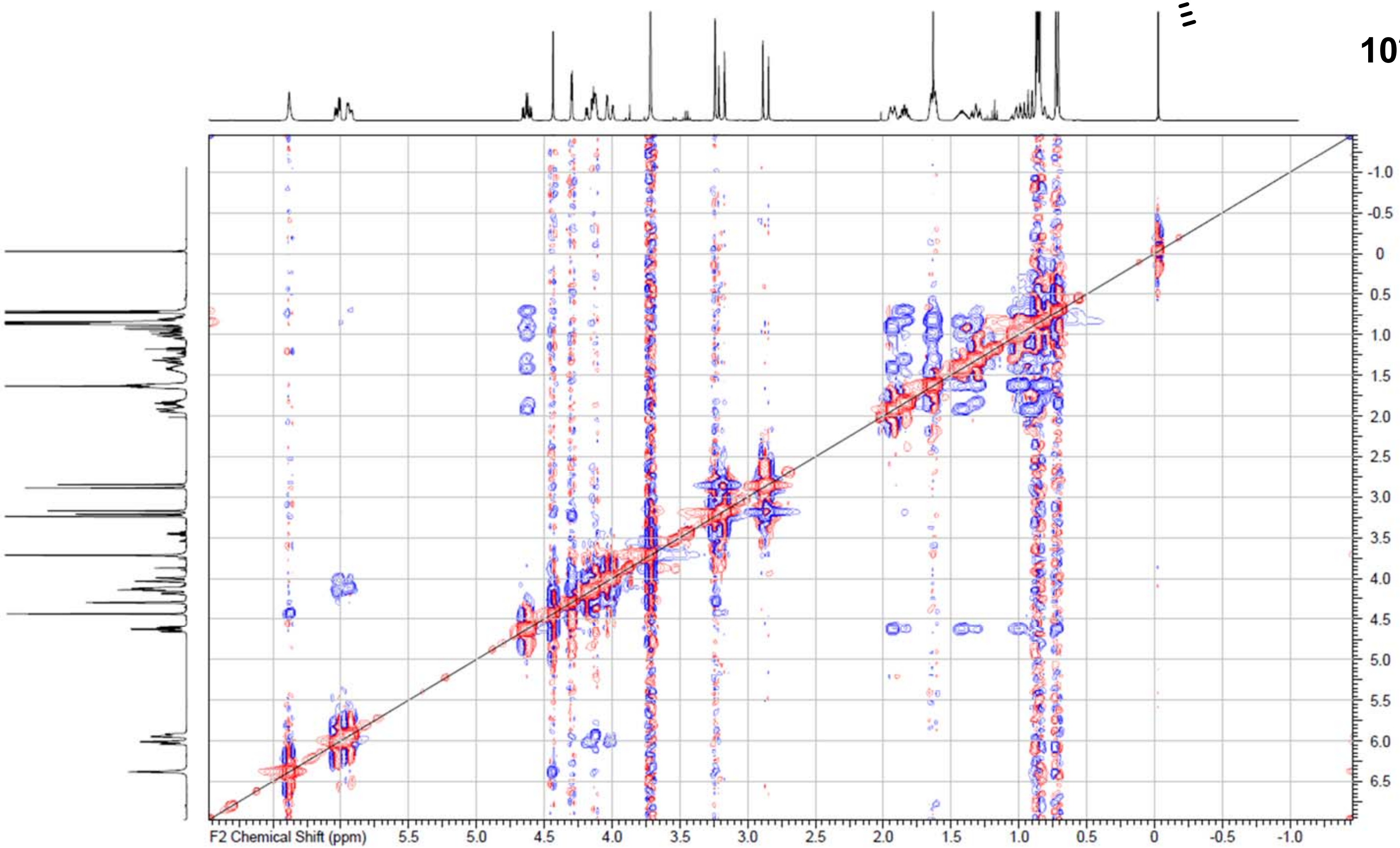

Date 21 Mar 2019 05:53:34
Kenji Morokuma
AA70071-001

Nucleus	(1H, 1H)	Frequency (MHz)	(400.1800, 400.1800)
Pulse Sequence	noesygpphpp_Bruker_AVIII400N	Spectrum Type	NOESY
Solvent	CHLOROFORM-d	Sweep Width (Hz)	(3375.08, 3375.08)

Number of Transients 8
Original Points Count (1024, 256)
Points Count (1024, 1024)

NOESY analysis of **10*** (2*S*) (400 MHz, $CDCl_3$) #2/2

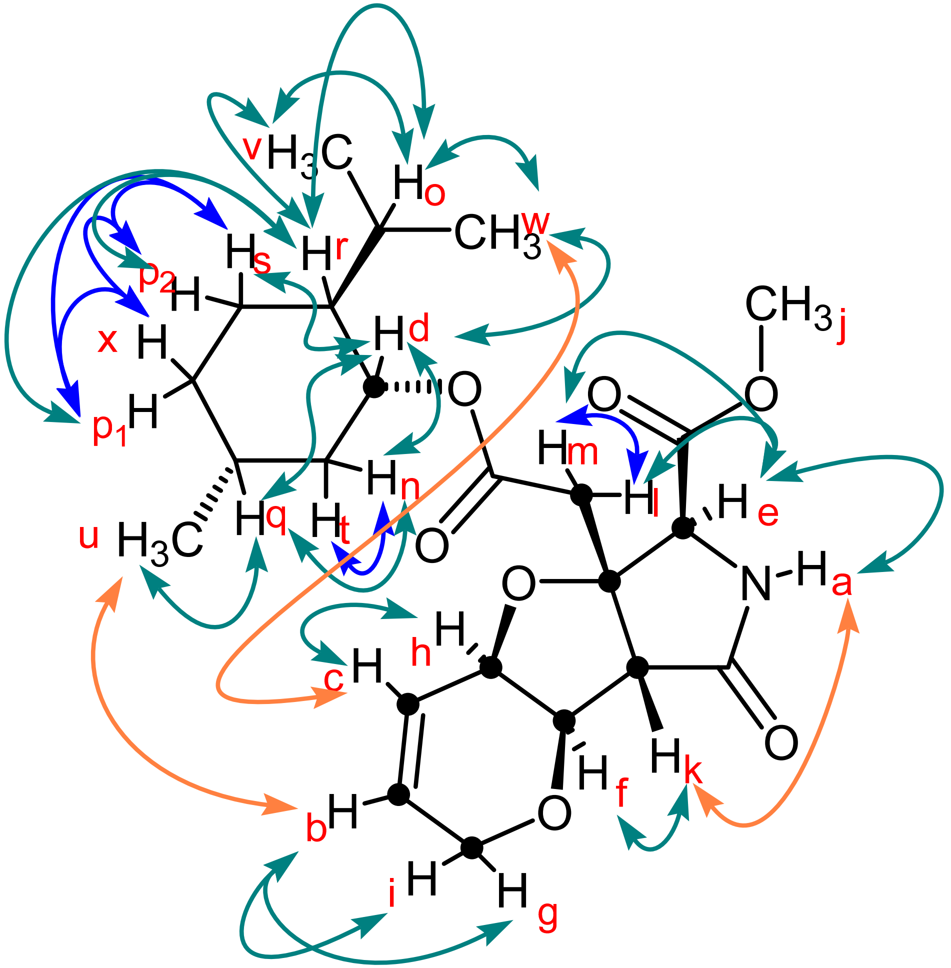

NOE
strong/medium/weak

Assignments and intensities of all NOESY cross peaks observed for **10** (2*R*)

^{1}H NMR for **10** (2*R*) (400 MHz, $CDCl_3$)

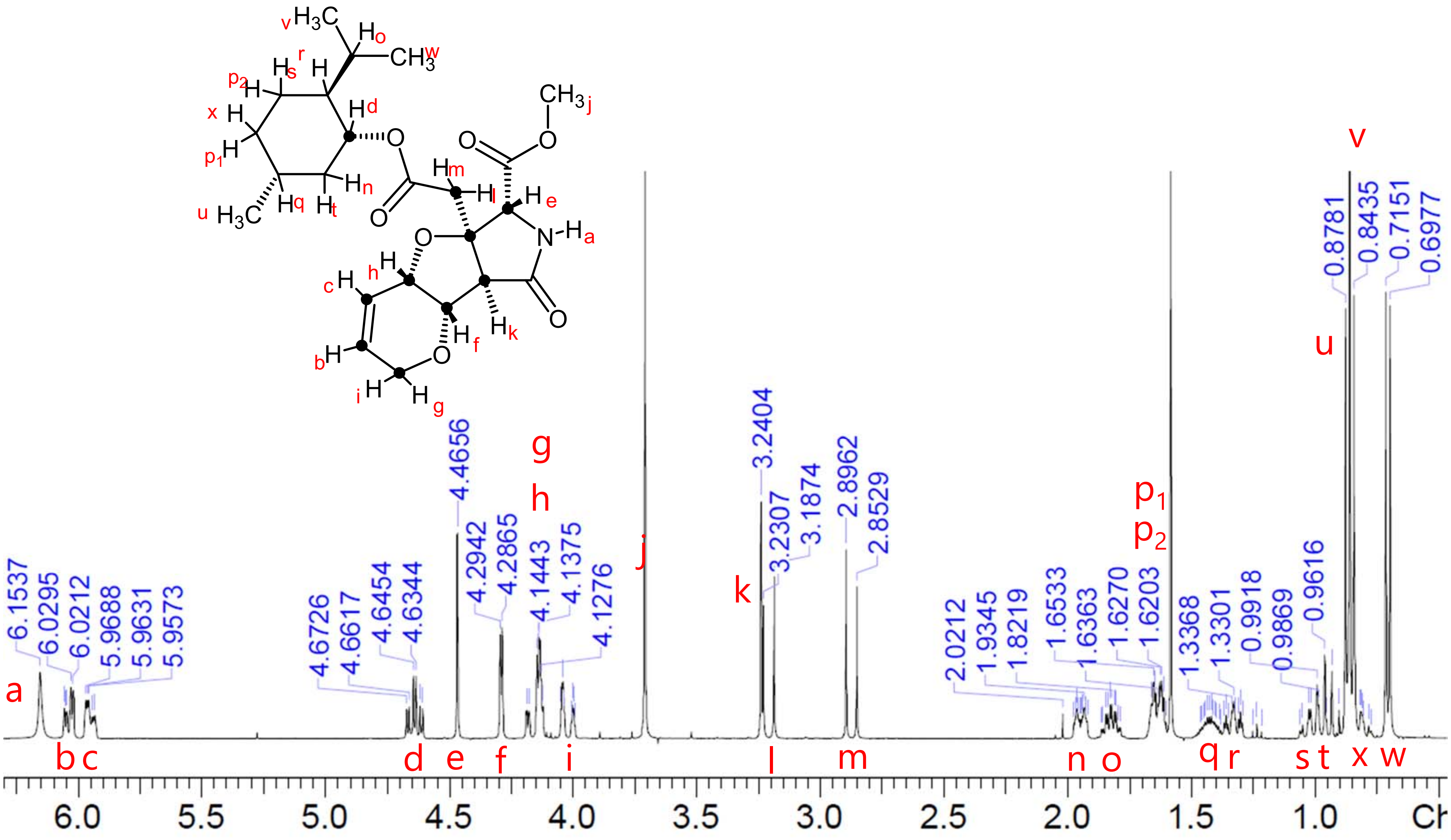

NOESY analysis of **10** (2*R*) (400 MHz, $CDCl_3$) #1/3

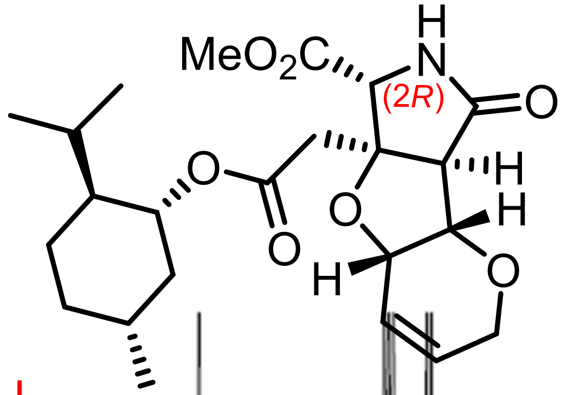

10 (2*R*)

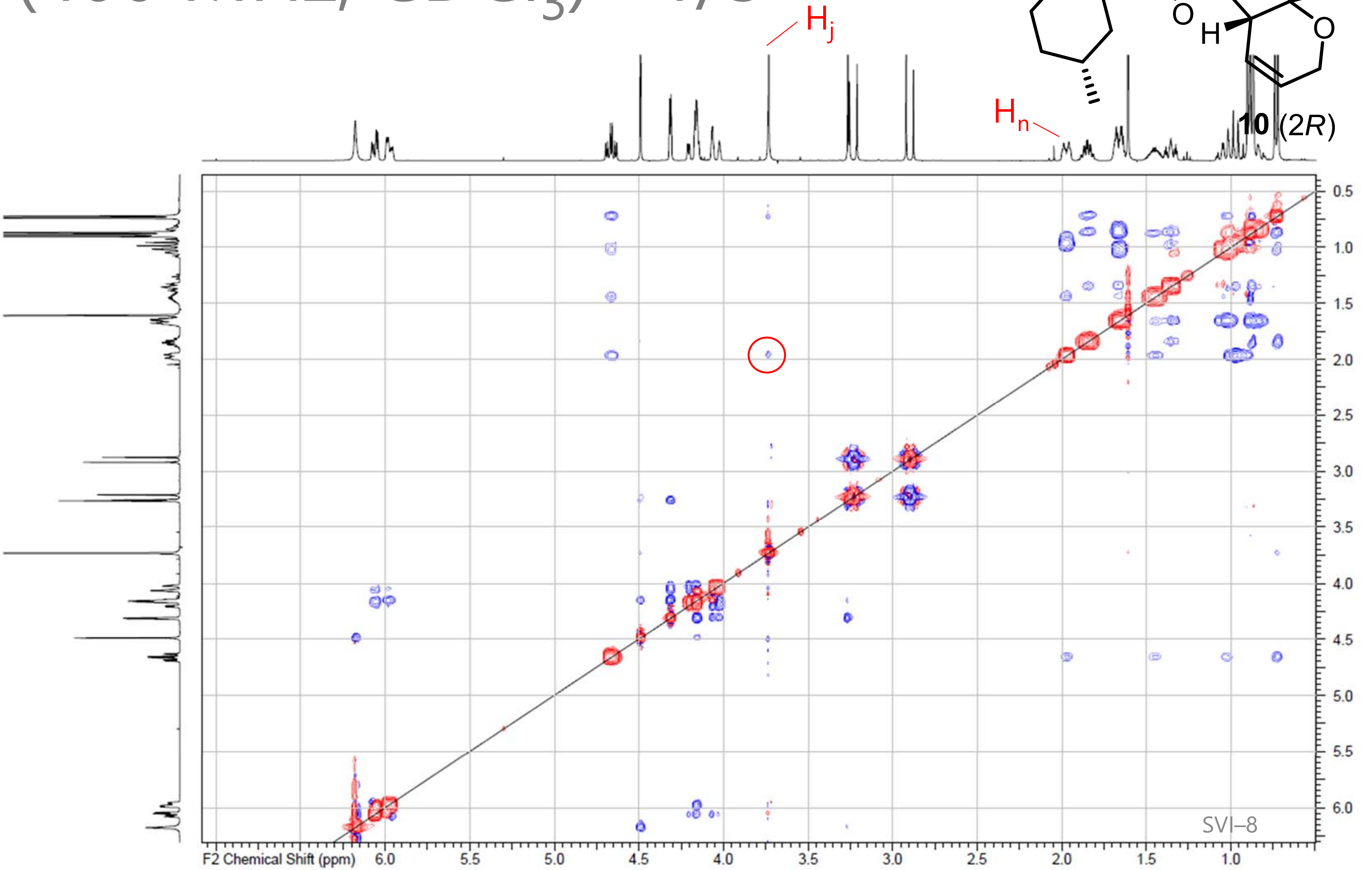

NOESY analysis of **10** (2*R*) (400 MHz, $CDCl_3$) #2/3

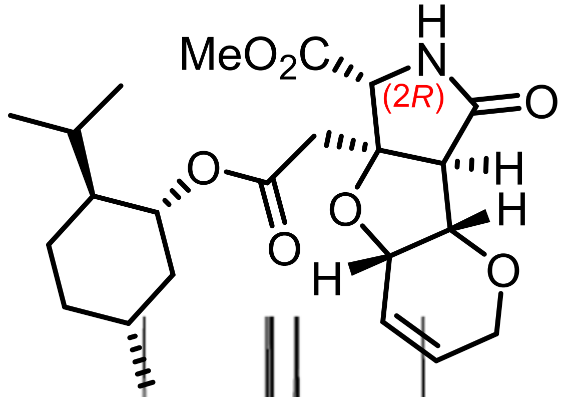

10 (2*R*)

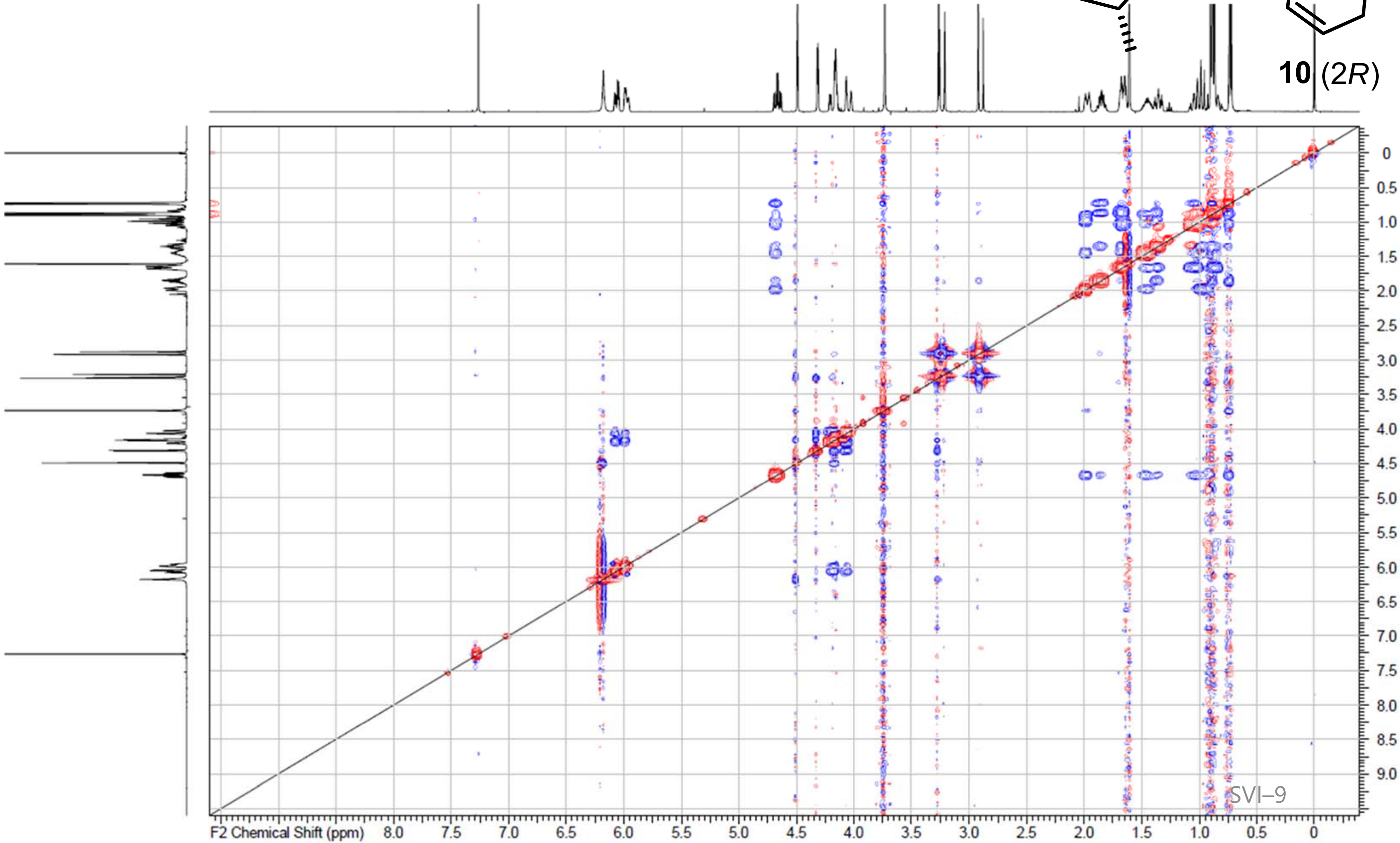

NOESY analysis of **10** (2*R*) (400 MHz, $CDCl_3$) #3/3

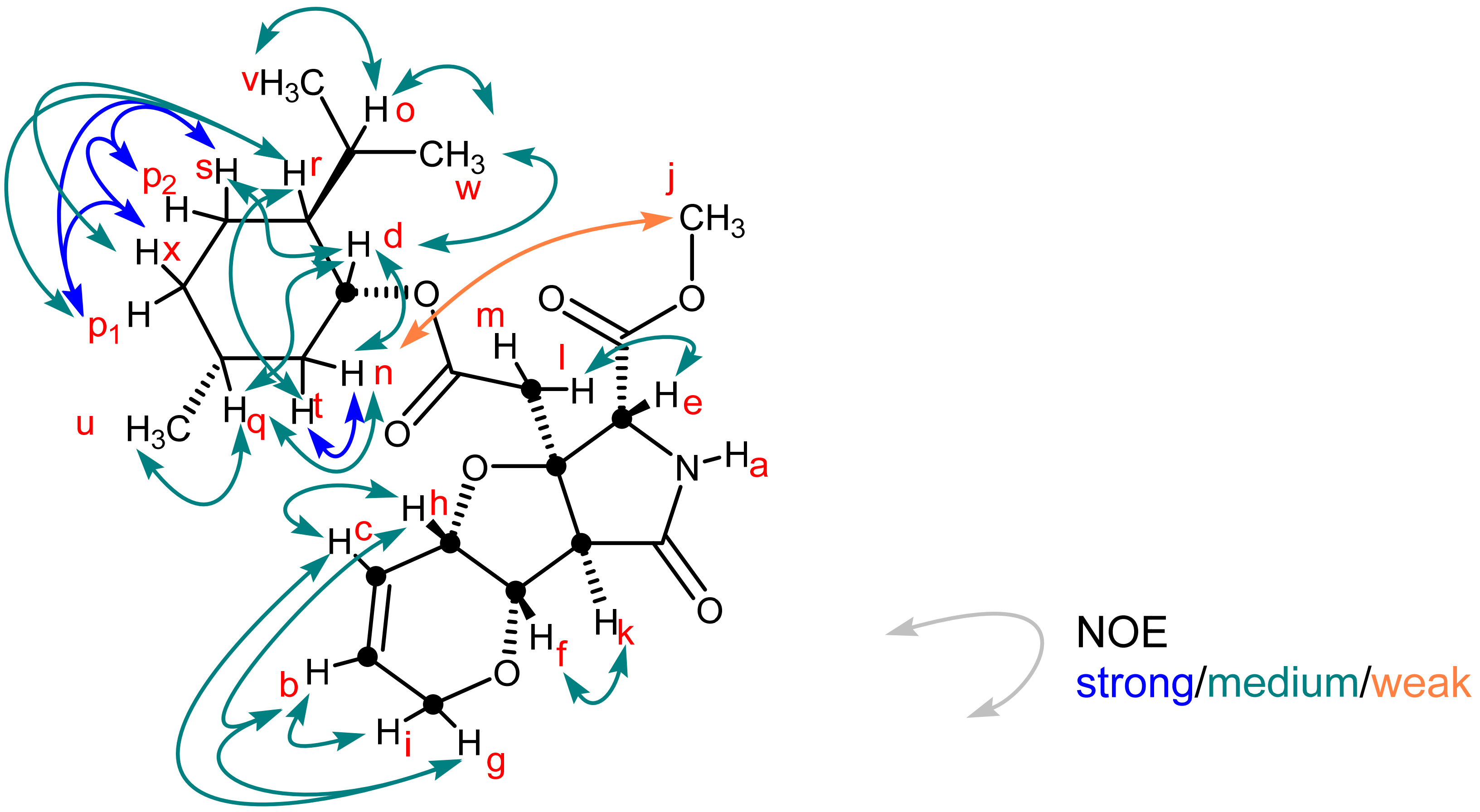

Assignments and intensities of all NOESY cross peaks observed for **21*** (2*S*)

NOESY analysis of **21*** (2*S*) (400 MHz, $CDCl_3$)

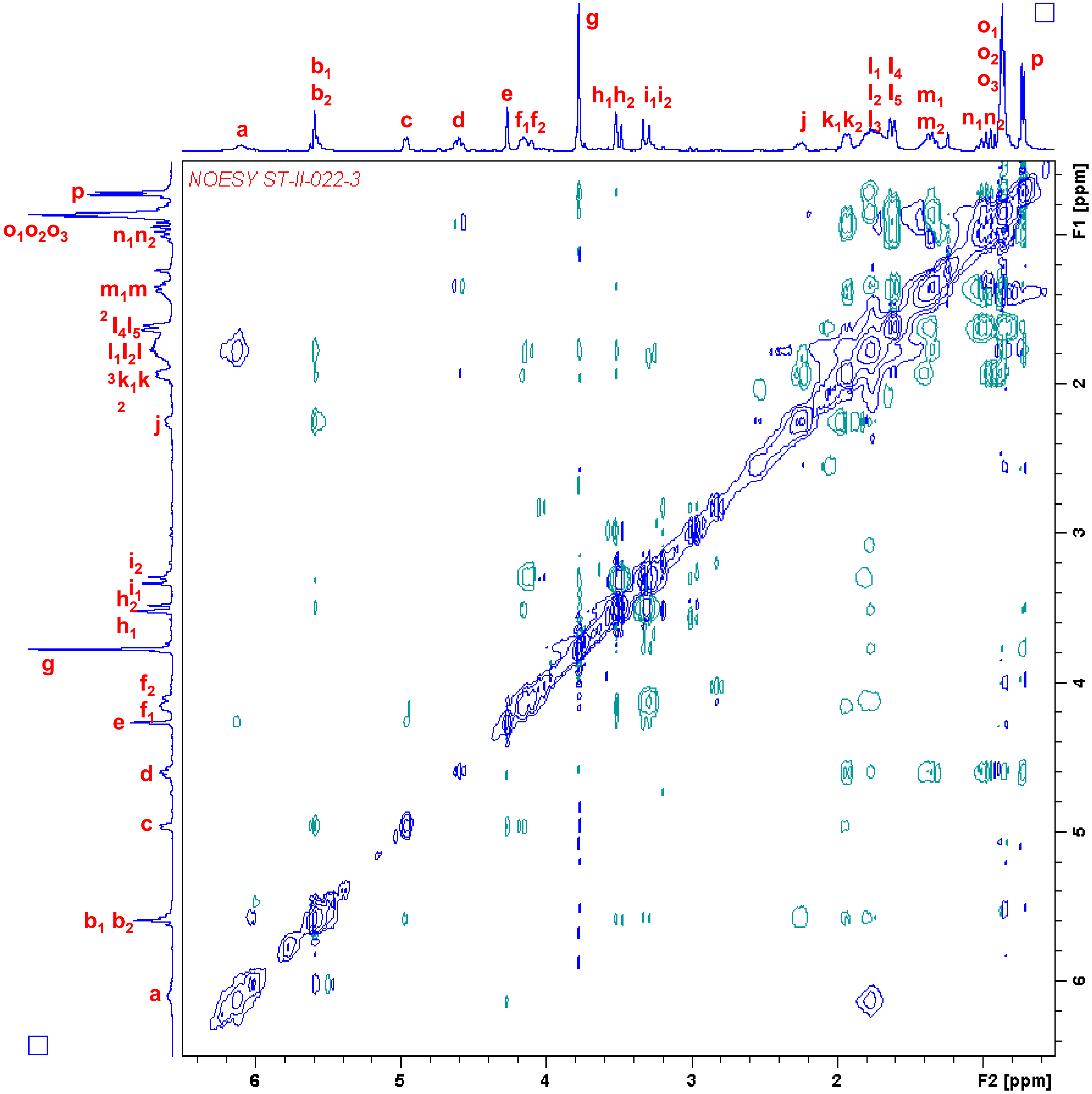

21*(2*S*)

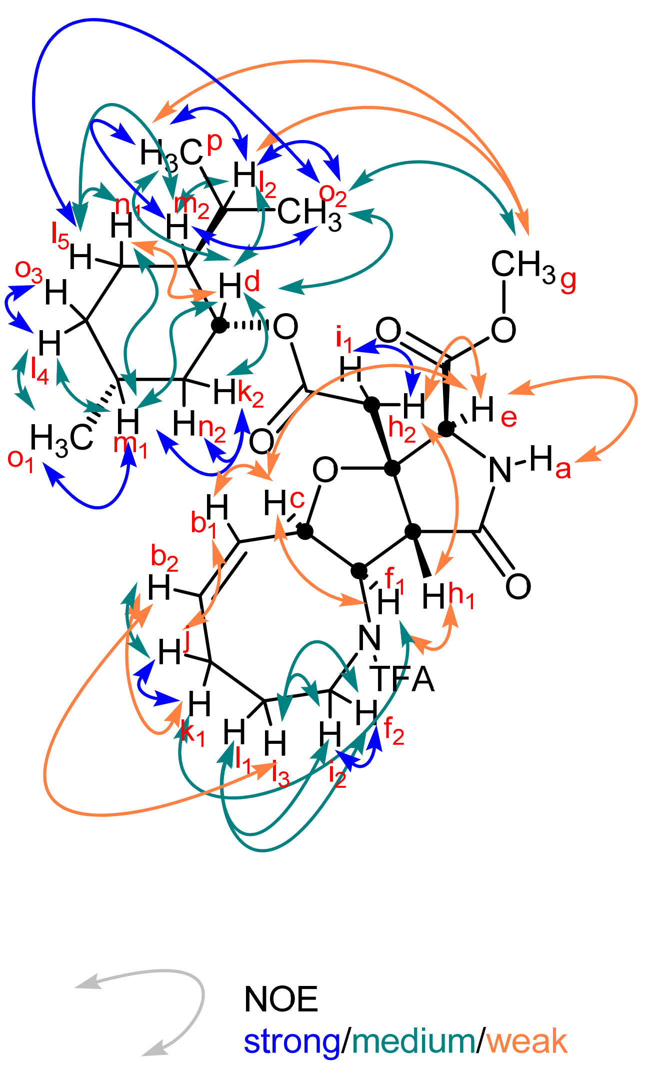

Assignments and intensities of all NOESY cross peaks observed for **21** (2*R*)

NOESY analysis of **21** (2*R*) (400 MHz, $CDCl_3$)

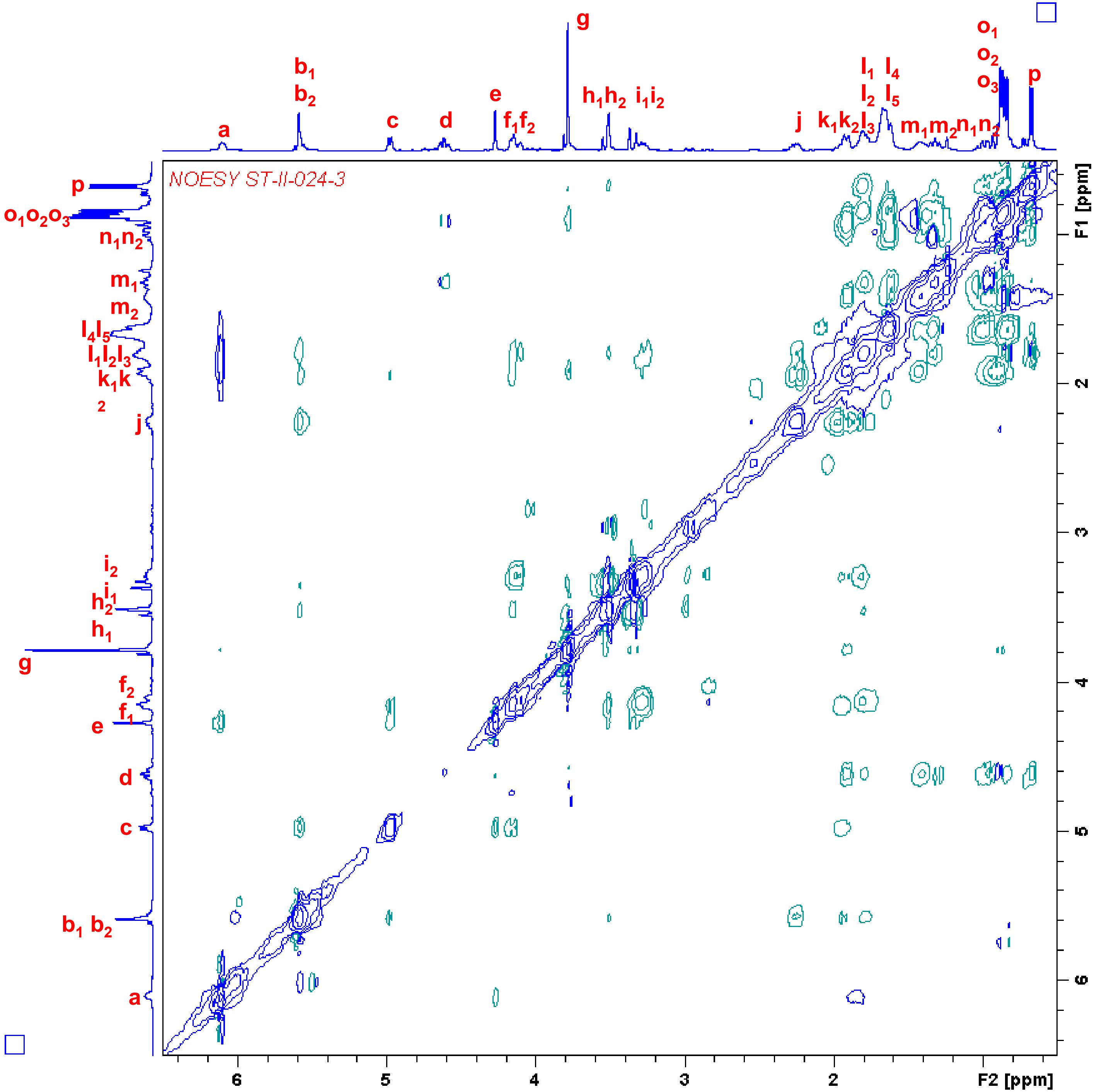

21(2*R*)

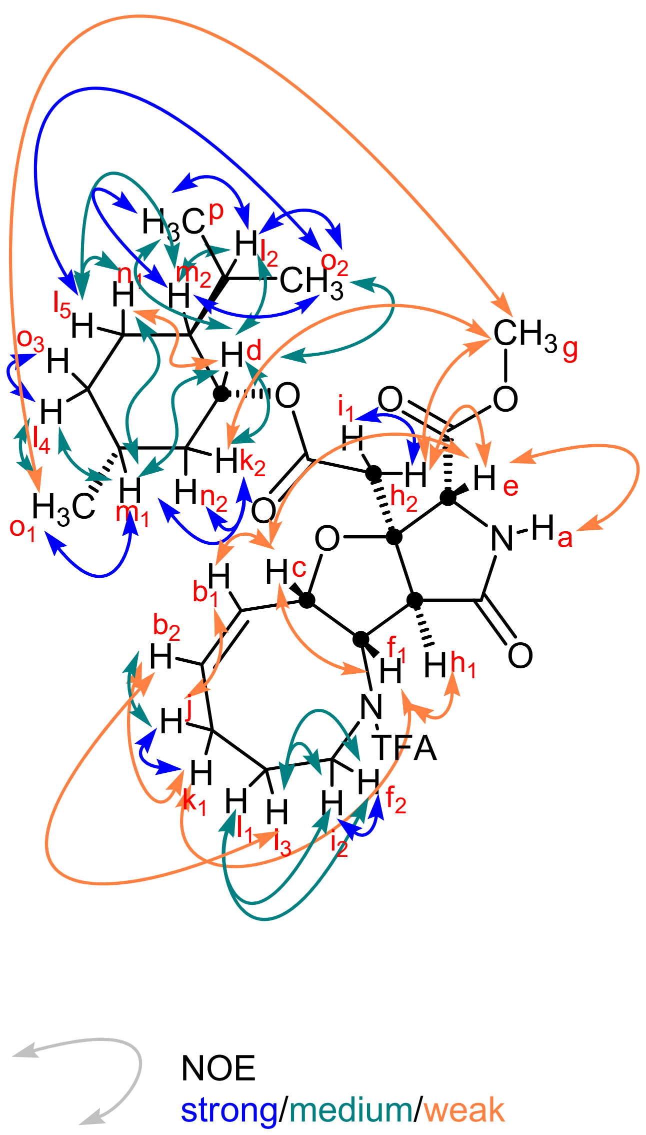

Supplement: File 6 — Assignments and intensities of all NOESY crosspeaks observed for 10*, 10, 21*, and 21. [file Beilstein_J_Org_Chem-17-540-s006.pdf]
