# Supplementary material for: Menthyl esterification allows chiral resolution for the synthesis of artificial glutamate analogs
Source: Beilstein J Org Chem. 2021 Feb 24;17:540–50. doi: 10.3762/bjoc.17.48 (PMC7934734; doi:10.3762/bjoc.17.48)
Supplement: File 7 — Stereochemical analysis of TKM-38 by the PGME amide method, as a support for the original determination of the configuration of menthyl esters 21 and 21* based on NOESY spectra and CONFLEX calculations. [file Beilstein_J_Org_Chem-17-540-s007.pdf]

BEILSTEIN JOURNAL OF ORGANIC CHEMISTRY

Supporting Information

for

Menthyl esterification allows chiral resolution for the synthesis of artificial glutamate analogs

Kenji Morokuma, Shuntaro Tsukamoto, Kyosuke Mori, Kei Miyako, Ryuichi Sakai, Raku Irie and Masato Oikawa

Beilstein J. Org. Chem. **2021,** *17,* 540–550. doi:10.3762/bjoc.17.48

Stereochemical analysis of TKM-38 by the PGME amide method, as a support for the original determination of the configuration of menthyl esters 21 and 21* based on NOESY spectra and CONFLEX calculations

License and Terms: This is a supporting information file under the terms of the Creative Commons Attribution License (https://creativecommons.org/licenses/by/4.0). Please note that the reuse, redistribution and reproduction in particular requires that the author(s) and source are credited and that individual graphics may be subject to special legal provisions.
The license is subject to the *Beilstein Journal of Organic Chemistry* terms and conditions: (https://www.beilstein-journals.org/bjoc/terms)

Contents:

Stereochemical analysis of TKM-38 by the PGME method, as a support for original determination of the configuration of the menthyl esters **21** and **21*** performed based on NOESY spectra and CONFLEX calculations S2–S6

References S7

NOESY analysis of (*R*)-PGME amides S8–S12

$\Delta\delta_H$ analysis of (*R*)-PGME amides S13

Chiral LC-MS analysis of **SVII3**, **SVII4**, and **SVII4*** S14

NMR spectra for (*R*)-PGME amides **SVII1**, **SVII1***, and **SVII2** S15–S21

Stereochemical analysis of TKM-38 by the PGME method, as a support for original determination of the configuration of the menthyl esters 21 and 21* performed based on NOESY spectra and CONFLEX calculations

From *rac*-**19**, diastereomeric (*R*)-PGME amides **SVII1** (t_R = 16.2 min on HPLC) and **SVII1*** (t_R = 10.8 min) were prepared in 26% and 28% yield, respectively (Scheme SVII-1). We then attempted to determine the structures, based on the empirical rule named "the PGME method" [1].

However, the PGME method is generally applied to α,α-disubstituted, α,α,α-trisubstituted, or β,β-disubstituted chiral carboxylic acids [1, 2]. The substrates, **SVII1** and **SVII1*** used in this study are β,β,β-trisubstituted carboxylic acids, and there has been only one example of application to such a substrate [3]. However, in that study, it was shown that the PGME method could be applied by independently analyzing the conformation of the PGME plane in the prepared PGME amides by ROESY. A related discussion can also be found in the original paper by Kusumi et al [1]. Therefore, in this study, the conformational analysis of the PGME plane was first attempted independently by ROESY before conducting the analysis by the PGME method. The results are shown in Figures SVII-1 ~ SVII-3. It was found that NOESY gave better spectra than ROESY for **SVII1** and **SVII1***. In the NOESY spectra of **SVII1** (Figures SVII-1A, 1B, 1C), the presence of five correlations from NH other than NH/PGME(α) supports that the O–C_3–C_{12}–C(=O)–N moiety is in a zigzag conformation (Figure SVII-1D) [4]. Another correlation between PGME aryl proton(s) and CO_2Me is also remarkable to indicate that <u>CO_2Me is positioned at one side of the plane defined by (*R*)-PGME</u> [1, 2]. From the NOESY correlations summarized in Figure SVII-1D, it was also suggested that **SVII1** is the (2*R*)-isomer (Figure SVII-3A). Since these seven NOESY correlations are satisfied in the conformation shown in Figure SVII-3A, **SVII1** is considered to exist mainly in this conformation [5].

In the NOESY spectra of diastereomeric **SVII1*** (Figures SVII-2A and SVII-2B), the correlations at NH/H_{12a}, NH/H_{12b}, and H_{12a}/H_7(H_8) showed that <u>H_{12a} is positioned at one side of the plane defined by (*R*)-PGME</u> [1, 2]. These structural requirements seemed to be well satisfied by the (2*S*)-isomer in

the conformation depicted in Figure SVII-3B; the spatial relationship between NH, H_7, H_8, H_{12a}, and H_{12b} is consistent with the NOESY correlations observed. Although the absent correlations at NH/H_2 and PGME(Ar)/CO_2Me may indicate the relative conformation in the O-C_3-C_{12}-C(=O)-N moiety is slightly twisted compared to **SVII1**, the NOESY correlations observed would indicate the conformation of **SVII1*** is quasi enantiomeric to that of the (2*R*)-isomer **SVII1** (Figure SVII-3A). Since no other important NOESY correlations were observed, the structure shown in Figure SVII-3B seems to be the predominant conformation for **SVII1***.

From NOESY analyses discussed above, it was shown that H_{12a}, H_{12b}, and some protons on the heterotricyclic skeleton are located on either side of the plane defined by (*R*)-PGME in both **SVII1** and **SVII1***, and the protons can be useful for diagnostics to determine the absolute configurations. Thus, from the differences of chemical shift value in ^{1}H NMR between **SVII1** and **SVII1*** ($\Delta\delta = \delta_{SVII1} - \delta_{SVII1*}$) shown in Figure SVII-4, it was determined that **SVII1** is the (2*R*)-isomer, and **SVII1*** is the (2*S*)-isomer. It should be also noted that the conclusion is consistent with the configurations shown in Figure SVII-3, proposed based on NOESY correlations.

Using **SVII1** (2*R*), the protecting groups were attempted to remove (Scheme SVII-1). First, the PMB group was smoothly removed by CAN in 96% yield. However, final hydrolytic deprotection of all other groups under alkaline conditions (1 M KOH, MeOH, 40 °C, 8 d) as used for TKM-38 was not completed, and prolonged reaction (6 d) with higher concentration of KOH (3 M) resulted in decomposition of the substrate.

We next examined acidic hydrolysis (6 M HCl, 110 °C, 1 h) with 0.05 mg (0.09 μmol) of **SVII1** (2*R*), and in this case the reaction was cleanly completed to give **XVII3** as a sole product. HRMS analysis of crude **XVII3** showed MH^+ (*m/z* 311.1237) that seemed to correspond to TKM-38, however, **XVII3** did not match either (2*R*)- and (2*S*)-TKM-38 on chiral HPLC. The observations indicated that the product **XVII3** is a constitutional isomer or a diastereomer of TKM-38.

We then attempted to reproduce the same reaction on menthyl esters **21**

(0.05 mg, 0.09 μmol) and **21*** (0.05 mg, 0.09 μmol). As shown in Scheme SVII-2, acid treatment (6 M HCl, 110 °C, 1 h) of **21** and **21*** gave **SVII4** and **SVII4***, respectively, and both products (**SVII4**, **SVII4***) showed satisfactory MH^+ ions (Scheme SVII-2). As expected, the behaviors of **SVII4** and **SVII4*** on chiral HPLC were obviously different from those of TKM-38 enantiomers, and retention time of **SVII4** was found to be consistent with that of **SVII3** (2*R*) (Figure SVII-5).

From above experiments shown in Schemes SVII-1 and SVII-2, we reasonably concluded that **21** is the enantiomer with 2*R* configuration, and the conclusion strongly supported our original assignment in this study for the menthyl esters **21** and **21*** based on NOESY spectra and CONFLEX calculations.

It should be noted, however, that menthyl esterification is not generally applicable to the configurational analysis of chiral carboxylic acid, from the fact that no other examples have been reported so far. In this study, the bulkiness and the rigidity of the heterotricyclic skeleton of menthyl esters **21** and **21*** would have enabled configurational analysis based on NOESY data and CONFLEX calculations.

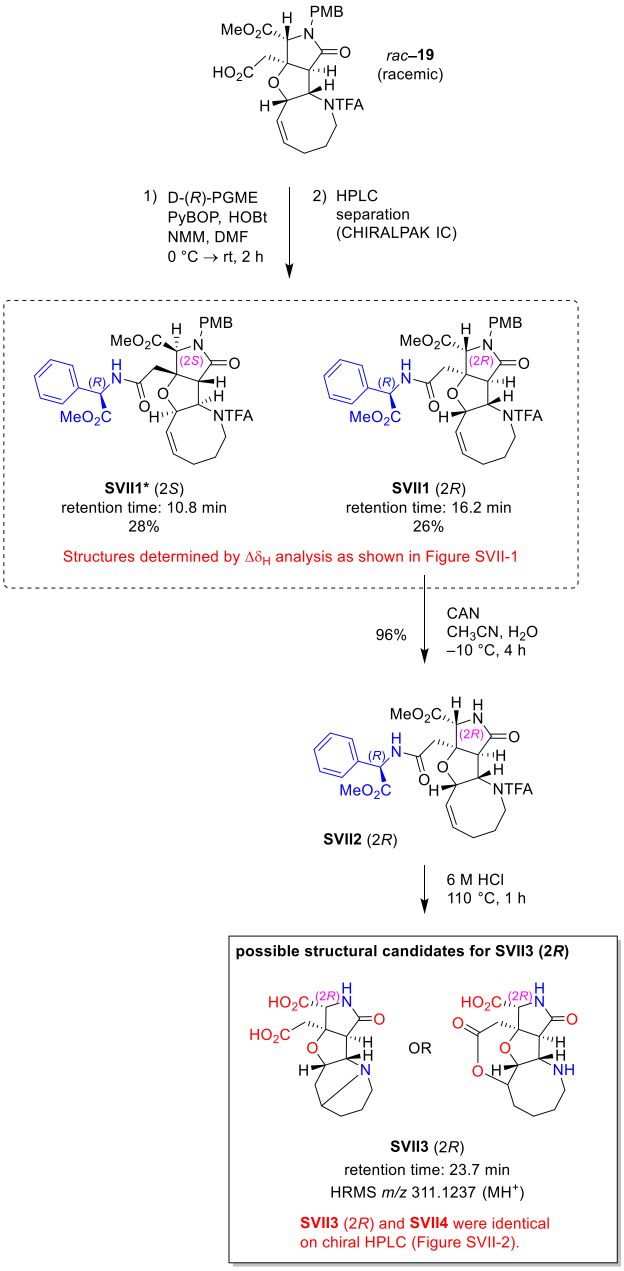

Scheme SVII-1. Preparation from *rac*-**19**, separation, and configurational analysis of (*R*)-PGME amides **SVII1** and **SVII1***, and sequential deprotection toward (2*R*)-**SVII3**.

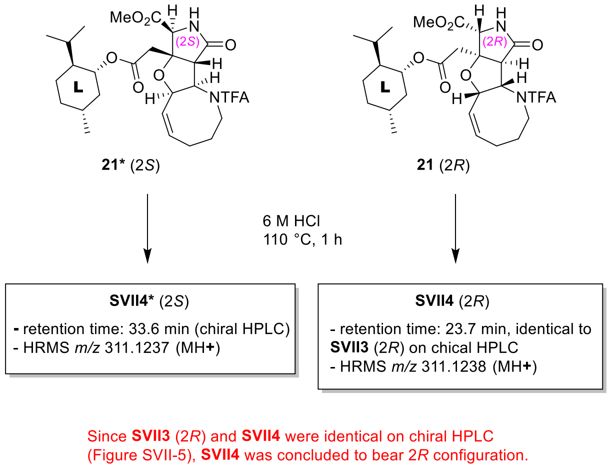

Scheme SVII-2. Preparation of **SVII4** and **SVII4*** from **21** and **21***, respectively, for comparison with (2*R*)-**SVII3**.

References

[1] T. Yabuuchi, T. Kusumi, J. Org. Chem. 65 (2000) 397-404.

[2] T. Kusumi, T. Yabuuchi, H. Takahashi, T. Ooi, J. Synth. Org. Chem., Jpn. 63 (2005) 1102-1114.

[3] G. Pan, Z. Xu, Z. Guo, Hindra, M. Ma, D. Yang, H. Zhou, Y. Gansemans, X. Zhu, Y. Huang, L.-X. Zhao, Y. Jiang, J. Cheng, F. Van Nieuwerburgh, J.-W. Suh, Y. Duan, B. Shen, Proc. Natl. Acad. Sci. U. S. A. 114 (2017) E11131-E11140.

[4] A similar zigzag conformation has been observed in the related paper for S-CR_1R_2-CH_2-C(=O)-N. See reference 3.

[5] The conformation would be thermodynamically favored, since the conformation can be reproduced in conformational search by CONFLEX calculation (data not shown).

NOESY analysis of (*R*)-PGME amides

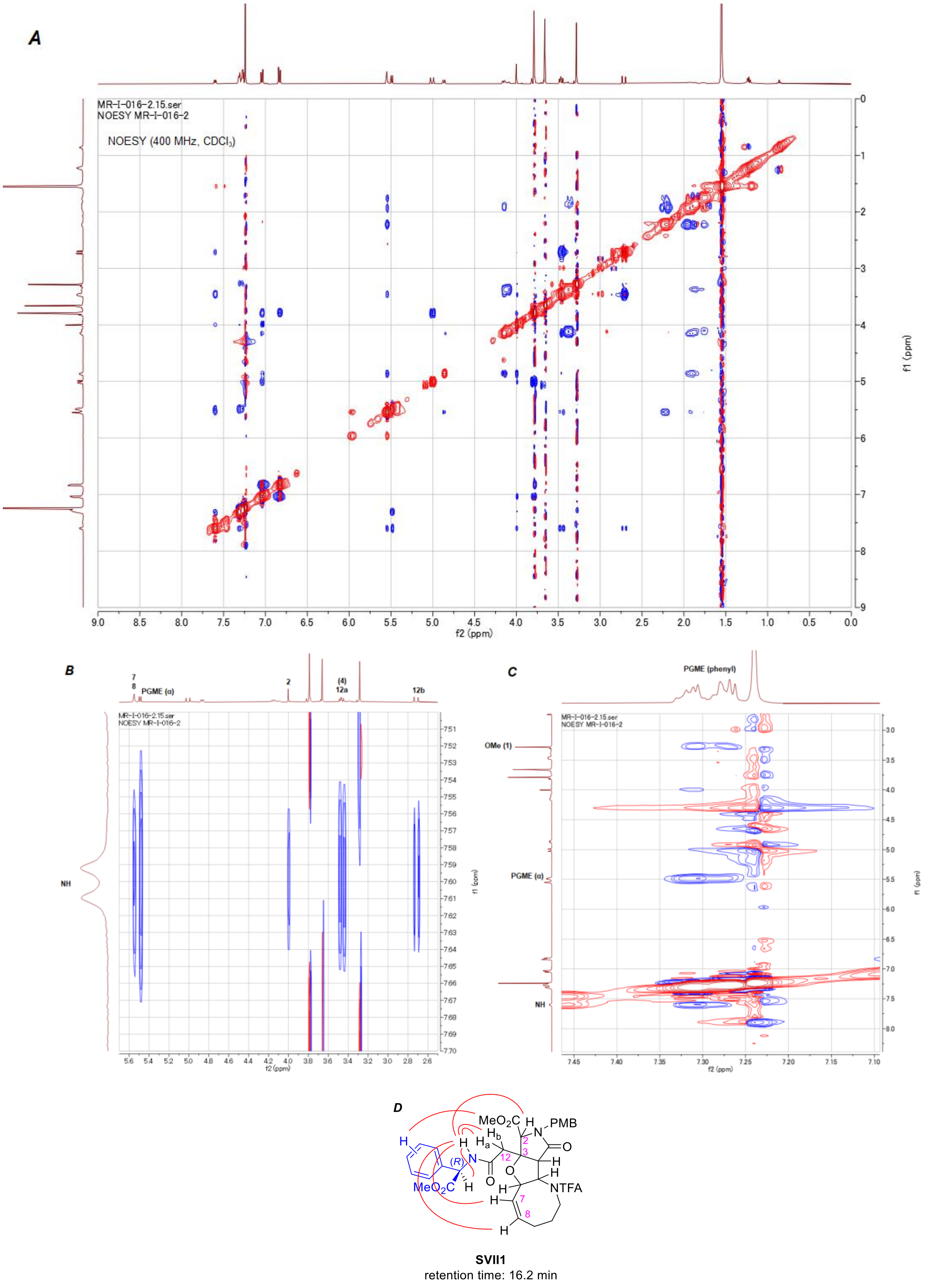

Figure SVII-1. NOESY spectra of (*R*)-PGME amide **SVII1**[a]

[a] H_{12a} denotes one of the geminal protons appearing at the lower field in the ^{1}H NMR spectrum, and H_{12b} denotes the one appearing at the higher field. The purpose of this NOESY analysis was to show the location of the PGME plane in the whole molecule, and hence assignment of H_{12a}/H_{12b} was not necessary. H_{12a} and H_{12b} were, however, later found to be *pro-S* and *pro-R*, respectively, by the PGME method.

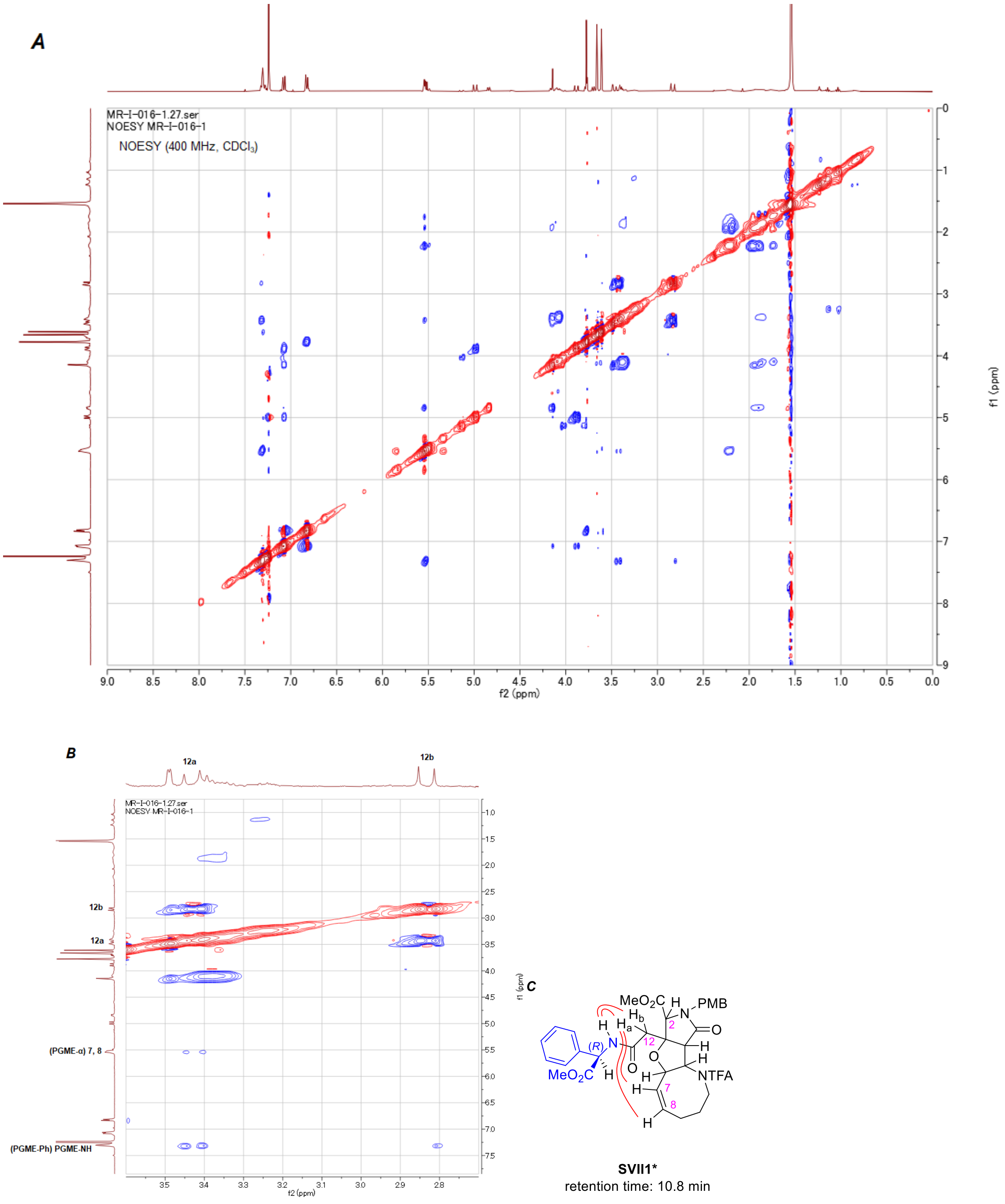

Figure SVII-2. NOESY spectra of (*R*)-PGME amide **SVII1***

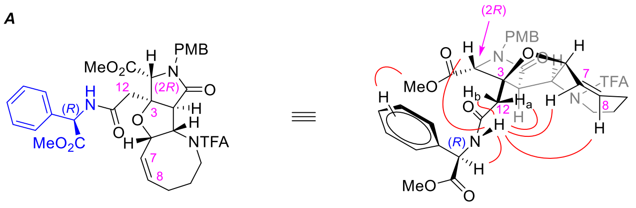

SVII1 (2*R*)
retention time: 16.2 min

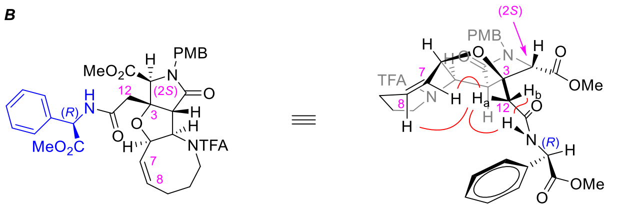

SVII1* (2*S*)
retention time: 10.8 min

Figure SVII-3. Key NOE correlations of (*R*)-PGME derivatives **SVII1** (*A*) and **SVII1*** (*B*), for presumption of the absolute configurations with predominant conformations

$\Delta\delta_H$ analysis of (*R*)-PGME amides

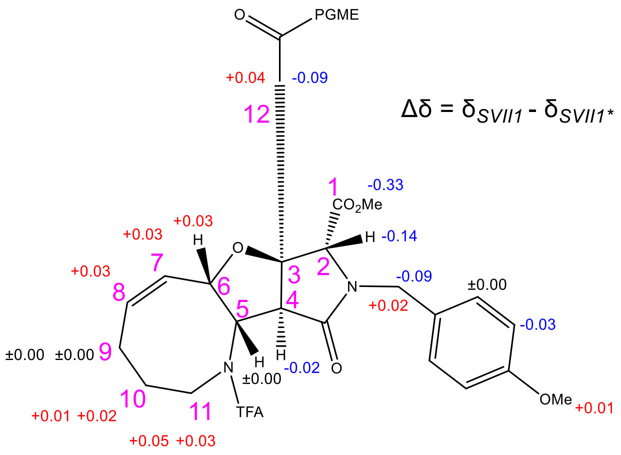

Figure SVII-4. Δδ values obtained for (*R*)-PGME amides **SVII1** and **SVII1*** (400 MHz, $CDCl_3$)

Chiral LC-MS analysis of SVII3, SVII4, and SVII4*

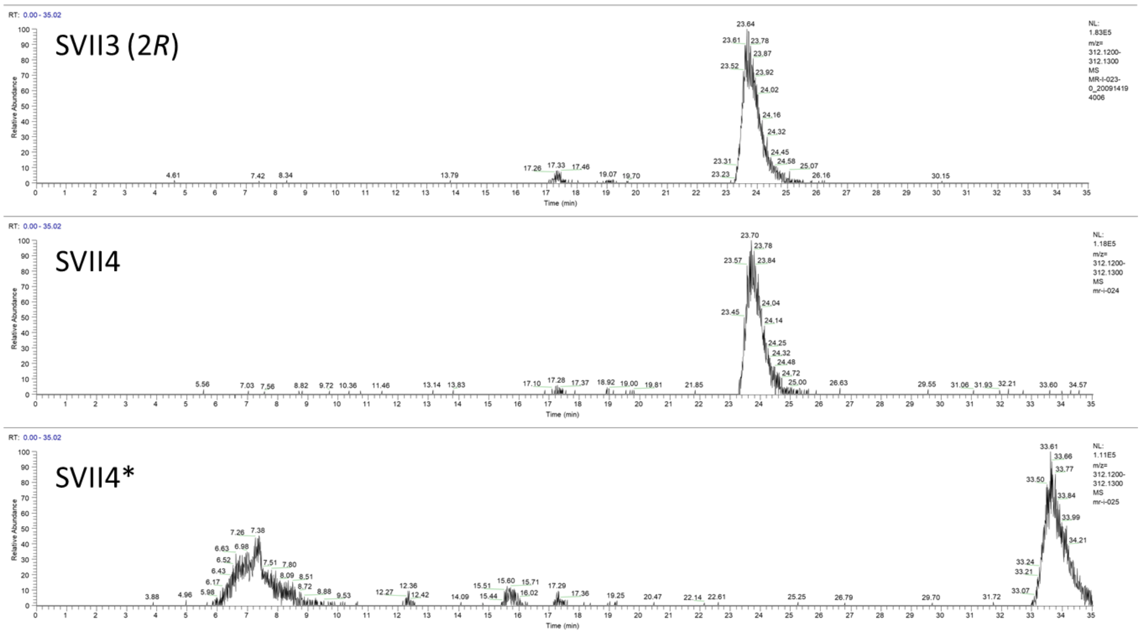

Figure SVII-5. Chiral LC-MS profiles of acid hydrolysates **SVII3, SVII4** and **SVII4***.

Conditions: 4.6 × 250 mm CHIRALPAK ZWIX (+) column, 98% MeOH/H_2O + 25 mM HCO_2H + 25 mM HCO_2NH_4, 0.5 mL/min, 40 °C.

NMR spectra for (*R*)-PGME amides SVII1, SVII1*, and SVII2

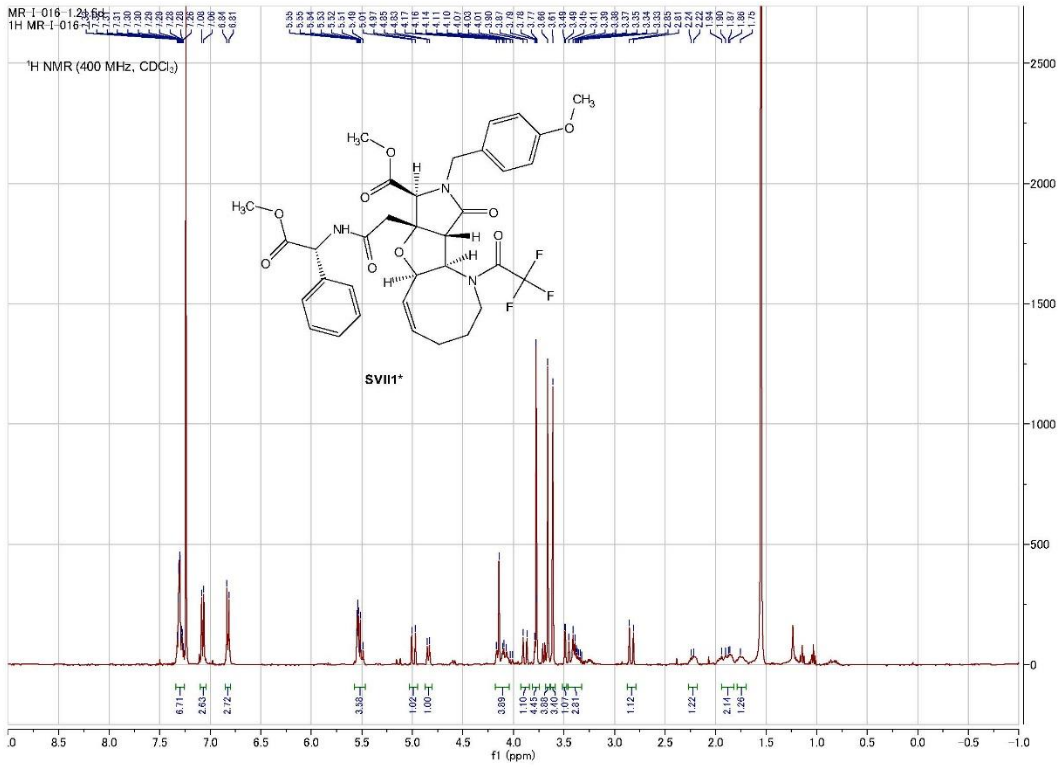

MR I 016 1
1H MR I 016
¹H NMR (400 MHz, CDCl₃)
SVII1*
f1 (ppm)

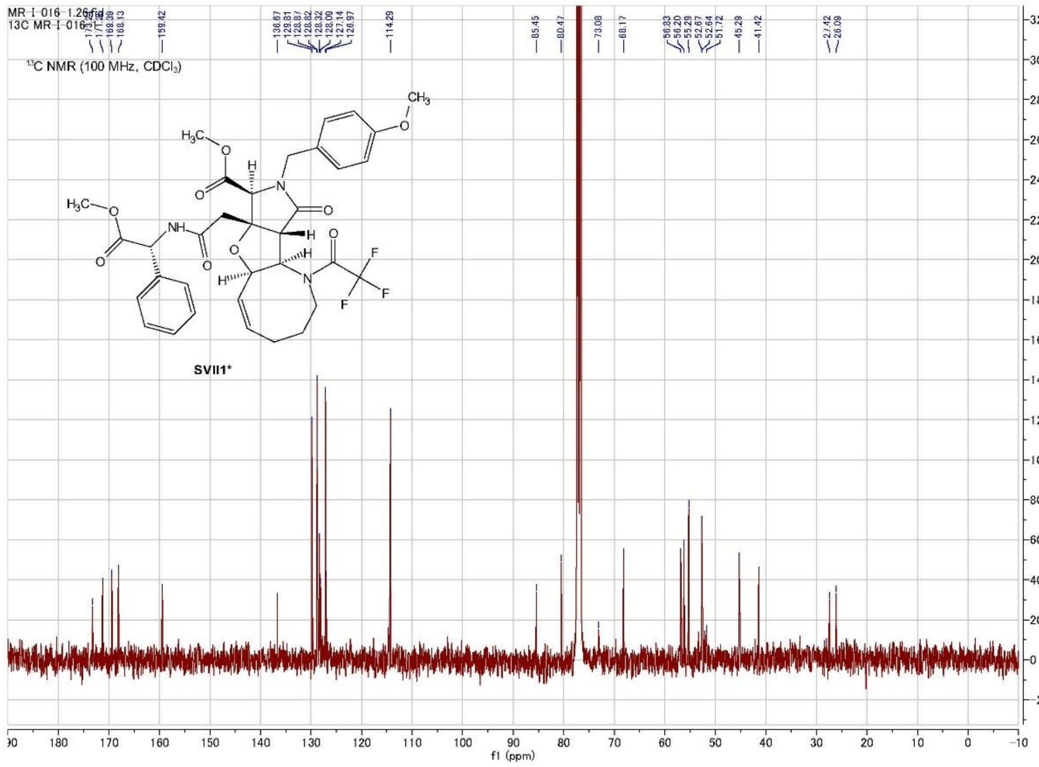

13C NMR (100 MHz, CDCl3)
159.42
136.67
129.81
128.87
128.82
128.32
128.09
127.14
126.97
114.29
85.45
80.47
73.08
68.17
56.83
56.20
55.29
52.67
52.64
51.72
45.29
41.42
27.42
26.09
SVII1*
f1 (ppm)

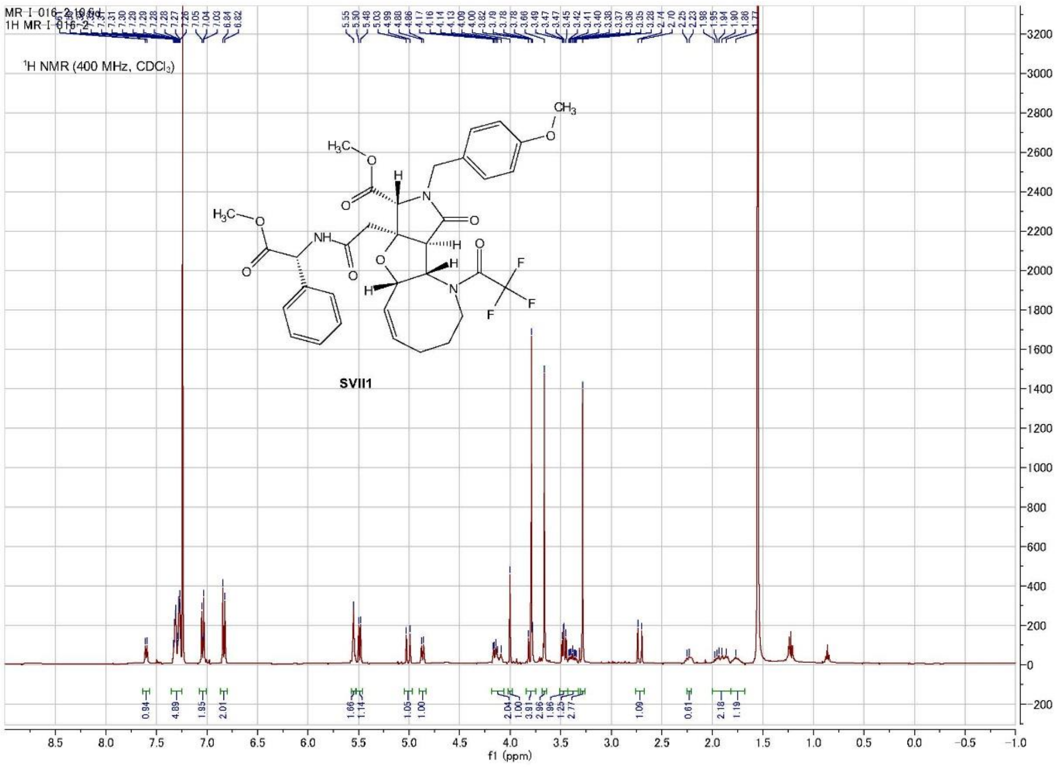

MR I 016-2.10.fid
1H MR I 016-2
¹H NMR (400 MHz, CDCl₃)
SVII1
f1 (ppm)

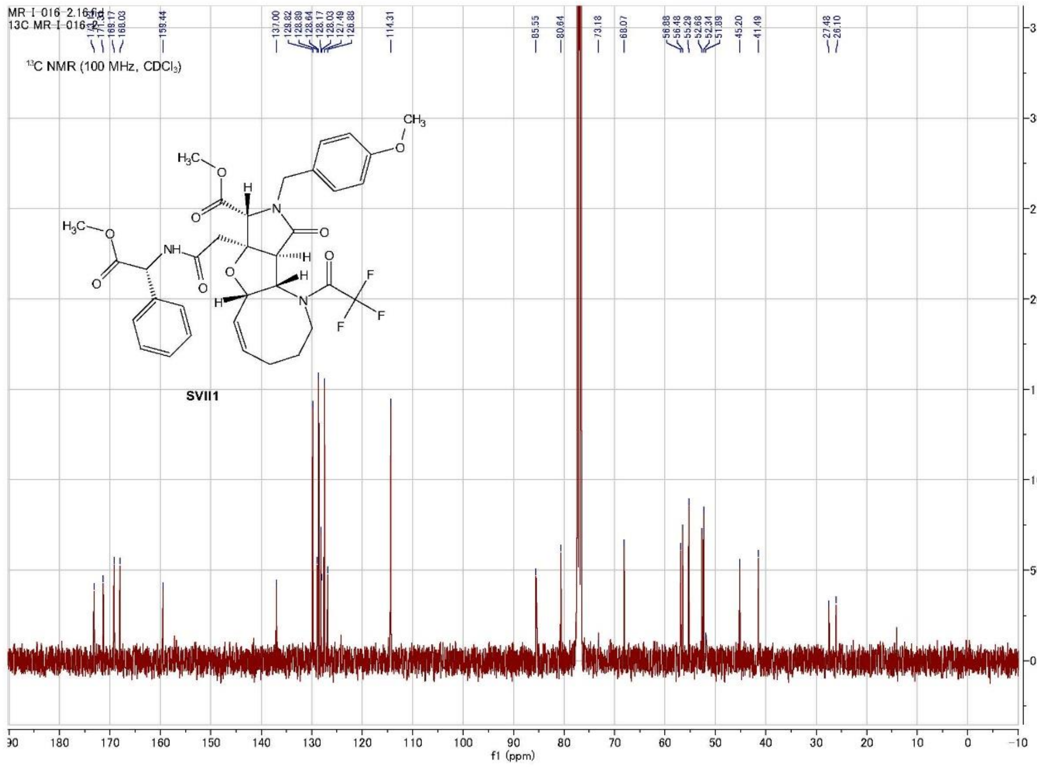

13C NMR (100 MHz, CDCl3)
SVII1
f1 (ppm)

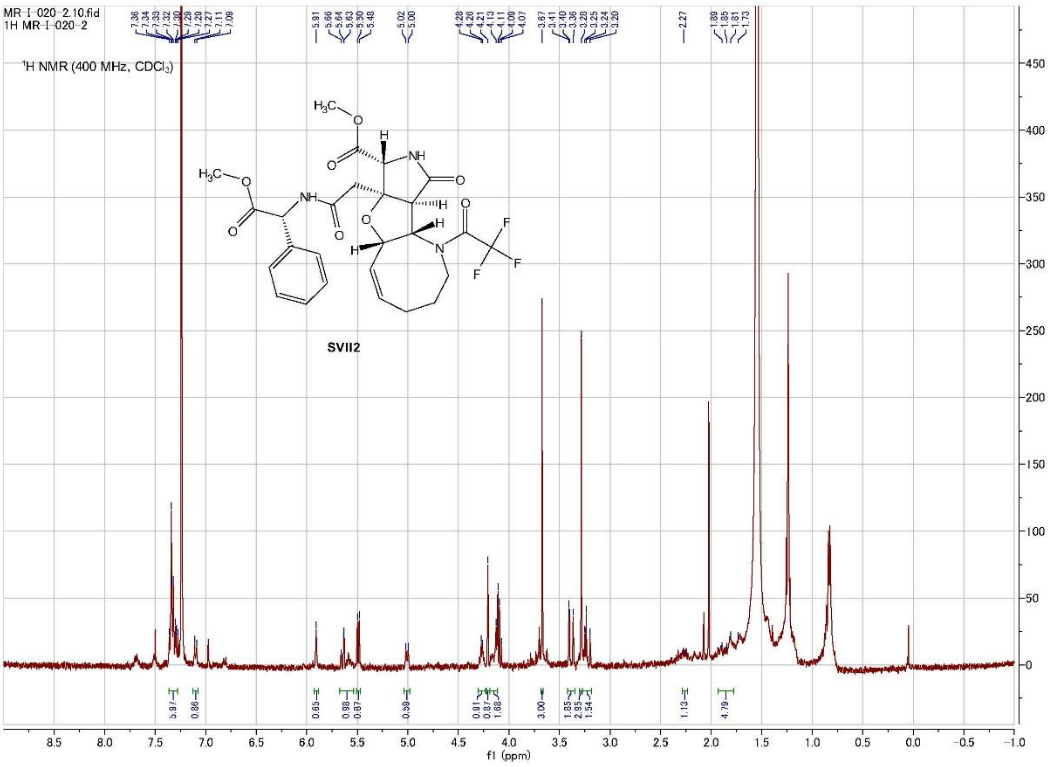
MR-I-020-2.10.fid
1H MR-I-020-2
^{1}H NMR (400 MHz, $CDCl_3$)
SVII2
f1 (ppm)

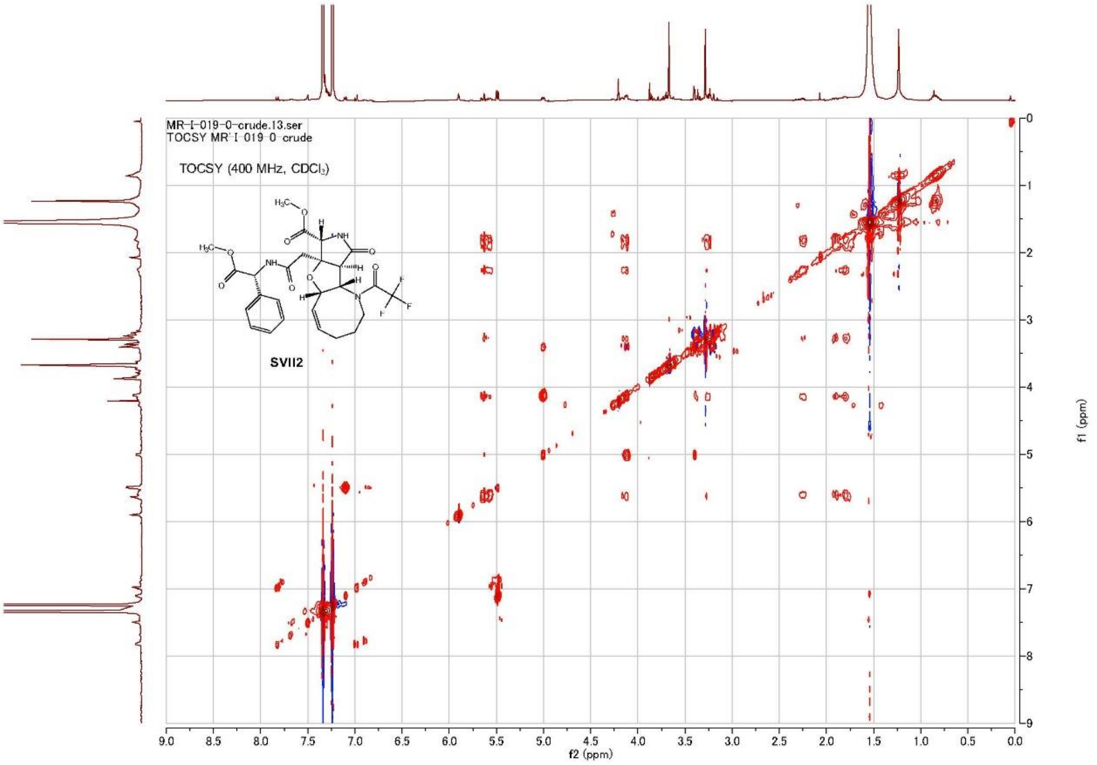
MR-I-019-0-crude.13.ser
TOCSY MR-I-019-0-crude
TOCSY (400 MHz, CDCl$_3$)
SVII2
f2 (ppm)
f1 (ppm)
